# Efficacy of Novel Quaternary Ammonium and Phosphonium Salts Differing in Cation Type and Alkyl Chain Length against Antibiotic-Resistant *Staphylococcus aureus*

**DOI:** 10.3390/ijms25010504

**Published:** 2023-12-29

**Authors:** Bárbara Nunes, Fernando Cagide, Carlos Fernandes, Anabela Borges, Fernanda Borges, Manuel Simões

**Affiliations:** 1 LEPABE—Laboratory for Process Engineering, Environment, Biotechnology and Energy, Faculty of Engineering, University of Porto, Rua Dr. Roberto Frias, s/n, 4200-465 Porto, Portugal; up201804372@edu.fe.up.pt (B.N.); apborges@fe.up.pt (A.B.); 2 ALiCE—Associate Laboratory in Chemical Engineering, Faculty of Engineering, University of Porto, Rua Dr. Roberto Frias, 4200-465 Porto, Portugal; 3 CIQUP-IMS, Department of Chemistry and Biochemistry, Faculty of Sciences, University of Porto, Rua do Campo Alegre, s/n, 4169-007 Porto, Portugalcarlos.fernandes@fc.up.pt (C.F.); fborges@fc.up.pt (F.B.)

**Keywords:** antibacterial activity, cytotoxicity profile, quaternary ammonium and phosphonium compounds, *Staphylococcus aureus*, structure–activity relationship

## Abstract

Antibacterial resistance poses a critical public health threat, challenging the prevention and treatment of bacterial infections. The search for innovative antibacterial agents has spurred significant interest in quaternary heteronium salts (QHSs), such as quaternary ammonium and phosphonium compounds as potential candidates. In this study, a library of 49 structurally related QHSs was synthesized, varying the cation type and alkyl chain length. Their antibacterial activities against *Staphylococcus aureus*, including antibiotic-resistant strains, were evaluated by determining minimum inhibitory/bactericidal concentrations (MIC/MBC) ≤ 64 µg/mL. Structure–activity relationship analyses highlighted alkyl-triphenylphosphonium and alkyl-methylimidazolium salts as the most effective against *S. aureus* CECT 976. The length of the alkyl side chain significantly influenced the antibacterial activity, with optimal chain lengths observed between C_10_ and C_14_. Dose–response relationships were assessed for selected QHSs, showing dose-dependent antibacterial activity following a non-linear pattern. Survival curves indicated effective eradication of *S. aureus* CECT 976 by QHSs at low concentrations, particularly compounds **1e**, **3e**, and **5e**. Moreover, in vitro human cellular data indicated that compounds **2e**, **4e**, and **5e** showed favourable safety profiles at concentrations ≤ 2 µg/mL. These findings highlight the potential of these QHSs as effective agents against susceptible and resistant bacterial strains, providing valuable insights for the rational design of bioactive QHSs.

## 1. Introduction

Over the past half-century, the excessive or inadequate use of antibiotics has accelerated the emergence of multidrug-resistant (MDR) bacteria [1,2,3]. Such a medical insurgence could compromise the efficacy of currently available antibiotics, posing a major threat to public health [4]. According to a World Health Organization (WHO) report, at least 700,000 people globally die each year from antibiotic-resistant bacterial infections [5,6]. In 2019, the United States reported more than 2.8 million cases of antibiotic-resistant bacterial infections, resulting in over 35,000 deaths [5], while Europe witnessed about 33,000 annual deaths attributed to antibiotic-resistant infections [7]. *Staphylococcus aureus*, recognized as a leading cause of human bacterial infections globally, exacerbates the issue with the yearly emergence of methicillin-resistant *S. aureus* (MRSA) strains exhibiting high resistance rates [8]. In 2017, MRSA alone contributed to an estimated 323,700 cases in hospitalized patients in the United States, causing 10,600 deaths and incurring £1.7 billion in healthcare costs [9,10,11]. Hence, addressing the ongoing challenge of developing novel antibacterial agents to combat multidrug resistance, particularly against MDR *S. aureus*, remains an imperative task.

Quaternary heteronium salts (QHSs), such as quaternary ammonium and phosphonium compounds, present a promising avenue for discovering new antibiotics to counter antibacterial resistance [6,12,13,14,15]. Since the 1930s, quaternary ammonium salts (QASs) have been integral components of various antiseptics and disinfectants [6,16,17,18,19], finding applications in household, agriculture, medicine, and industry [16,17,19]. The structure of QASs comprises a positively charged nitrogen atom (head) linked through four bonds to alkyl or aryl chains (tails) with varying numbers of carbon atoms [16,17,18]. Depending on their structure, they exhibit antimicrobial activity against Gram-positive bacteria, fungi, and enveloped viruses [16]. Despite extensive research, the precise mechanism of action of QASs is not yet fully elucidated [6,16,20]; however, it is widely acknowledged that they interact with cell membranes [16,21]. Selectivity is thought to rely on differences between pathogenic and mammalian membranes, with the former abundant in negatively charged components and the latter in zwitterionic ones. Positively charged QASs interact with negatively charged membrane components, such as phospholipids, inducing their disintegration. In contrast, mammalian cell membranes, predominantly composed of zwitterionic lipids (e.g., phosphatidylcholine), demonstrate diminished interactions with QASs [21]. Additionally, some studies indicate a correlation between membrane activity and the hydrophobicity of the active molecule, as well as the density of the cationic charge [6,12].

Discovered in the mid-20th century, quaternary phosphonium salts (QPSs) have been extensively researched for their antimicrobial properties and safety in human applications [6,12,15,22]. These salts, which share structural similarities with QASs, demonstrate high efficiency across various biological activities, serving as anticholinesterase, anticancer, antitumor, antiviral, analgesic, antibacterial, and antiparasitic agents [22,23]. Notably, they display useful antibacterial properties against both Gram-positive and Gram-negative bacteria [15,22,23,24]. The reported killing activities extend to viruses, parasites, and fungi [23,24]. In a study by Li et al. [23,25], a novel category of quaternary phosphonium *N*-chloramine was reported, formed by covalently linking the *N*-chloramine moiety and the QPS through varying aliphatic methylene chains. These salts exhibited significant antibacterial activity against *Escherichia coli* and *S. aureus*, with efficacy varying based on the length of the methylene chain linker. The antibacterial action declined as the methylene linker increased from –(CH_2_)_3_– to –(CH_2_)_8_–, while the highest log reduction was achieved with the introduction of the –(CH_2_)_12_– linker. In another study, Khasiyatullina et al. [23,26] synthesized a series of functionally substituted tetraarylphosphonium salts with hydroxy- and methoxy-moieties. These salts demonstrated activity against Gram-positive bacteria such as *S. aureus* and *Bacillus cereus*. It was observed that the increase in the number of phosphonium salt methoxy substituents enhanced bacteriostatic and bactericidal activity. Additionally, Ravindra and Karpagam [23,27] explored the use of various azine heterocycles in the formation of phosphonium salts, including pyrazine, quinoxaline, and quinoline. The biological activity analysis revealed strong interaction and remarkable antibacterial characteristics against both Gram-positive bacteria (*S. aureus*, *Bacillus subtilis*) and Gram-negative bacteria (*E. coli*, *Klebsiella pneumoniae*). Significantly, the quinoline-functionalized phosphonium salt exhibited higher antibacterial activity compared to those with pyrazine and quinoxaline groups. 

While QHSs have exhibited antimicrobial activity against a broad spectrum of pathogens, as evidenced in various antimicrobial susceptibility testing protocols, assessing their potential as antibacterial agents faces challenges due to the lack of comparative data [28]. This complexity is exacerbated by the diverse methods and procedures employed in antimicrobial susceptibility testing over the years [28,29]. Consequently, there is an urgent need for a systematic examination of structure–activity relationships and the development of a strategy to fine-tune biological properties through the structural modification of active molecules [12]. The optimization of potential antimicrobial agents is critically dependent on achieving a delicate balance between antimicrobial activity and cytotoxicity [12]. Compounds with potent antimicrobial effects face approval challenges if they demonstrate high cytotoxicity to healthy eukaryotic cells [12,30]. For instance, Ermolaev et al. [12] investigated the antimicrobial, hemolytic, and cytotoxic activities of QPSs based on tri-*tert*-butylphosphine. The results indicated that tri-*tert*-butyl(*n*-dodecyl)phosphonium and tri-*tert*-butyl(*n*-tridecyl)phosphonium bromides exhibited both low cytotoxicity against human cells and high antimicrobial activity against bacteria, including MRSA. Consequently, these compounds were deemed safe for human health and demonstrated high selectivity for pathogenic microorganisms, positioning them as promising candidates for drug development.

**Figure 1:**
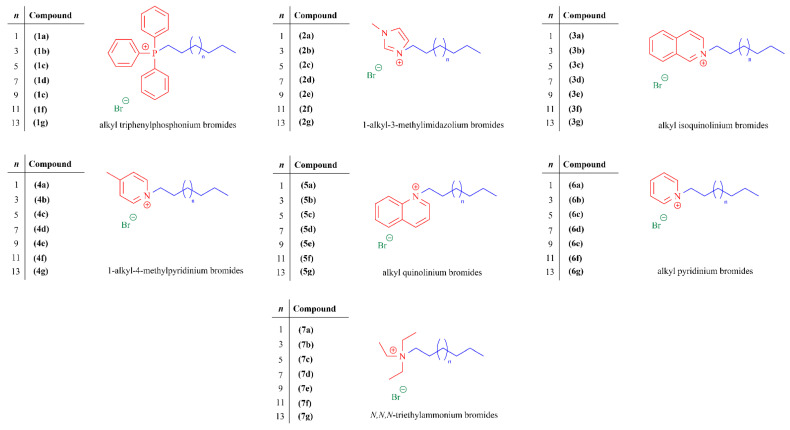
General chemical structure of quaternary ammonium and phosphonium salts synthesized and used in this study.

**Scheme 1:**
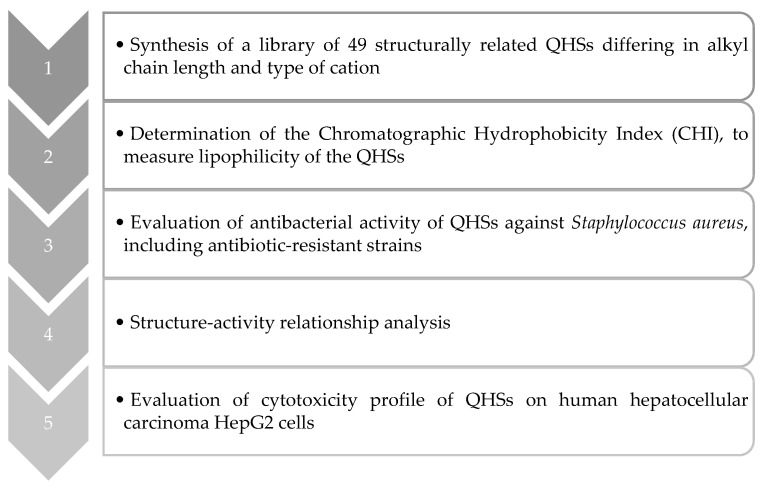
Overview of the rational design employed in this study.

In this study, a library of 49 structurally related QHSs (alkyl-substituted cations with bromide anions) (Figure 1) was designed, synthesized, and assessed for their antibacterial activity against *S. aureus*, including antibiotic-resistant strains. Furthermore, an evaluation of their cytotoxicity profile against hepatocellular carcinoma (HepG2) cells was conducted. Structural modifications were performed on the nature of the cation (triphenylphosphonium, methylimidazolium, isoquinolinium, quinolinium, methylpyridinium, pyridinium, and triethylammonium) and on the length of the alkyl linear chain (six-, eight-, ten-, twelve-, fourteen-, sixteen-, and eighteen-carbon atoms). A visual representation of the rational design employed in this study is presented in Scheme 1. 

The 49 compounds synthesized and tested in the present work were chosen as representatives of different classes of QHSs which have attracted a growing interest in recent years. These compounds featured alkyl chain lengths ranging from C_6_ to C_18_, as previous studies have demonstrated that this structural characteristic significantly influences antimicrobial activity [31,32,33,34]. Our investigation included both nitrogen-containing heterocyclic cations and phosphorous-containing cations. Nitrogen-containing heterocyclic cations have been extensively studied and well documented, whereas phosphorous-containing cations have received relatively less attention. Nevertheless, phosphonium salts hold great promise as antibacterials, surpassing their nitrogen-containing counterparts due to higher thermal stability and faster synthesis kinetics [35]. Numerous studies propose that the activity of QHSs may be less influenced by counterions compared to the aliphatic chain length or cation type [29,36,37]; however, recent research suggests that specific counterions, particularly those with the ability to oxidize proteins or lipids (e.g., chlorine, bromide, or iron atoms) can enhance QHS activity [16]. Notably, the inclusion of bromide (Br−) in QHS structures has been associated with increased antimicrobial efficacy [16], prompting us to focus this study specifically on the bromide counterion. This approach will enable a deeper understanding of the structural variations that may influence antimicrobial activity in order to establish a structure–activity relationship (SAR) study by doing a rational comparison. To the best of our knowledge, this study represents the first systematic SAR investigation of different types of QHSs using standard antimicrobial susceptibility testing methods.

## 2. Results and Discussion

### 2.1. Chemical Studies 

#### 2.1.1. Synthesis of Quaternary Ammonium and Phosphonium Salts

**Scheme 2:**
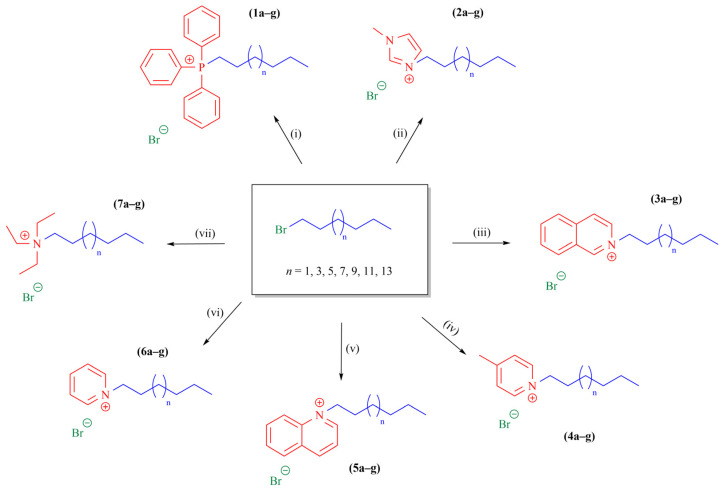
Synthetic strategy followed to obtain quaternary ammonium and phosphonium salts. Reagents and conditions: (i) triphenylphosphine, 130 °C, 24–48 h; (ii) 1-methylimidazole, 130 °C, 24 h; (iii) isoquinoline, 130 °C, 21 h; (iv) 4-picoline, 130 °C, 24 h; (v) quinoline, 130 °C, 21 h; (vi) pyridine, 105 °C, 24 h; (vii) triethylamine, acetonitrile, reflux, 85 °C, 48 h.

The library of 49 structurally related QHSs was designed and synthesised following a straightforward protocol, which is depicted in Scheme 2. The QHSs were obtained by one-pot reactions using commercially available reactants. Hence, the selected halogenated aliphatic chains that have a C_6_ to C_18_ length underwent a nucleophilic substitution reaction with the appropriate amine or phosphine nucleophiles, affording the desired compounds in moderate to high yields. The reactions were carried out under neat conditions at high temperature (130 °C) for approximately 24 h for compounds **1a**–**g**, **2a**–**g**, **3a**–**g**, **4a**–**g**, **5a**–**g**, and **6a**–**g** (Scheme 2(i)–(vi)) while, for compounds **7a**–**g**, heating under reflux with a prolongated reaction time (48 h) was required (Scheme 2(vii)).

All synthesized QHSs have been characterized by NMR (^1^H, ^13^C and DEPT135) spectroscopy (Figures S1–S49, Supplementary Materials).

#### 2.1.2. Determination of the Chromatographic Hydrophobicity Index 

**Figure 2:**
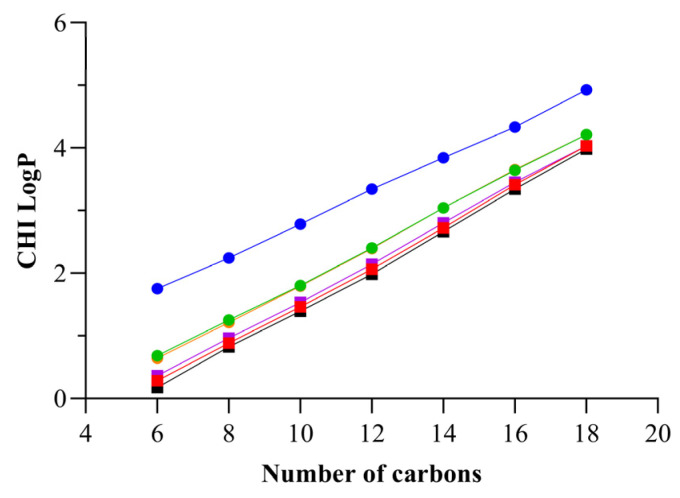
Effect of the number of carbons in the alkyl side-chain in cation on their logarithm of octanol/water partition coefficient (CHI LogP) values. The ● represents compounds **1a**–**g** containing triphenylphosphonium (TPP^+^) cations, ■ represents compounds **2a**–**g** containing methylimidazolium (mim^+^) cations, ● represents compounds **3a**–**g** containing isoquinolinium (IQ^+^) cations, ■ represents compounds **4a**–**g** containing methylpyridinium (mPyr^+^) cations, ● represents compounds **5a**–**g** containing quinolinium (Q^+^) cations, while ■ represents compounds **6a**–**g** containing pyridinium (Pyr^+^) cations.

The chromatographic hydrophobicity index (CHI) values were determined by UHPLC using previously established protocols [38,39,40]. CHI LogP values were then back-calculated from their experimental CHI values (see Equation (1), Section 3.1.3) [40,41]. The CHI values of triethylammonium salts **7a**–**g** could not be determined due to the lack of visible and middle-UV absorption spectra. The corresponding data are presented in Table 1, Figure 2 and Figure S50 (Supplementary Materials). 

Overall, the tested compounds presented CHI LogP values varying significantly from 0.1723 to 4.926 (Table 1). The change in the cationic head group considerably modified the CHI LogP values due to π–π interactions, the number of electronegative atoms present in the cationic ring, and the hydrophobicity of the cationic ring [42]. The presence of the triphenylphosphonium (TPP^+^) cation in compounds **1a**–**g** resulted in the highest relative CHI LogP values (1.747 to 4.926) among all derivatives, indicating that the incorporation of this cation into QHSs increased the compounds’ lipophilicity. It can be observed that compounds **6a**–**g** containing pyridinium (Pyr^+^) cations are characterized by the lowest lipophilicity (CHI LogP values from 0.1723 to 3.982), given the smaller size and less aromatic nature of this aromatic ring. 

From Figure 2, it is clear that CHI LogP values increase with the increment of the size of the alkyl chain attached to the cationic ring. In fact, upon increasing the alkyl chain length of the cationic ring, there is a possibility of strong Van der Waals interactions between the alkyl group of QHS and the hydrophobic C_18_ stationary phase column. This behaviour of hydrophobicity can also be explained by their polarity values: as the size of the alkyl group in QHS increases, its polarity decreases, restricting its ability to interact with water [42].

### 2.2. Microbiological Studies 

#### 2.2.1. Determination of Inhibitory/Bactericidal Activities of Quaternary Ammonium and Phosphonium Salts against an Antibiotic-Susceptible *S. aureus* Strain: Structure–Activity Relationship Analyses

The library of 49 structurally related QHSs was evaluated for their antibacterial activity against the *S. aureus* CECT 976 using the broth microdilution method. The concentrations of the compounds tested in this study ranged from 0.0625 to 64 µg/mL. The results of inhibitory and bactericidal concentrations are presented in Table 2. Considering that lipophilicity is an important property for data interpretation, the experimental CHI LogP values were used for the present discussion. Moreover, for the purpose of facilitating activity comparison, the MIC values of several commonly used antibiotics belonging to various classes, such as ampicillin [AMP] and oxacillin [OXA]—β-lactams, ciprofloxacin [CIP]—fluoroquinolone, erythromycin [ERY]—macrolide, and tetracycline [TET], have been incorporated in the current discussion [43]. The MIC values for [TET] (MIC = 0.96 µg/mL), [CIP] (MIC = 1 µg/mL), and [AMP] (MIC = 1.5 µg/mL) are detailed in Table 2 and fall within a comparable range to that of QHSs with a carbon chain composed of 12–14 atoms (MIC values ranging from 1 to 4 µg/mL). For instance, compounds **1e** and **2e** exhibited MIC values similar to those of [CIP] and [TET]. These findings underscore the promising antibacterial efficacy of these compounds.

From the data shown in Table 2, it is clear that the nature of the cationic head group dramatically influences the QHS’s antibacterial efficiency. Indeed, it is evident that compounds **1a**–**g** containing TPP^+^ and compounds **2a**–**g** containing methylimidazolium (mim^+^) cations exhibited the most promising activity against *S. aureus* CECT 976, as suggested by the MIC and MBC values obtained for the different cationic cores. The higher antibacterial activity of the QPSs based on TPP (compounds **1a**–**g**) could be related to their ability to suppress bacterial bioenergetics by collapsing membrane potential through the activation of protonophorous uncoupling [6,46,47]. The compounds **7a**–**g**, which contain triethylammonium (TEA^+^) cations, elicited the lowest antibacterial activity. This observation is consistent with previous studies [48,49] that highlight the influence of the aromatic nature of the cation on QHS toxicity. Specifically, the presence of an aromatic head group has been associated with increased QHS toxicity, while non-aromatic cations, such as TEA^+^, typically exhibit lower toxicity. The remaining QHSs (containing quinolinium, isoquinolinium, pyridinium, or methylpyridinium cations) lie in between these extremes. Surprisingly, compounds **4a**–**g** and **6a**–**g**, as well as **3a**–**g** and **5a**–**g**, which differ by the nature of the cation, exhibited a similar bactericidal effect under the conditions tested. 

**Figure 3:**
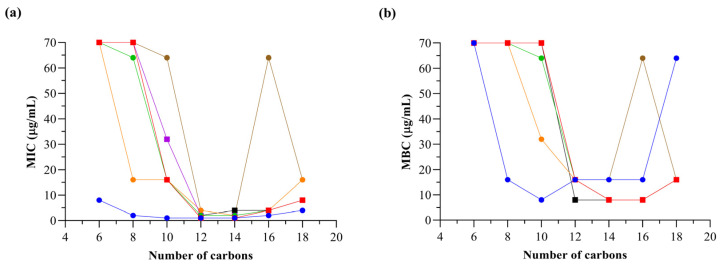
Effect of the number of carbons in the alkyl side-chain in cation on their (**a**) inhibitory and (**b**) bactericidal efficiencies for *S. aureus* CECT 976. The ● represents compounds **1a**–**g** containing triphenylphosphonium (TPP^+^) cations, ■ represents compounds **2a**–**g** containing methylimidazolium (mim^+^) cations, ● represents compounds **3a**–**g** containing isoquinolinium (IQ^+^) cations, ■ represents compounds **4a**–**g** containing methylpyridinium (mPyr^+^) cations, ● represents compounds **5a**–**g** containing quinolinium (Q^+^) cations, ■ represents compounds **6a**–**g** containing pyridinium (Pyr^+^) cations, while ● represents compounds **7a**–**g** containing triethylammonium (TEA^+^) cations.

In this study, it was also highlighted that the length of the alkyl chain is a mandatory structural parameter that ruled the antibacterial activity. Indeed, up to tetradecyl side-chain—C_14_, an increase in chain length leads to a decrease in MIC and MBC values, indicating that the elongation of the alkyl substituent increases the antibacterial activity of the compounds. To illustrate this, the effect of the number of carbons in the alkyl chain of each QHS on their MIC and MBC values for *S. aureus* CECT 976 was graphically compared (Figure 3). Herein, the parabola curve obtained is representative of a square function, and the behaviour attests that C_14_ might correspond to the optimal chain length in this series (MICs = 1–4 μg/mL; MBCs = 8–16 μg/mL; CHI LogP = 2.655–3.836). Furthermore, the replacement of the C_14_ chains with longer or shorter ones has led to drastic changes associated with a clear decrease in activity. The detrimental effect is more obvious in the case of C_6_-homologous, whose antibacterial activity was annihilated for *S. aureus* CECT 976 (MIC, MBC > 64 μg/mL). Compound **1a** is the unique QHS that showed a detectable MIC (but not MBC) for concentrations lower than 64 μg/mL. Previous observations have demonstrated that a higher lipophilicity does not always result in superior antibacterial activity [50]. Actually, the relationship between these two factors follows a non-linear trend that increases as the length of the alkyl chain increases until a threshold value (point) is reached, after which any further extension of the alkyl chain causes a decline in antibacterial activity (also known as the “cut-off” effect) [51,52]. Based on the present data, the reduction in antibacterial activity beyond the C_14_ chain (“cut-off”) might be attributable to several factors, e.g., lower aqueous solubility, micellization, and aggregation due to steric effects in QHSs containing very long alkyl side chains [53]. Additionally, it can be attributed to the mimicry of cell membrane lipids, which results in lower membrane disruption and toxicity [54]. 

The existence of a correlation between efficiency and chain length observed in this study is consistent with data previously published for some QHSs. For instance, the highest efficiency as antibacterial agents was reported for 1-alkylquinolinium and picolinium salts with an alkyl chain length of 14–16 carbon atoms [55,56], 1-alkyl-3-methylimidazolium and 1-alkylpyridinium salts bearing 14 carbon atoms [57,58], 3-alkoxymethyl-1-methylimidazolium salts with 10–14 carbon atoms [59], 1-alkylimidazolium and 1-alkoxymethylimidazolium lactates containing 11 or 12 carbon atoms in the alkyl group [58,59], 1-alkyloxycarbonyloxyethyl-3-methylimidazolium chlorides for 12–14 carbon atoms [60], 1-alkylcarbamylmethyl-3-methylimidazolium bromides and 1-alkylcarbamylmethylpyridinium bromides for 12 carbon atoms in the alkyl chain [58]. However, it must be highlighted that no data have been reported so far for some of the QHSs tested in the present study. To date, the precise bactericidal mechanism of the action of QHSs is unknown, but the main hypothesis-meeting consensus proposes the adsorption of the cationic head group onto the negatively charged bacterial outer layers, followed by the diffusion of the hydrophobic alkyl chains through the lipid bilayer, provoking disruption and loss of membrane integrity. This two-step mechanism assumes an accurate hydrophobic/hydrophilic balance and supports the results reported herein and the “cut-off” effect [4,61,62,63,64].

#### 2.2.2. Determination of Inhibitory/Bactericidal Activities of the Most Promising Quaternary Ammonium and Phosphonium Salts against Antibiotic-Resistant *S. aureus* Strains

In order to provide a more comprehensive investigation into the efficacy of the most potent quaternary salts, namely compounds **1e**, **2e**, **3e**, **4e**, **5e**, **6e**, and **7e**, additional tests against antibiotic-resistant strains were carried out. This study involved the use of the broth microdilution method with three MDR *S. aureus* bacteria, including XU212, SA1199B, and RN4220. The results of the MIC and MBC assays for these strains are presented in Table 3. For *S. aureus* XU212, SA1199B, and RN4220, only [ERY], [TET] and [CIP] antibiotics were used, respectively. Efflux mechanisms play an important role in antibiotic resistance among clinically relevant pathogens, such as *S. aureus*, where NorA, MsrA, and TetK transport proteins are involved [65,66]. Although originally described for the efflux of fluoroquinolones, macrolides, and tetracyclines, these proteins, particularly NorA, actively export a wide range of structurally dissimilar drugs from the bacterial cell. Examples of such drugs include biocides, dyes, quaternary ammonium compounds, and antiseptics [65,66,67]. 

The MIC and MBC values varied significantly depending on the QHS and the specific strain. MIC values ranged from 1 to 2 µg/mL for compound **1e**, 2 to 4 µg/mL for compound **3e**, 2 to 8 µg/mL for compound **5e**, and 4 to 32 µg/mL for compounds **2e**, **4e**, **6e**, and **7e**. Corroborating the findings presented in Section 2.2.1, compound **1e** demonstrated the most promising activity among all the compounds tested. The MBC values were equal to or greater than the corresponding MIC values for all strains tested. The MBC values (µg/mL) ranged from 8 to 64 for compounds **2e** and **6e**, 4 to 8 for compound **3e**, 8 to 32 for compounds **4e** and **7e**, 2 for compound **1e**, and 8 for compound **5e**. Furthermore, substantial differences were observed among the strains, with *S. aureus* XU212 displaying the highest resistance profile, while *S. aureus* RN4220 exhibited the highest susceptibility. Notably, the antibiotic-resistance strains demonstrated higher MIC and MBC values compared to *S. aureus* CECT 976. Considering that *S. aureus* is recognized by the WHO as one of the most clinically significant pathogens associated with uncontrolled infectious diseases, characterized by its high resistance to various antibiotic classes and constant acquisition of additional resistance [68,69], the observed activity of the selected QHSs presents highly promising results. 

#### 2.2.3. Quaternary Ammonium and Phosphonium Salts Activity against Planktonic Bacteria: Dose–Response

Several factors are known to influence the efficacy of antimicrobials in bacterial inactivation, including contact time, the temperature of exposure, the nature of target microorganisms, and antimicrobial concentration [70]. In this study, the relationship between concentration and the bactericidal activity of QHSs against *S. aureus* CECT 976 was evaluated as it is particularly important in assessing the clinical significance of varying microbial tolerances to QHSs. For this purpose, only the most bioactive ones regarding MIC/MBC results (i.e., compounds **1e**, **2e**, **3e**, **4e**, **5e**, **6e**, and **7e**) were chosen.

**Figure 4:**
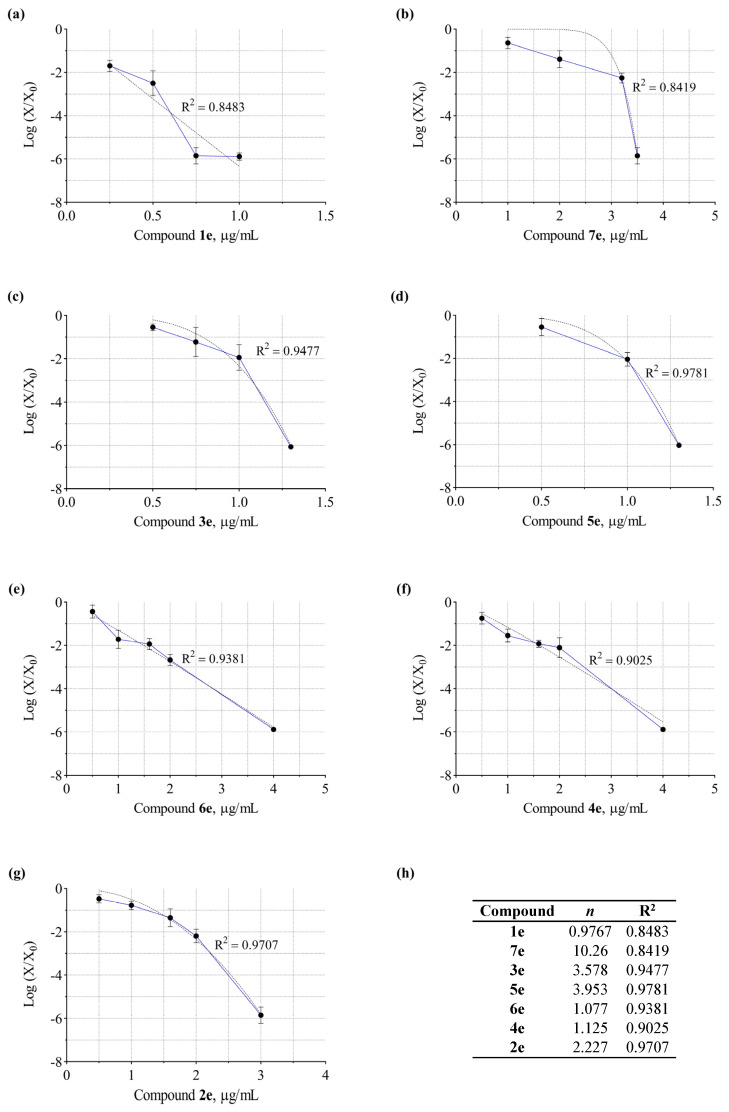
Logarithmic reduction of planktonic *S. aureus* CECT 976 as a function of QHS concentration (µg/mL) after 30 min of exposure to (**a**) compound **1e**, (**b**) compound **7e**, (**c**) compound **3e**, (**d**) compound **5e**, (**e**) compound **6e**, (**f**) compound **4e**, and (**g**) compound **2e**. The blue solid line represents the experimental data and the dashed line the Chick–Watson model fit of experimental data. The points represent the mean ± SD for at least three independent assays. (**h**) Estimated values of the QHS concentration dependence parameter (*n*) considering all experimental points before achieving the maximum log CFU/mL reduction—adapted to the Chick–Watson model. Model adjustability was assessed by the R^2^.

The antibacterial activity of all examined QHSs was found to be dose dependent, following a non-linear dose–response (Figure 4). In general, the antimicrobial effects of the QHSs under study increased with concentration until a maximum biological activity (MBC) was reached; however, the MBC values were lower compared to those reported in the broth microdilution method (see Section 2.2.1). The differences observed in the absolute MBC values could be due to the distinct methods performed for these determinations, as well as differences in medium composition, namely distilled water (DW) and Mueller–Hinton broth (MHB). As it was previously reported that some QHSs can bind to proteins [28], it can be hypothesized that the higher MBC values could be related to the presence of proteins in the MHB medium used for the broth method, which may partly neutralize the antibacterial activity of these QHSs. 

As depicted in Figure 4, *S. aureus* CET 976 was totally eradicated by all quaternary salts under study at a low concentration range (MBC ≤ 4 μg/mL). The long and hydrophobic alkyl side-chains, which may promote strong interactions with bacterial membranes in conjunction with the permeability of the *S. aureus* peptidoglycan cell wall to substances of a high molecular weight, can rationally explain the high susceptibility to these compounds [71]. Particularly, compounds **1e** (MBC = 1 μg/mL; Figure 4a), **5e** and **3e** (both MBC = 1.3 μg/mL; Figure 4c,d) were more effective than the other QHSs in controlling *S. aureus* growth. These MBC values, although different from the ones determined using the broth microdilution method (see Section 2.2.1), matched the same behaviour. Compounds **1e**, **7e**, **5e**, **3e**, **6e**, **4e**, and **2e** at 0.7, 3.4, 1.2, 1.2, 3.2, 3.5, and 2.8 μg/mL, respectively, induced reductions in *S. aureus* log colony-forming units (CFU)/mL > 5. Both [C_14_QBr] and [C_14_IQBr] caused similar maximum log CFU/mL reductions (*p* > 0.05) for *S. aureus* with a 6.0-log reduction at only 1.3 μg/mL (Figure 4c,d). Compounds **6e** and **4e** exhibited similar actions (*p* > 0.05)—maximum reduction of 5.9-log CFU/mL at 4 μg/mL (Figure 4e,f).

The values estimated for the QHS concentration dependence parameter (*n*) from the Chick–Watson model (see Section 3.2.4) are presented in Figure 4h. According to this model, the antimicrobial efficacy of the QHSs with a high *n* value is largely affected by changes in concentration, whereas those with low *n* values are less influenced by QHS concentration and may be more governed by contact time [70,71,72,73]. For *S. aureus*, the estimated *n* values were > 1 for all QHSs (more dose dependent) with the exception of compounds **1e**, **6e**, and **4e**, for which the concentration exponent was circa 1. Hugo and Denyer [74] identified a possible correlation between the *n* value and the sort of interaction between an antimicrobial agent and its cellular target. Antimicrobials originating *n* < 2 include thiol or amino groups, oxidising agents, and membrane-active agents, which bind strongly with their target through chemical or ionic interactions. Thus, the results obtained here suggest that compounds **1e**, **4e**, and **6e** can be membrane-active agents and have strong interactions with their cell targets. By contrast, if *n* > 4, antimicrobials behave as membrane disruptors and proton conductors, such as aliphatic and aromatic alcohols, exhibiting a weak physical interaction with the lipophilic molecules of the bacterial cell envelope. Hence, compound **7e** is the only QHS with possible weakly physical interactions between the cell envelope of *S. aureus*; all the others (compounds **5e**, **3e**, and **2e**) with intermediate *n* values of 2–4 are thought to have a dual action, both chemical and weakly physical. 

### 2.3. Cytotoxicity Cell-Based Studies 

#### Evaluation of the Cytotoxicity Profile in Human Hepatocellular Carcinoma Cells

Apart from antibacterial activity, the assessment of mammalian cell cytotoxicity is crucial in the development of effective antimicrobial agents. The cytotoxicity of QHSs is not only influenced by their structural characteristics but also by the specific cell type under investigation. Bacterial membranes primarily consist of anionic lipids, while mammalian cells predominantly comprise zwitterionic lipids that are neutral at physiological pH. This disparity in membrane electrostatic charges results in targeted tropism of antimicrobial agents toward negatively charged bacteria. Moreover, the presence of cholesterol in mammalian cell membranes provides protection and reduces cytotoxicity upon exposure to microbiocidal components; however, depending on the concentration and structural properties of QHSs, cytotoxicity may still manifest in mammalian cells [1].

In this study, the cytotoxicity profiles of the most potent QHSs, namely compounds **1e**, **2e**, **3e**, **4e**, **5e**, **6e**, and **7e**, were assessed in hepatocarcinoma (HepG2) cells using NR uptake as an endpoint. HepG2 cells are commonly employed in drug metabolism and hepatotoxicity studies, representing a human hepatoma model [75]. These cells exhibit a non-tumorigenic nature, display high proliferation rates, and possess an epithelial-like morphology while retaining many differentiated hepatic functions [76,77]. These characteristics make them a useful model for evaluating the cytotoxic effects of novel compounds, including QHSs.

Initially, the cells were incubated with three different concentrations of each QHS (2, 16, and 32 µg/mL) for 24 h, and the corresponding data are presented in Table 4. Notably, despite the compounds sharing the same alkyl chain length (C_14_) and counterion, the results underscore the substantial influence of different cations on the cytotoxicity observed in HepG2 cells. Moreover, the cytotoxic effect of QHSs was found to be dose dependent. It is interesting to observe that, except for compounds **1e** and **3e**, all QHS compounds displayed cell viability above 85% at the lowest concentration tested (2 µg/mL). Furthermore, both compounds **4e** and **6e** exhibited NR uptake exceeding 90%, further supporting their potential as antimicrobial agents at these concentrations [78]. Comparing the MIC values, it was observed that, except for the previously mentioned compounds, all QHSs maintained cell viability within the therapeutic window range for *S. aureus* CECT 976. Additionally, the MIC value of compound **5e** against *S. aureus* RN4220 fell within the safe concentration range, suggesting its potential as a viable treatment option for this resistant strain. Considering that compounds **1e** and **2e** displayed MIC values of 1 µg/mL for certain strains tested, the cell viability of these compounds was also evaluated at this concentration. The data revealed that only compound **2e** exhibited no detrimental effects on cellular health, as indicated by a 90.88% NR uptake. This highlights the significance of replacing the TPP cation with alternative nitrogen-based carriers to reduce inherent toxicity [79]. 

At the highest QHS concentrations (16 and 32 µg/mL), a significant decrease in cell viability (*p* < 0.0001) to approximately 13% was observed. To address the observed toxicity of QHSs at higher concentrations, a further lead optimization process must be performed to fine-tune their physicochemical, biological, and toxicity properties. Recognizing that the cation is the primary driver of toxicological effects [80], modifying it emerges as a logical approach to achieve more positive outcomes. Potential modifications include introducing substituents into the cationic cores and/or replacing the alkyl side chain with systems containing ether moieties, all while carefully selecting appropriate anions. Moreover, beyond cytotoxicity, various properties of QHSs can be fine-tuned, encompassing solubility, viscosity, melting point, and aggregation [80].

## 3. Materials and Methods

### 3.1. Chemical Studies 

#### 3.1.1. Reagents and Apparatus 

The reagents used as starting materials were purchased from Merck (Darmstadt, Germany), Fluorochem (Hadfield, UK) and Alfa Aesar (Kandel, Germany): 1-bromohexane (CAS 111–25–1, 99.0%), 1-bromooctane (CAS 111–83–1, >98.0%), 1-bromodecane (CAS 112–29–8, 98.0%), 1-bromododecane (CAS 143–15–7, 97.0%), 1-bromotetradecane (CAS 112–71–0, 97.0%), 1-bromohexadecane (CAS 112–82–3, 97.0%), 1-bromooctadecane (CAS 112–89–0, ≥97.0%), triphenylphosphine (CAS 603–35–0, >95.0%), 1-methylimidazole (CAS 616–47–7, ≥99.0%), isoquinoline (CAS 119–65–3, >95.0%), 4-picoline (CAS 108–89–4, 99.0%), triethylamine (CAS 121–44–8, 99.0%), quinoline (CAS 91–22–5, 98.0%), and pyridine (CAS 110–86–1, ≥99.0%). Deuterated chloroform (CDCl_3_) was obtained from Deutero GmbH (Kastellaun, Germany), whereas dichloromethane, methanol, and diethyl ether (all with pro-analysis grade) were acquired from Panreac (Lisbon, Portugal) and Merck (Darmstadt, Germany). For the ultra-high performance liquid chromatography (UHPLC) analysis, the following reagents were used: acetonitrile of HPLC grade (Honeywell Riedel-de Haën—Seelze, Germany), acetic acid glacial of HPLC grade (Carlo Erba—Val de Reuil, France), and ultrapure water from Direct-Pure adept lab water system (RephiLe Bioscience, Ltd—Shanghai, China). All chemicals were used as received without additional purification. 

Syntheses were performed in a reaction station (Radleys Mya 4 station). Analytical thin-layer chromatography (TLC) was performed on pre-coated silica gel 60 F_254_ plates (Merck) with a layer thickness of 0.2 mm. Reaction control was monitored using dichloromethane:methanol (9:1) as the mobile phase, and spots were visualised under UV detection (254 and 366 nm), potassium permanganate (KMnO_4_, 1% aq.) solution, or by exposure to iodine vapor. Flash column chromatography was carried out with silica gel 60 Å (0.040–0.063 mm) (Carlo Erba Reactifs—SDS, Val de Reuil, France) as the stationary phase and dichloromethane:methanol (9:1) as the elution system. Fractions of approximately 15 mL were collected. Automated flash column chromatography was performed on the Biotage Isolera Prime system (Biotage—Uppsala, Sweden) with Biotage SNAP 25 g or 100 g cartridges. The internal detector wavelength employed was 206–366 nm. Solvents were evaporated under reduced pressure in a Büchi Rotavapor.

Nuclear magnetic resonance (NMR) data were acquired on a Bruker Avance III 400 NMR spectrometer, at room temperature, operating at 400.15 MHz for ^1^H, 100.62 MHz for ^13^C, and DEPT135 (Distortionless Enhancement by Polarization Transfer). Chemical shifts are expressed in δ (ppm) values relative to tetramethylsilane (TMS) as the internal reference, and coupling constants (*J*) are given in Hz. DEPT135 values are included in ^13^C NMR data (underlined values).

Chromatograms were acquired on a UHPLC NEXERA SERIES LC-40 equipped with a SCL-40 system controller, FCV-11AL valve unit, SPD-M40 photo diode array detector, DGU-405 degassing unit, and SIL-40C XS autosampler. A C-18 column was used to perform the analysis (Phenomenex Luna—Torrance, USA; 150 mm × 4.6 mm; 5 μm particle size). The mobile phase was acetonitrile/water (gradient mode, room temperature) at a flow rate of 1.0 mL/min, and the injection volume was 40 μL. Data acquisition and processing were carried out with LabSolutions software version 5.93 (Shimadzu, Kyoto, Japan).

#### 3.1.2. Chemical Synthesis and Structural Characterization

##### Synthesis of Triphenylphosphonium, Methylimidazolium, Isoquinolinium, Methylpyridinium, Quinolinium, and Pyridinium Salts

General procedure A. Triphenylphosphine (1.2 eq.), 1-methylimidazole (1.2 eq.), isoquinoline (1.2 eq.), 4-picoline (1.2 eq.), quinoline (1.2 eq.), pyridine (1.2 eq.), and the appropriate bromoalkane (0.5 mL, 1 eq.) were added to a hermetically sealed vessel. The reaction mixture was stirred and heated at 130 °C for 24 h under an argon atmosphere. Thereafter, the crude product was dissolved in dichloromethane and purified by silica gel flash chromatography: compounds **1a**–**g** and **2a**–**g** were purified using dichloromethane:methanol (9:1) as the eluting system, and compounds **3a**–**g**, **4a**–**g**, **5a**–**g**, and **6a**–**g** using gradient elution from dichloromethane to dichloromethane:methanol (8:2). The fractions containing the intended compound were then collected and the solvent evaporated to dryness. The reaction control was performed by TLC (silica gel, dichloromethane:methanol (9:1)). The synthetic procedure was adapted from the literature [81].

In an attempt to reduce the amount of solvent used during the purification steps, another procedure was implemented.

General procedure B. Triphenylphosphine (0.5 g, 1 eq.), 1-methylimidazole (0.250 mL, 1 eq.), isoquinoline (0.250 mL, 1 eq.), 4-picoline (0.250 mL, 1 eq.), quinoline (0.250 mL, 1 eq.), pyridine (0.250 mL, 1 eq.), and the appropriate bromoalkane (1.5 eq.) were added to a hermetically sealed vessel. The reaction mixture was stirred and heated at 130 °C for 24 h under an argon atmosphere. Thereafter, the crude product was dissolved in a minimum amount of dichloromethane and precipitated with ice-cold diethyl ether (for compounds **1a**–**g**, **2a**–**g**, **4a**–**g**, and **6a**–**g**) or ethyl acetate (for compounds **3a**–**g** and **5a**–**g**). The precipitate was filtered and washed with ice-cold diethyl ether or ethyl acetate, respectively. The resulting product was then suspended in dichloromethane and finally obtained through drying under vacuum.

Hexyltriphenylphosphonium bromide (**1a**). Synthesized according to the general procedure A. Pale yellow oil. η = 95%. ^1^H NMR (CDCl_3_): δ = 0.82 (3H, *t*, *J* = 7.1 Hz, H(6)), 1.16–1.33 (4H, *m*, H(4), H(5)), 1.54–1.72 (4H, *m*, H(2), H(3)), 3.69–3.84 (2H, *m*, H(1)), 7.63–8.00 (15H, *m*, H(2′)–H(6′)). ^13^C NMR (CDCl_3_): δ = 14.0 (C(6)), 22.2 (C(5)), 22.6 (*d*, *J*_CP_ = 4.6 Hz, C(3)), 22.8 (*d*, *J*_CP_ = 49.7 Hz, C(1)), 30.1 (*d*, *J*_CP_ = 15.5 Hz, C(2)), 31.3 (*d*, *J*_CP_ = 1.0 Hz, C(4)), 118.4 (*d*, *J*_CP_ = 85.8 Hz, 3 × C(1′)), 130.5 (*d*, *J*_CP_ = 12.5 Hz, 3 × C(3′) and 3 × C(5′)), 133.7 (*d*, *J*_CP_ = 10.0 Hz, 3 × C(2′) and 3 × C(6′)), 135.0 (*d*, *J*_CP_ = 3.0 Hz, 3 × C(4′)).

Octyltriphenylphosphonium bromide (**1b**). Synthesized according to the general procedure A. Pale yellow oil. η = 68%. ^1^H NMR (CDCl_3_): δ = 0.83 (3H, *t*, *J* = 6.9 Hz, H(8)), 1.13–1.32 (8H, *m*, H(4)–H(7)), 1.56–1.70 (4H, *m*, H(2), H(3)), 3.45–3.97 (2H, *m*, H(1)), 7.62–7.94 (15H, *m*, H(2′)–H(6′)). ^13^C NMR (CDCl_3_): δ = 14.1 (C(8)), 22.5 (C(7)), 22.7 (*d*, *J*_CP_ = 4.6 Hz, C(3)), 22.8 (*d*, *J*_CP_ = 49.5 Hz, C(1)), 28.8 (C(6)), 29.2 (*d*, *J*_CP_ = 1.0 Hz, C(4)), 30.4 (*d*, *J*_CP_ = 15.5 Hz, C(2)), 31.7 (C(5)), 118.5 (*d*, *J*_CP_ = 85.8 Hz, 3 × C(1′)), 130.5 (*d*, *J*_CP_ = 12.5 Hz, 3 × C(3′) and 3 × C(5′)), 133.7 (*d*, *J*_CP_ = 10.0 Hz, 3 × C(2′) and 3 × C(6′)), 135.0 (*d*, *J*_CP_ = 3.0 Hz, 3 × C(4′)). 

Decyltriphenylphosphonium bromide (**1c**). Synthesized according to the general procedure A. Pale yellow oil. η = 92%. ^1^H NMR (CDCl_3_): δ = 0.85 (3H, *t*, *J* = 7.0 Hz, H(10)), 1.13–1.32 (12H, *m*, H(4)–H(9)), 1.55–1.69 (4H, *m*, H(2), H(3)), 3.68–3.91 (2H, *m*, H(1)), 7.63–7.96 (15H, *m*, H(2′)–H(6′)). ^13^C NMR (CDCl_3_): δ = 14.1 (C(10)), 22.5 (C(9)), 22.7 (*d*, *J*_CP_ = 4.7 Hz, C(3)), 22.8 (*d*, *J*_CP_ = 49.5 Hz, C(1)), 29.2 (C(7)), 29.2 (C(5), C(6)), 29.5 (C(4)), 30.4 (*d*, *J*_CP_ = 15.5 Hz, C(2)), 31.8 (C(8)), 118.5 (*d*, *J*_CP_ = 85.8 Hz, 3 × C(1′)), 130.5 (*d*, *J*_CP_ = 12.5 Hz, 3 × C(3′) and 3 × C(5′)), 133.7 (*d*, *J*_CP_ = 9.9 Hz, 3 × C(2′) and 3 × C(6′)), 135.0 (*d*, *J*_CP_ = 3.0 Hz, 3 × C(4′)).

Dodecyltriphenylphosphonium bromide (**1d**). Synthesized according to the general procedure A. Pale yellow oil. η = 76%. ^1^H NMR (400 MHz, CDCl_3_): δ = 0.87 (3H, *t*, *J* = 6.9 Hz, H(12)), 1.13–1.33 (16H, *m*, H(4)–H(11)), 1.54–1.69 (4H, *m*, H(2), H(3)), 3.68–3.81 (2H, *m*, H(1)), 7.66–7.90 (15H, *m*, H(2′)–H(6′)). ^13^C NMR (101 MHz, CDCl_3_): δ = 14.1 (C(12)), 22.7 (*d*, *J*_CP_ = 4.4 Hz, C(3)), 22.7 (C(11)), 22.8 (*d*, *J*_CP_ = 49.5 Hz, C(1)), 29.2 (C(9)), 29.2 (C(4)), 29.3 (C(5)), 29.5 (C(6)), 29.6 (C(7), C(8)), 30.4 (*d*, *J*_CP_ = 15.5 Hz, C(2)), 31.9 (C(10)), 118.5 (*d*, *J*_CP_ = 85.8 Hz, 3 × C(1′)), 130.5 (*d*, *J*_CP_ = 12.5 Hz, 3 × C(3′) and 3 × C(5′)), 133.7 (*d*, *J*_CP_ = 9.9 Hz, 3 × C(2′) and 3 × C(6′)), 135.0 (*d*, *J*_CP_ = 3.0 Hz, 3 × C(4′)).

Triphenyl(tetradecyl)phosphonium bromide (**1e**). Synthesized according to the general procedure A. White foam. η = 98%. ^1^H NMR (400 MHz, CDCl_3_): δ = 0.86 (3H, *t*, *J* = 6.9 Hz, H(14)), 1.13–1.34 (20H, *m*, H(4)–H(13)), 1.57–1.67 (4H, *m*, H(2), H(3)), 3.74–3.84 (2H, *m*, H(1)), 7.65–7.91 (15H, *m*, H(2′)–H(6′)). ^13^C NMR (101 MHz, CDCl_3_): δ = 14.1 (C(14)), 22.7 (*d*, *J*_CP_ = 4.4 Hz, C(3)), 22.7 (C(13)), 22.8 (*d*, *J*_CP_ = 49.5 Hz, C(1)), 29.2 (C(11)), 29.3 (C(4)), 29.3 (C(10)), 29.5 (C(5)), 29.6 (C(6)), 29.6 (C(7)), 29.6 (C(8)), 29.7 (C(9)), 30.4 (*d*, *J*_CP_ = 15.5 Hz, C(2)), 31.9 (C(12)), 118.5 (*d*, *J*_CP_ = 85.8 Hz, 3 × C(1′)), 130.5 (*d*, *J*_CP_ = 12.5 Hz, 3 × C(3′) and 3 × C(5′)), 133.7 (*d*, *J*_CP_ = 9.9 Hz, 3 × C(2′) and 3 × C(6′)), 135.0 (*d*, *J*_CP_ = 3.0 Hz, 3 × C(4′)).

Hexadecyltriphenylphosphonium bromide (**1f**). Synthesized according to the general procedure B. White foam. η = 94%. ^1^H NMR (400 MHz, CDCl_3_): δ = 0.87 (3H, *t*, *J* = 6.9 Hz, H(16)), 1.14–1.34 (24H, *m*, H(4)–H(15)), 1.56–1.68 (4H, *m*, H(2), H(3)), 3.74–3.84 (2H, *m*, H(1)), 7.67–7.89 (15H, *m*, H(2′)–H(6′)). ^13^C NMR (101 MHz, CDCl_3_): δ = 14.1 (C(16)), 22.7 (C(15)), 22.7 (*d*, *J*_CP_ = 3.1 Hz, C(3)), 22.9 (*d*, *J*_CP_ = 49.5 Hz, C(1)), 29.2 (C(13)), 29.3 (C(4)), 29.4–29.7 (C(5)–C(12)), 30.4 (*d*, *J*_CP_ = 15.4 Hz, C(2)), 31.9 (C(14)), 118.5 (*d*, *J*_CP_ = 85.8 Hz, 3 × C(1′)), 130.5 (*d*, *J*_CP_ = 12.5 Hz, 3 × C(3′) and 3 × C(5′)), 133.8 (*d*, *J*_CP_ = 10.0 Hz, 3 × C(2′) and 3 × C(6′)), 135.0 (*d*, *J*_CP_ = 3.0 Hz, 3 × C(4′)).

Octadecyltriphenylphosphonium bromide (**1g**). Synthesized according to the general procedure B. White foam. η = 94%. ^1^H NMR (400 MHz, CDCl_3_): δ = 0.87 (3H, *t*, *J* = 7.0 Hz, H(18)), 1.10–1.35 (28H, *m*, H(4)–H(17)), 1.60–1.68 (4H, *m*, H(2), H(3)), 3.74–3.86 (2H, *m*, H(1)), 7.67–7.89 (15H, *m*, H(2′)–H(6′)). ^13^C NMR (101 MHz, CDCl_3_): δ = 14.1 (C(18)), 22.7 (*d*, *J*_CP_ = 3.8 Hz, C(3)), 22.7 (C(17)), 22.8 (*d*, *J*_CP_ = 49.6 Hz, C(1)), 29.2 (C(15)), 29.3 (C(4)), 29.4–29.7 (C(5)–C(14)), 30.4 (*d*, *J*_CP_ = 15.4 Hz, C(2)), 31.9 (C(16)), 118.5 (*d*, *J*_CP_ = 85.8 Hz, 3 × C(1′)), 130.5 (*d*, *J*_CP_ = 12.5 Hz, 3 × C(3′) and 3 × C(5′)), 133.7 (*d*, *J*_CP_ = 10.0 Hz, 3 × C(2′) and 3 × C(6′)), 135.0 (*d*, *J*_CP_ = 3.0 Hz, 3 × C(4′)).

3-Hexyl-1-methyl-1*H*-imidazol-3-ium bromide (**2a**). Synthesized according to the general procedure A. Pale yellow viscous oil. η = 71%. ^1^H NMR (CDCl_3_): δ = 0.87 (3H, *t*, *J* = 7.1 Hz, H(6)), 1.23–1.40 (6H, *m*, H(3)–H(5)), 1.85–1.98 (2H, *m*, H(2)), 4.13 (3H, *s*, H(6′)), 4.33 (2H, *t*, *J* = 7.5 Hz, H(1)), 7.25 (1H, *dd*, *J*_1_ = 1.8 Hz, *J*_2_ = 1.8 Hz, H(4′)), 7.32 (1H, *dd*, *J*_1_ = 1.8 Hz, *J*_2_ = 1.8 Hz, H(5′)), 10.36 (1H, *bs*, H(2′)). ^13^C NMR (CDCl_3_): δ = 13.9 (C(6)), 22.4 (C(5)), 25.9 (C(3)), 30.2 (C(2)), 31.1 (C(4)), 36.8 (C(6′)), 50.3 (C(1)), 121.5 (C(5′)), 123.0 (C(4′)), 138.3 (C(2′)). 

1-Methyl-3-octyl-1*H*-imidazol-3-ium bromide (**2b**). Synthesized according to the general procedure A. Pale yellow viscous oil. η = 88%. ^1^H NMR (CDCl_3_): δ = 0.84 (3H, *t*, *J* = 6.9 Hz, H(8)), 1.15–1.40 (10H, *m*, H(3)–H(7)), 1.84–1.94 (2H, *m*, H(2)), 4.11 (3H, *s*, H(6′)), 4.33 (2H, *t*, *J* = 7.4 Hz, H(1)), 7.36 (1H, *dd*, *J*_1_ = 1.8 Hz, *J*_2_ = 1.8 Hz, H(4′)), 7.50 (1H, *dd*, *J*_1_ = 1.8 Hz, *J*_2_ = 1.8 Hz, H(5′)), 10.40 (1H, *bs*, H(2′)). ^13^C NMR (CDCl_3_): δ = 14.1 (C(8)), 22.6 (C(7)), 26.3 (C(2)), 28.9 (C(3)), 29.0 (C(5)), 30.3 (C(4)), 31.7 (C(6)), 36.8 (C(6′)), 50.2 (C(1)), 121.8 (C(5′)), 123.5 (C(4′)), 137.7 (C(2′)).

3-Decyl-1-methyl-1*H*-imidazol-3-ium bromide (**2c**). Synthesized according to the general procedure A. Pale yellow foam. η = 94%. ^1^H NMR (CDCl_3_): δ = 0.87 (3H, *t*, *J* = 6.9 Hz, H(10)), 1.17–1.41 (14H, *m*, H(3)–H(9)), 1.84–2.03 (2H, *m*, H(2)), 4.13 (3H, *s*, H(6′)), 4.32 (2H, *t*, *J* = 7.4 Hz, H(1)), 7.35 (1H, *dd*, *J*_1_ = 1.8 Hz, *J*_2_ = 1.8 Hz, H(4′)), 7.49 (1H, *dd*, *J*_1_ = 1.8 Hz, *J*_2_ = 1.8 Hz, H(5′)), 10.42 (1H, *bs*, H(2′)). ^13^C NMR (CDCl_3_): δ = 14.1 (C(10)), 22.7 (C(9)), 26.3 (C(2)), 29.0 (C(7)), 29.2 (C(6)), 29.4 (C(5)), 29.5 (C(4)), 30.3 (C(8)), 31.8 (C(3)), 36.8 (C(6′)), 50.2 (C(1)), 121.7 (C(5′)), 123.5 (C(4′)), 137.8 (C(2′)).

3-Dodecyl-1-methyl-1*H*-imidazol-3-ium bromide (**2d**). Synthesized according to the general procedure A. Pale yellow foam. η = 90%. ^1^H NMR (400 MHz, CDCl_3_): δ = 0.87 (3H, *t*, *J* = 6.9 Hz, H(12)), 1.20–1.40 (18H, *m*, H(3)–H(11)), 1.87–1.96 (2H, *m*, H(2)), 4.14 (3H, *s*, H(6′)), 4.32 (2H, *t*, *J* = 7.4 Hz, H(1)), 7.38 (1H, *dd*, *J*_1_ = 1.8 Hz, *J*_2_ = 1.8 Hz, H(4′)), 7.53 (1H, *dd*, *J*_1_ = 1.7 Hz, *J*_2_ = 1.7 Hz, H(5′)), 10.36 (1H, *bs*, H(2′)). ^13^C NMR (101 MHz, CDCl_3_): δ = 14.1 (C(12)), 22.7 (C(11)), 26.3 (C(3)), 29.0 (C(4)), 29.3 (C(8)), 29.4 (C(7)), 29.5 (C(5), C(6)), 29.6 (C(3)), 30.3 (C(2)), 31.9 (C(10)), 36.8 (C(6′)), 50.2 (C(1)), 121.8 (C(5′)), 123.5 (C(4′)), 137.7 (C(2′)).

1-Methyl-3-tetradecyl-1*H*-imidazol-3-ium bromide (**2e**). Synthesized according to the general procedure B. Pale yellow foam. η = 92%. ^1^H NMR (400 MHz, CDCl_3_): δ = 0.88 (3H, *t*, *J* = 6.9 Hz, H(14)), 1.19–1.40 (22H, *m*, H(3)–H(13)), 1.83–1.99 (2H, *m*, H(2)), 4.14 (3H, *s*, H(6′)), 4.32 (2H, *t*, *J* = 7.4 Hz, H(1)), 7.32 (1H, *dd*, *J*_1_ = 1.8 Hz, *J*_2_ = 1.8 Hz, H(4′)), 7.44 (1H, *dd*, *J*_1_ = 1.8 Hz, *J*_2_ = 1.8 Hz, H(5′)), 10.49 (1H, *bs*, H(2′)). ^13^C NMR (101 MHz, CDCl_3_): δ = 14.1 (C(14)), 22.7 (C(13)), 26.3 (C(3)), 29.0 (C(4)), 29.4 (C(10)), 29.4 (C(9)), 29.5 (C(8)), 29.6 (C(5)), 29.6 (C(6), C(7)), 29.7 (C(11)), 30.3 (C(2)), 31.9 (C(12)), 36.8 (C(6′)), 50.2 (C(1)), 121.7 (C(5′)), 123.5 (C(4′)), 137.8 (C(2′)).

3-Hexadecyl-1-methyl-1*H*-imidazol-3-ium bromide (**2f**). Synthesized according to the general procedure B. Pale yellow foam. η = 89%. ^1^H NMR (400 MHz, CDCl_3_): δ = 0.87 (3H, *t*, *J* = 6.9 Hz, H(16)), 1.18–1.40 (26H, *m*, H(3)–H(15)), 1.85–1.98 (2H, *m*, H(2)), 4.14 (3H, *s*, H(6′)), 4.32 (2H, *t*, *J* = 7.4 Hz, H(1)), 7.32 (1H, *dd*, *J*_1_ = 1.8 Hz, *J*_2_ = 1.8 Hz, H(4′)), 7.44 (1H, *dd*, *J*_1_ = 1.8 Hz, *J*_2_ = 1.8 Hz, H(5′)), 10.52 (1H, *bs*, H(2′)). ^13^C NMR (101 MHz, CDCl_3_): δ = 14.1 (C(16)), 22.7 (C(15)), 26.3 (C(3)), 29.0 (C(4)), 29.4 (C(13)), 29.4–29.7 (C(5)–C(12)), 30.3 (C(2)), 31.9 (C(14), 36.8 (C(6′)), 50.3 (C(1)), 121.7 (C(5′)), 123.4 (C(4′)), 137.9 (C(2′)).

1-Methyl-3-octadecyl-1*H*-imidazol-3-ium bromide (**2g**). Synthesized according to the general procedure B. Pale yellow foam. η = 96%. ^1^H NMR (400 MHz, CDCl_3_): δ = 0.87 (3H, *t*, *J* = 6.9 Hz, H(18)), 1.19–1.39 (30H, *m*, H(3)–H(17)), 1.87–1.97 (2H, *m*, H(2)), 4.14 (3H, *s*, H(6′)), 4.32 (2H, *t*, *J* = 7.4 Hz, H(1)), 7.30 (1H, *dd*, *J*_1_ = 1.8 Hz, *J*_2_ = 1.8 Hz, H(4′)), 7.41 (1H, *dd*, *J*_1_ = 1.8 Hz, *J*_2_ = 1.8 Hz, H(5′)), 10.55 (1H, *bs*, H(2′)). ^13^C NMR (101 MHz, CDCl_3_): δ = 14.1 (C(18)), 22.7 (C(17)), 26.3 (C(3)), 29.0 (C(4)), 29.4 (C(15)), 29.4–29.7 (C(5)–C(14)), 30.3 (C(2)), 31.9 (C(16), 36.8 (C(6′)), 50.2 (C(1)), 121.7 (C(5′)), 123.4 (C(4′)), 137.8 (C(2′)).

2-Hexylisoquinolin-2-ium bromide (**3a**). Synthesized according to the general procedure A. Brownish solid. η = 78%. ^1^H NMR (CDCl_3_): 0.83 (3H, *t*, *J* = 7.1 Hz, H(6)), 1.18–1.50 (6H, *m*, H(3)–H(5)), 2.06–2.20 (2H, *m*, H(2)), 5.09 (2H, *t*, *J* = 7.4 Hz, H(1)), 7.95 (1H, *ddd*, *J* = 8.2, 6.7, 1.4 Hz, H(7′)), 8.08–8.20 (2H, *m*, H(5′), H(6′)), 8.39 (1H, *d*, *J* = 6.8 Hz, H(4′)), 8.79 (2H, *m*, H(3′), H(8′)), 11.12 (1H, *dd*, H(1′)). ^13^C NMR (CDCl_3_): δ = 14.1 (C(6)), 22.3 (C(5)), 25.9 (C(3)), 31.2 (C(2)), 31.9 (C(4)), 61.7 (C(1)), 126.3 (C(8′)), 127.1 (C(5′)), 127.9 (C(8a’)), 131.3 (C(6′)), 131.4 (C(7′)), 134.4 (C(4′)), 137.1 (C(3′)), 137.3 (C(4a’)), 150.5 (C(1′)).

2-Octylisoquinolin-2-ium bromide (**3b**). Synthesized according to the general procedure A. Brownish solid. η = 85%. ^1^H NMR (CDCl_3_): δ = 0.84 (3H, *t*, *J* = 6.9 Hz, H(8)), 1.13–1.49 (10H, *m*, H(3)–H(7)), 2.05–2.19 (2H, *m*, H(2)), 5.09 (2H, *t*, *J* = 7.4 Hz, H(1)), 7.95 (1H, *ddd*, *J* = 8.2, 6.5, 1.6 Hz, H(7′)), 8.07–8.20 (2H, *m*, H(5′), H(6′)), 8.37 (1H, *d*, *J* = 6.8 Hz, H(4′)), 8.77 (2H, *m*, H(3′), H(8′)), 11.16 (1H, *dd*, H(1′)). ^13^C NMR (CDCl_3_): δ = 14.1 (C(8)), 22.6 (C(7)), 26.2 (C(3)), 29.0 (C(5)), 29.1 (C(4)), 31.7 (C(2)), 32.0 (C(6)), 61.7 (C(1)), 126.3 (C(8′)), 127.1 (C(5′)), 127.9 (C(8a’)), 131.4 (C(6′)), 131.5 (C(7′)), 134.3 (C(4′)), 137.1 (C(3′)), 137.3 (C(4a’)), 150.6 (C(1′)).

2-Decylisoquinolin-2-ium bromide (**3c**). Synthesized according to the general procedure A. Brownish solid. η = 45%. ^1^H NMR (CDCl_3_): δ = 0.85 (3H, *t*, *J* = 6.9 Hz, H(10)), 1.15–1.49 (14H, *m*, H(3)–H(9)), 2.06–2.21 (2H, *m*, H(2)), 5.09 (2H, *t*, *J* = 7.4 Hz, H(1)), 7.95 (1H, *ddd*, *J* = 8.2, 6.7, 1.4 Hz, H(7′)), 8.08–8.20 (2H, *m*, H(5′), H(6′)), 8.40 (1H, *d*, *J* = 6.8 Hz, H(4′)), 8.74–8.84 (2H, *m*, H(3′), H(8′)), 11.10 (1H, *dd*, H(1′)). ^13^C NMR (CDCl_3_): δ = 14.1 (C(10)), 22.7 (C(9)), 26.2 (C(3)), 29.1 (C(7)), 29.2 (C(4)), 29.3 (C(6)), 29.4 (C(5)), 31.8 (C(2)), 31.9 (C(8)), 61.7 (C(1)), 126.2 (C(8′)), 127.0 (C(5′)), 128.0 (C(8a’)), 131.4 (C(6′)), 131.5 (C(7′)), 134.2 (C(4′)), 137.1 (C(3′)), 137.3 (C(4a’)), 150.8 (C(1′)).

2-Dodecylisoquinolin-2-ium bromide (**3d**). Synthesized according to the general procedure A. Brownish solid. η = 72%. ^1^H NMR (400 MHz, CDCl_3_): δ = 0.86 (3H, *t*, *J* = 6.9 Hz, H(12)), 1.16–1.46 (18H, *m*, H(3)–H(11)), 2.05–2.18 (2H, *m*, H(2)), 5.08 (2H, *t*, *J* = 7.4 Hz, H(1)), 7.96 (1H, *ddd*, *J* = 8.2, 6.7, 1.4 Hz, H(7′)), 8.09–8.16 (2H, *m*, H(5′), H(6′)), 8.32 (1H, *d*, *J* = 6.8 Hz, H(4′)), 8.64 (1H, *dd*, *J* = 6.8, 1.5 Hz, H(8′)), 8.79 (1H, *dd*, *J* = 8.3, 1.0 Hz, H(3′)), 11.14 (1H, *s*, H(1′)). ^13^C NMR (101 MHz, CDCl_3_): δ = 14.1 (C(12)), 22.6 (C(11)), 26.2 (C(3)), 29.1 (C(4)), 29.3 (C(6)), 29.3 (C(8)), 29.5 (C(7)), 29.6 (C(5), C(9)), 31.9 (C(2)), 31.9 (C(10)), 61.7 (C(1)), 126.2 (C(8′)), 127.0 (C(5′)), 127.9 (C(8a’)), 131.3 (C(6′)), 131.4 (C(7′)), 134.3 (C(4′)), 137.1 (C(3′)), 137.3 (C(4a’)), 150.5 (C(1′)).

2-Tetradecylisoquinolin-2-ium bromide (**3e**). Synthesized according to the general procedure A. Brownish solid. η = 86%. ^1^H NMR (400 MHz, CDCl_3_): δ = 0.86 (3H, *t*, *J* = 6.9 Hz, H(14)), 1.14–1.47 (22H, *m*, H(3)–H(13)), 2.05–2.19 (2H, *m*, H(2)), 5.08 (2H, *t*, *J* = 7.4 Hz, H(1)), 7.95 (1H, *ddd*, *J* = 8.2, 6.5, 1.7 Hz, H(7′)), 8.09–8.16 (2H, *m*, H(5′), H(6′)), 8.37 (1H, *d*, *J* = 6.8 Hz, H(4′)), 8.73 (1H, *d*, *J* = 6.8 Hz, H(8′)), 8.78 (1H, *dd*, *J* = 8.4, 1.0 Hz, H(3′)), 11.09 (1H, *s*, H(1′)). ^13^C NMR (101 MHz, CDCl_3_): δ = 14.1 (C(14)), 22.7 (C(13)), 26.2 (C(3)), 29.1 (C(4)), 29.3 (C(13)), 29.4 (C(10)), 29.5 (C(9)), 29.6 (C(5)), 29.6 (C(6)), 29.6 (C(7)), 29.7 (C(8)), 31.9 (C(2)), 31.9 (C(12)), 61.7 (C(1)), 126.2 (C(8′)), 127.0 (C(5′)), 127.9 (C(8a’)), 131.4 (C(6′)), 131.5 (C(7′)), 134.2 (C(4′)), 137.1 (C(3′)), 137.3 (C(4a’)), 150.6 (C(1′)).

2-Hexadecylisoquinolin-2-ium bromide (**3f**). Synthesized according to the general procedure B. Brownish solid. η = 81%. ^1^H NMR (400 MHz, CDCl_3_): δ = 0.87 (3H, *t*, *J* = 6.9 Hz, H(16)), 1.15–1.46 (26H, *m*, H(3)–H(15)), 2.06–2.18 (2H, *m*, H(2)), 5.09 (2H, *t*, *J* = 7.4 Hz, H(1)), 7.96 (1H, *ddd*, *J* = 8.2, 6.7, 1.4 Hz, H(7′)), 8.09–8.16 (2H, *m*, H(5′), H(6′)), 8.32 (1H, *d*, *J* = 6.8 Hz, H(4′)), 8.65 (1H, *dd*, *J* = 6.8, 1.4 Hz, H(8′)), 8.78 (1H, *dd*, *J* = 8.3, 1.0, H(3′)), 11.16 (1H, *s*, H(1′)). ^13^C NMR (101 MHz, CDCl_3_): δ = 14.1 (C(16)), 22.7 (C(15)), 26.2 (C(3)), 29.1 (C(4)), 29.4 (C(13)), 29.5–29.7 (C(5)–C(12)), 31.9 (C(2), C(14)), 61.7 (C(1)), 126.1 (C(8′)), 127.0 (C(5′)), 127.9 (C(8a’)), 131.4 (C(6′)), 131.5 (C(7′)), 134.2 (C(4′)), 137.1 (C(3′)), 137.2 (C(4a’)), 150.7 (C(1′)).

2-Octyldecylisoquinolin-2-ium bromide (**3g**). Synthesized according to the general procedure B. Brownish solid. η = 98%. ^1^H NMR (400 MHz, CDCl_3_): δ = 0.87 (3H, *t*, *J* = 6.9 Hz, H(18)), 1.15–1.46 (30H, *m*, H(3)–H(17)), 2.07–2.18 (2H, *m*, H(2)), 5.09 (2H, *t*, *J* = 7.4 Hz, H(1)), 7.95 (1H, *ddd*, *J* = 8.2, 6.3, 1.8 Hz, H(7′)), 8.09–8.17 (2H, *m*, H(5′), H(6′)), 8.36 (1H, *d*, *J* = 6.8 Hz, H(4′)), 8.72 (1H, *dd*, *J* = 6.8, 1.5 Hz, H(8′)), 8.78 (1H, *dd*, *J* = 8.4, 1.1 Hz, H(3′)), 11.12 (1H, *s*, H(1′)). ^13^C NMR (101 MHz, CDCl_3_): δ = 14.1 (C(18)), 22.7 (C(17)), 26.2 (C(3)), 29.1 (C(4)), 29.4 (C(15)), 29.5–29.9 (C(5)–C(14)), 31.9 (C(2), C(16)), 61.7 (C(1)), 126.1 (C(8′)), 127.0 (C(5′)), 127.9 (C(8a’)), 131.4 (C(6′)), 131.5 (C(7′)), 134.1 (C(4′)), 137.1 (C(3′)), 137.2 (C(4a’)), 150.7 (C(1′)).

1-Hexyl-4-methylpyridin-1-ium bromide (**4a**). Synthesized according to the general procedure A. Red oil. η = 93%. ^1^H NMR (CDCl_3_): δ = 0.86 (3H, *t*, *J* = 7.1 Hz, H(6)), 1.19–1.45 (6H, *m*, H(3)–H(5)), 1.94–2.12 (2H, *m*, H(2)), 2.68 (3H, *s*, H(7′)), 4.90 (2H, *t*, *J* = 7.4 Hz, H(1)), 7.92 (2H, *d*, *J* = 6.7 Hz, H(3′), H(5′)), 9.36 (2H, *d*, *J* = 6.7 Hz, H(2′), H(6′)). ^13^C NMR (CDCl_3_): δ = 14.0 (C(6)), 22.3 (C(7′)), 22.5 (C(5)), 25.8 (C(3)), 31.2 (C(2)), 31.9 (C(4)), 61.4 (C(1)), 129.0 (C(3′), C(5′)), 144.4 (C(2′), C(6′)), 158.9 (C(4′)).

4-Methyl-1-octylpyridin-1-ium bromide (**4b**). Synthesized according to the general procedure A. Red oil. η = 78%. ^1^H NMR (CDCl_3_): δ = 0.86 (3H, *t*, *J* = 6.9 Hz, H(8)), 1.15–1.44 (10H, *m*, H(3)–H(7)), 1.94–2.08 (2H, *m*, H(2)), 2.68 (3H, *s*, H(7′)), 4.91 (2H, *t*, *J* = 7.4 Hz, H(1)), 7.91 (2H, *d*, *J* = 6.7 Hz, H(3′), H(5′)), 9.34 (2H, *d*, *J* = 6.7 Hz, H(2′), H(6′)). ^13^C NMR (CDCl_3_): δ = 14.1 (C(8)), 22.3 (C(7′)), 22.6 (C(7)), 26.1 (C(3)), 29.0 (C(5), C(4)), 31.7 (C(2)), 31.9 (C(6)), 61.4 (C(1)), 128.9 (C(3′), C(5′)), 144.4 (C(2′), C(6′)), 158.9 (C(4′)). 

1-Decyl-4-methylpyridin-1-ium bromide (**4c**). Synthesized according to the general procedure A. Red oil. η = 98%. ^1^H NMR (CDCl_3_): δ = 0.87 (3H, *t*, *J* = 6.9 Hz, H(10)), 1.12–1.45 (14H, *m*, H(3)–H(9)), 1.94–2.10 (2H, *m*, H(2)), 2.68 (3H, *s*, H(7′)), 4.91 (2H, *t*, *J* = 7.4 Hz, H(1)), 7.90 (2H, *d*, *J* = 6.7 Hz, H(3′), H(5′)), 9.31 (2H, *d*, *J* = 6.7 Hz, H(2′), H(6′)). ^13^C NMR (CDCl_3_): δ = 14.1 (C(10)), 22.3 (C(7′)), 22.7 (C(9)), 26.1 (C(3)), 29.1 (C(7)), 29.3 (C(6)), 29.4 (C(5)), 29.5 (C(4)), 31.8 (C(2)), 31.9 (C(8)), 61.4 (C(1)), 128.9 (C(3′), C(5′)), 144.4 (C(2′), C(6′)), 158.9 (C(4′)).

1-Dodecyl-4-methylpyridin-1-ium bromide (**4d**). Synthesized according to the general procedure A. Brownish foam. η = 74%. ^1^H NMR (400 MHz, CDCl_3_): δ = 0.87 (3H, *t*, *J* = 6.8 Hz, H(12)), 1.16–1.42 (18H, *m*, H(3)–H(11)), 1.96–2.07 (2H, *m*, H(2)), 2.68 (3H, *s*, H(7′)), 4.90 (2H, *t*, *J* = 7.4 Hz, H(1)), 7.91 (2H, *d*, *J* = 6.3 Hz, H(3′), H(5′)), 9.31 (2H, *d*, *J* = 6.6 Hz, H(2′), H(6′)). ^13^C NMR (101 MHz, CDCl_3_): δ = 14.1 (C(12)), 22.3 (C(7′)), 22.7 (C(11)), 26.1 (C(3)), 29.1 (C(4)), 29.3 (C(9)), 29.4 (C(5)), 29.5 (C(6)), 29.6 (C(7), C(8)), 31.9 (C(2)), 31.9 (C(10)), 61.3 (C(1)), 128.9 (C(3′), C(5′)), 144.3 (C(2′), C(6′)), 158.8 (C(4′)).

4-Methyl-1-tetradecylpyridin-1-ium bromide (**4e**). Synthesized according to the general procedure A. Brownish foam. η = 79%. ^1^H NMR (400 MHz, CDCl_3_): δ = 0.87 (3H, *t*, *J* = 6.8 Hz, H(14)), 1.16–1.41 (22H, *m*, H(3)–H(13)), 1.96–2.06 (2H, *m*, H(2)), 2.67 (3H, *s*, H(7′)), 4.90 (2H, *t*, *J* = 7.4 Hz, H(1)), 7.90 (2H, *d*, *J* = 6.3 Hz, H(3′), H(5′)), 9.29 (2H, *d*, *J* = 6.7 Hz, H(2′), H(6′)). ^13^C NMR (101 MHz, CDCl_3_): δ = 14.1 (C(14)), 22.3 (C(7′)), 22.7 (C(13)), 26.1 (C(3)), 29.1 (C(4)), 29.3 (C(11)), 29.4 (C(9)), 29.5 (C(8)), 29.6 (C(5)), 29.6 (C(6), C(7)), 29.7 (C(10)), 31.8 (C(2)), 31.9 (C(12)), 61.3 (C(1)), 128.8 (C(3′), C(5′)), 144.2 (C(2′), C(6′)), 158.8 (C(4′)).

1-Hexadecyl-4-methylpyridin-1-ium bromide (**4f**). Synthesized according to the general procedure B. Brownish foam. η = 97%. ^1^H NMR (400 MHz, CDCl_3_): δ = 0.87 (3H, *t*, *J* = 6.8 Hz, H(16)), 1.19–1.41 (26H, *m*, H(3)–H(15)), 1.94–2.06 (2H, *m*, H(2)), 2.68 (3H, *s*, H(7′)), 4.91 (2H, *t*, *J* = 7.4 Hz, H(1)), 7.87 (2H, *d*, *J* = 6.3 Hz, H(3′), H(5′)), 9.26 (2H, *d*, *J* = 6.7 Hz, H(2′), H(6′)). ^13^C NMR (101 MHz, CDCl_3_): δ = 14.1 (C(16)), 22.3 (C(7′)), 22.7 (C(15)), 26.1 (C(3)), 29.1 (C(4)), 29.4 (C(13)), 29.5–29.7 (C(5)–C(12)), 31.9 (C(2)), 31.9 (C(14)), 61.4 (C(1)), 128.8 (C(3′), C(5′)), 144.2 (C(2′), C(6′)), 158.8 (C(4′)).

4-Methyl-1-octadecylpyridin-1-ium bromide (**4g**). Synthesized according to the general procedure B. Brownish foam. η = 90%. ^1^H NMR (400 MHz, CDCl_3_): δ = 0.87 (3H, *t*, *J* = 6.8 Hz, H(18)), 1.17–1.40 (30H, *m*, H(3)–H(17)), 1.95–2.05 (2H, *m*, H(2)), 2.67 (3H, *s*, H(7′)), 4.92 (2H, *t*, *J* = 7.4 Hz, H(1)), 7.89 (2H, *d*, *J* = 6.2 Hz, H(3′), H(5′)), 9.31 (2H, *d*, *J* = 6.7 Hz, H(2′), H(6′)). ^13^C NMR (101 MHz, CDCl_3_): δ = 14.1 (C(18)), 22.3 (C(7′)), 22.7 (C(17)), 26.1 (C(3)), 29.1 (C(4)), 29.3 (C(15)), 29.4–29.7 (C(5)–C(14)), 31.8 (C(2)), 31.9 (C(16)), 61.3 (C(1)), 128.8 (C(3′), C(5′)), 144.2 (C(2′), C(6′)), 158.8 (C(4′)).

1-Hexylquinolin-1-ium bromide (**5a**). Synthesized according to the general procedure A. Dark-red solid. η = 78%. ^1^H NMR (CDCl_3_): 0.87 (3H, *t*, *J* = 7.1 Hz, H(6)), 1.24–1.41 (4H, *m*, H(4), H(5)), 1.45–1.61 (2H, *m*, H(3)), 2.05–2.19 (2H, *m*, H(2)), 5.42 (2H, *t*, *J* = 7.6 Hz, H(1)), 7.98 (1H, *ddd*, *J* = 8.0, 7.0, 1.0 Hz, H(6′)), 8.19–8.25 (2H, *m*, H(3′), H(7′)), 8.31–8.38 (2H, *m*, H(5′), H(8′)), 9.06 (1H, *d*, *J* = 8.3 Hz, H(2′)), 10.64 (1H, *dd*, *J* = 5.9, 1.4 Hz, H(4′)). ^13^C NMR (CDCl_3_): δ = 13.9 (C(6)), 22.4 (C(5)), 26.2 (C(3)), 30.4 (C(2)), 31.2 (C(4)), 58.2 (C(1)), 118.4 (C(8′)), 122.7 (C(3′)), 130.0 (C(4a’)), 130.2 (C(6′)), 131.2 (C(5′)), 136.0 (C(7′)), 137.7 (C(8a’)), 147.3 (C(2′)), 150.6 (C(4′)). 

1-Octylquinolin-1-ium bromide (**5b**). Synthesized according to the general procedure A. Dark-red solid. η = 85%. ^1^H NMR (CDCl_3_): δ = 0.84 (3H, *t*, *J* = 7.1 Hz, H(8)), 1.16–1.40 (8H, *m*, H(4)–H(7)), 1.46–1.58 (2H, *m*, H(3)), 2.05–2.16 (2H, *m*, H(2)), 5.43 (2H, *t*, *J* = 7.6 Hz, H(1)), 7.97 (1H, *ddd*, *J* = 8.0, 7.0, 0.9 Hz, H(6′)), 8.18–8.26 (2H, *m*, H(3′), H(7′)), 8.33–8.40 (2H, *m*, H(5′), H(8′)), 9.10 (1H, *d*, *J* = 8.3 Hz, H(2′)), 10.61 (1H, *dd*, *J* = 5.8, 1.4 Hz, H(4′)). ^13^C NMR (CDCl_3_): δ = 14.0 (C(8)), 22.6 (C(7)), 26.5 (C(3)), 29.0 (C(5)), 29.1 (C(4)), 30.5 (C(2)), 31.7 (C(6)), 58.2 (C(1)), 118.3 (C(8′)), 122.8 (C(3′)), 130.0 (C(4a’)), 130.1 (C(6′)), 131.1 (C(5′)), 135.9 (C(7′)), 137.7 (C(8a’)), 146.8 (C(2′)), 151.0 (C(4′)). 

1-Decylquinolin-1-ium bromide (**5c**). Synthesized according to the general procedure A. Dark-red solid. η = 45%. ^1^H NMR (CDCl_3_): δ = 0.85 (3H, *t*, *J* = 7.1 Hz, H(10)), 1.15–1.40 (12H, *m*, H(4)–H(9)), 1.45–1.57 (2H, *m*, H(3)), 2.06–2.16 (2H, *m*, H(2)), 5.43 (2H, *t*, *J* = 7.6 Hz, H(1)), 7.97 (1H, *ddd*, *J* = 8.0, 7.0, 0.9 Hz, H(6′)), 8.18–8.25 (2H, *m*, H(3′), H(7′)), 8.34–8.40 (2H, *m*, H(5′), H(8′)), 9.11 (1H, *d*, *J* = 8.4 Hz, H(2′)), 10.61 (1H, *dd*, *J* = 5.9, 1.4 Hz, H(4′)). ^13^C NMR (CDCl_3_): δ = 14.1 (C(10)), 22.6 (C(9)), 26.5 (C(3)), 29.2 (C(7), (C(4)), 29.4 (C(6)), 29.5 (C(5)), 30.5 (C(2)), 31.8 (C(8)), 58.2 (C(1)), 118.3 (C(8′)), 122.7 (C(3′)), 130.0 (C(4a’)), 130.1 (C(6′)), 131.2 (C(5′)), 135.9 (C(7′)), 137.7 (C(8a’)), 147.1 (C(2′)), 150.8 (C(4′)).

1-Dodecylquinolin-1-ium bromide (**5d**). Synthesized according to the general procedure A. Brownish solid. η = 82%. ^1^H NMR (400 MHz, CDCl_3_): δ = 0.86 (3H, *t*, *J* = 7.1 Hz, H(12)), 1.17–1.40 (16H, *m*, H(4)–H(11)), 1.45–1.61 (2H, *m*, H(3)), 2.06–2.15 (2H, *m*, H(2)), 5.42 (2H, *t*, *J* = 7.7 Hz, H(1)), 7.98 (1H, *ddd*, *J* = 8.0, 7.0, 0.9 Hz, H(6′)), 8.19–8.26 (2H, *m*, H(3′), H(7′)), 8.36–8.42 (2H, *m*, H(5′), H(8′)), 9.15 (1H, *d*, *J* = 8.4 Hz, H(2′)), 10.54 (1H, *dd*, *J* = 5.8, 1.4 Hz, H(4′)). ^13^C NMR (101 MHz, CDCl_3_): δ = 14.1 (C(12)), 22.7 (C(11)), 26.5 (C(3)), 29.2 (C(4)), 29.3 (C(9)), 29.3 (C(8)), 29.5 (C(7)), 29.6 (C(5), C(6)), 30.4 (C(2)), 31.9 (C(10)), 58.2 (C(1)), 118.3 (C(8′)), 122.7 (C(3′)), 130.0 (C(4a’)), 130.1 (C(6′)), 131.1 (C(5′)), 135.9 (C(7′)), 137.7 (C(8a’)), 147.1 (C(2′)), 150.7 (C(4′)).

1-Tetradecylquinolin-1-ium bromide (**5e**). Synthesized according to the general procedure A. Brownish solid. η = 83%. ^1^H NMR (400 MHz, CDCl_3_): δ = 0.86 (3H, *t*, *J* = 7.1 Hz, H(14)), 1.17–1.40 (20H, *m*, H(4)–H(13)), 1.45–1.62 (2H, *m*, H(3)), 2.06–2.17 (2H, *m*, H(2)), 5.43 (2H, *t*, *J* = 7.6 Hz, H(1)), 7.97 (1H, *ddd*, *J* = 8.3, 6.9, 0.9 Hz, H(6′)), 8.18–8.26 (2H, *m*, H(3′), H(7′)), 8.34–8.43 (2H, *m*, H(5′), H(8′)), 9.13 (1H, *d*, *J* = 8.2 Hz, H(2′)), 10.57 (1H, *dd*, *J* = 5.5, 1.4 Hz, H(4′)). ^13^C NMR (101 MHz, CDCl_3_): δ = 14.1 (C(14)), 22.7 (C(13)), 26.5 (C(3)), 29.2 (C(4)), 29.3 (C(11)), 29.4 (C(10)), 29.5 (C(9)), 29.6 (C(7), 29.6 (C(8)), 29.6 (C(6)), 29.7 (C(5)), 30.5 (C(2)), 31.9 (C(12)), 58.2 (C(1)), 118.3 (C(8′)), 122.8 (C(3′)), 130.0 (C(4a’)), 130.1 (C(6′)), 131.1 (C(5′)), 135.9 (C(7′)), 137.7 (C(8a’)), 147.1 (C(2′)), 150.8 (C(4′)).

1-Hexadecylquinolin-1-ium bromide (**5f**). Synthesized according to the general procedure B. Brownish solid. η = 68%. ^1^H NMR (400 MHz, CDCl_3_): δ = 0.87 (3H, *t*, *J* = 7.1 Hz, H(16)), 1.17–1.40 (24H, *m*, H(4)–H(15)), 1.46–1.57 (2H, *m*, H(3)), 2.06–2.15 (2H, *m*, H(2)), 5.43 (2H, *t*, *J* = 7.6 Hz, H(1)), 7.97 (1H, *ddd*, *J* = 8.0, 7.0, 0.9 Hz, H(6′)), 8.19–8.25 (2H, *m*, H(3′), H(7′)), 8.35–8.41 (2H, *m*, H(5′), H(8′)), 9.13 (1H, *d*, *J* = 8.4 Hz, H(2′)), 10.57 (1H, *dd*, *J* = 5.9, 1.4 Hz, H(4′)). ^13^C NMR (101 MHz, CDCl_3_): δ = 14.1 (C(16)), 22.7 (C(15)), 26.5 (C(3)), 29.2 (C(4)), 29.3 (C(13)), 29.4–29.7 (C(5)–C(12)), 30.5 (C(2)), 31.9 (C(14)), 58.2 (C(1)), 118.3 (C(8′)), 122.7 (C(3′)), 130.0 (C(4a’)), 130.1 (C(6′)), 131.1 (C(5′)), 135.9 (C(7′)), 137.7 (C(8a’)), 147.0 (C(2′)), 150.8 (C(4′)).

1-Octadecylquinolin-1-ium bromide (**5g**). Synthesized according to the general procedure B. Brownish solid. η = 65%. ^1^H NMR (400 MHz, CDCl_3_): δ = 0.88 (3H, *t*, *J* = 6.8 Hz, H(18)), 1.16–1.41 (28H, *m*, H(4)–H(17)), 1.46–1.58 (2H, *m*, H(3)), 2.06–2.16 (2H, *m*, H(2)), 5.42 (2H, *t*, *J* = 7.6 Hz, H(1)), 7.97 (1H, *ddd*, *J* = 8.0, 7.0, 0.9 Hz, H(6′)), 8.18–8.25 (2H, *m*, H(3′), H(7′)), 8.33–8.40 (2H, *m*, H(5′), H(8′)), 9.10 (1H, *d*, *J* = 8.3 Hz, H(2′)), 10.59 (1H, *dd*, *J* = 5.7, 1.4 Hz, H(4′)). ^13^C NMR (101 MHz, CDCl_3_): δ = 14.1 (C(18)), 22.7 (C(17)), 26.5 (C(3)), 29.2 (C(4)), 29.3 (C(15)), 29.4–29.7 (C(5)–C(14)), 30.5 (C(2)), 31.9 (C(16)), 58.2 (C(1)), 118.3 (C(8′)), 122.7 (C(3′)), 130.0 (C(4a’)), 130.1 (C(6′)), 131.1 (C(5′)), 135.9 (C(7′)), 137.7 (C(8a’)), 147.0 (C(2′)), 150.8 (C(4′)).

1-Hexylpyridin-1-ium bromide (**6a**). Synthesized according to the general procedure A. Pale yellow viscous oil. η = 98%. ^1^H NMR (CDCl_3_): δ = 0.87 (3H, *t*, *J* = 7.1 Hz, H(6)), 1.20–1.46 (6H, *m*, H(3)–H(5)), 2.00–2.12 (2H, *m*, H(2)), 5.01 (2H, *t*, *J* = 7.5 Hz, H(1)), 8.18 (2H, *dd*, *J* = 7.8, 6.5 Hz, H(3′), H(5′)), 8.56 (1H, *tt*, *J* = 7.8, 1.4 Hz, H(4′)), 9.56 (2H, *dd*, *J* = 6.5, 1.4 Hz, H(2′), H(6′)). ^13^C NMR (CDCl_3_): δ = 13.9 (C(6)), 22.4 (C(5)), 25.7 (C(3)), 31.1 (C(2)), 32.0 (C(4)), 62.1 (C(1)), 128.5 (C(3′), C(5′)), 145.2 (C(2′), C(4′) and C(6′)). 

1-Octylpyridin-1-ium bromide (**6b**). Synthesized according to the general procedure A. Pale yellow viscous oil. η = 99%. ^1^H NMR (CDCl_3_): δ = 0.85 (3H, *t*, *J* = 7.1 Hz, H(8)), 1.15–1.46 (10H, *m*, H(3)–H(7)), 1.93–2.13 (2H, *m*, H(2)), 5.02 (2H, *t*, *J* = 7.5 Hz, H(1)), 8.16 (2H, *dd*, *J* = 7.8, 6.5 Hz, H(3′), H(5′)), 8.54 (1H, *tt*, *J* = 7.8, 1.4 Hz, H(4′)), 9.52 (2H, *dd*, *J* = 6.5, 1.4 Hz, H(2′), H(6′)). ^13^C NMR (CDCl_3_): δ = 14.1 (C(8)), 22.6 (C(7)), 26.1 (C(3)), 29.0 (C(5), C(4)), 31.7 (C(2)), 32.0 (C(6)), 62.2 (C(1)), 128.5 (C(3′), C(5′)), 145.1 (C(2′), C(4′) and C(6′)).

1-Decylpyridin-1-ium bromide (**6c**). Synthesized according to the general procedure A. Pale yellow viscous oil. η = 99%. ^1^H NMR (CDCl_3_): δ = 0.86 (3H, *t*, *J* = 7.1 Hz, H(10)), 1.17–1.44 (14H, *m*, H(3)–H(9)), 1.96–2.09 (2H, *m*, H(2)), 5.01 (2H, *t*, *J* = 7.5 Hz, H(1)), 8.17 (2H, *dd*, *J* = 7.8, 6.7 Hz, H(3′), H(5′)), 8.54 (1H, *tt*, *J* = 7.8, 1.4 Hz, H(4′)), 9.51 (2H, *dd*, *J* = 6.7, 1.4 Hz, H(2′), H(6′)). ^13^C NMR (CDCl_3_): δ = 14.1 (C(10)), 22.7 (C(9)), 26.1 (C(3)), 29.1 (C(7)), 29.2 (C(6)), 29.3 (C(5)), 29.4 (C(4)), 31.8 (C(2)), 32.0 (C(8)), 62.2 (C(1)), 128.4 (C(3′), C(5′)), 145.1 (C(2′), C(4′) and C(6′)).

1-Dodecylpyridin-1-ium bromide (**6d**). Synthesized according to the general procedure B. White foam. η = 98%. ^1^H NMR (400 MHz, CDCl_3_): δ = 0.87 (3H, *t*, *J* = 6.8 Hz, H(12)), 1.18–1.44 (18H, *m*, H(3)–H(11)), 1.99–2.14 (2H, *m*, H(2)), 5.01 (2H, *t*, *J* = 7.4 Hz, H(1)), 8.17 (2H, *dd*, *J* = 7.7, 6.3 Hz, H(3′), H(5′)), 8.54 (1H, *dddd*, *J* = 7.7, 7.7, 1.4, 1.4 Hz, H(4′)), 9.53 (2H, *dd*, *J* = 6.3, 1.4 Hz, H(2′), H(6′)). ^13^C NMR (101 MHz, CDCl_3_): δ = 14.1 (C(12)), 22.6 (C(11)), 26.1 (C(3)), 29.1 (C(4)), 29.3 (C(9)), 29.3 (C(7)), 29.5 (C(6)), 29.6 (C(5), C(8)), 31.9 (C(2)), 32.0 (C(10)), 62.1 (C(1)), 128.5 (C(3′), C(5′)), 145.1 (C(4′)), 145.2 ((C(2′) and C(6′)).

1-Tetradecylpyridin-1-ium bromide (**6e**). Synthesized according to the general procedure B. White foam. η = 98%. ^1^H NMR (400 MHz, CDCl_3_): δ = 0.87 (3H, *t*, *J* = 6.8 Hz, H(14)), 1.18–1.43 (22H, *m*, H(3)–H(13)), 2.00–2.10 (2H, *m*, H(2)), 5.02 (2H, *t*, *J* = 7.5 Hz, H(1)), 8.15 (2H, *dd*, *J* = 7.8, 6.7 Hz, H(3′), H(5′)), 8.53 (1H, *dddd*, *J* = 7.8, 7.8, 1.3, 1.3 Hz, H(4′)), 9.50 (2H, *dd*, *J* = 6.7, 1.3 Hz, H(2′), H(6′)). ^13^C NMR (101 MHz, CDCl_3_): δ = 14.1 (C(14)), 22.7 (C(13)), 26.1 (C(3)), 29.1 (C(4), 29.4 (C(10)), 29.5 (C(9)), 29.6 (C(8)), 29.6 (C(5), C(6)), 29.7 (C(7), C(11)), 31.9 (C(2)), 32.0 (C(12)), 62.2 (C(1)), 128.4 (C(3′), C(5′)), 145.1 (C(4′)), 145.2 (C(2′) and C(6′)).

1-Hexadecylpyridin-1-ium bromide (**6f**). Synthesized according to the general procedure B. White foam. η = 97%. ^1^H NMR (400 MHz, CDCl_3_): δ = 0.87 (3H, *t*, *J* = 6.8 Hz, H(16)), 1.19–1.43 (26H, *m*, H(3)–H(15)), 1.99–2.10 (2H, *m*, H(2)), 5.02 (2H, *t*, *J* = 7.4 Hz, H(1)), 8.15 (2H, *dd*, *J* = 7.8, 6.7 Hz, H(3′), H(5′)), 8.53 (1H, *tt*, *J* = 7.8, 1.4 Hz, H(4′)), 9.50 (2H, *dd*, *J* = 6.7, 1.4 Hz, H(2′), H(6′)). ^13^C NMR (101 MHz, CDCl_3_): δ = 14.1 (C(16)), 22.7 (C(15)), 26.1 (C(3)), 29.1 (C(4)), 29.4 (C(13)), 29.5–29.7 (C(5)–C(12)), 31.9 (C(2)), 32.0 (C(14)), 62.2 (C(1)), 128.4 (C(3′), C(5′)), 145.1 (C(4′)), 145.2 (C(2′) and C(6′)).

1-Octadecylpyridin-1-ium bromide (**6g**). Synthesized according to the general procedure B. White foam. η = 98%. ^1^H NMR (400 MHz, CDCl_3_): δ = 0.87 (3H, *t*, *J* = 6.8 Hz, H(18)), 1.18–1.43 (30H, *m*, H(3)–H(17)), 1.99–2.09 (2H, *m*, H(2)), 5.02 (2H, *t*, *J* = 7.4 Hz, H(1)), 8.15 (2H, *dd*, *J* = 7.8, 6.7 Hz, H(3′), H(5′)), 8.53 (1H, *tt*, *J* = 7.8, 1.4 Hz, H(4′)), 9.50 (2H, *dd*, *J* = 6.7, 1.4 Hz, H(2′), H(6′)). ^13^C NMR (101 MHz, CDCl_3_): δ = 14.1 (C(18)), 22.7 (C(17)), 26.1 (C(3)), 29.1 (C(4)), 29.4 (C(15)), 29.5–29.7 (C(5)–C(14)), 31.9 (C(2)), 32.0 (C(16)), 62.2 (C(1)), 128.4 (C(3′), C(5′)), 145.1 (C(4′)), 145.2 (C(2′) and C(6′)).

##### Synthesis of Triethylammonium Salts

Triethylamine (1.2 eq.), 4 mL of acetonitrile, and the appropriate bromoalkane (0.5 mL, 1 eq.) were added to a hermetically sealed vessel. The reaction mixture was stirred and heated at 85 °C for 48 h. Acetonitrile was evaporated, and the crude product was poured into water (15 mL) and extracted with diethyl ether (2 × 5 mL). The aqueous phase was concentrated, and the solvent was evaporated under reduced pressure. The synthetic procedure was adapted from the literature [82].

*N*,*N*,*N*-Triethylhexan-1-aminium bromide (**7a**). White foam. η = 77%.^1^H NMR (CDCl_3_): δ = 0.90 (3H, *t*, *J* = 7.1 Hz, H(6)), 1.26–1.49 (6H, *m*, H(3)–H(5)), 1.40 (9H, *t*, *J* = 7.3 Hz, 3 × H(2′)), 1.63–1.77 (2H, *m*, H(2)), 3.19–3.39 (2H, *m*, H(1)), 3.53 (6H, *q*, *J* = 7.3 Hz, 3 × H(1′)). ^13^C NMR (CDCl_3_): δ = 8.2 (3 × C(2′)), 14.0 (C(6)), 22.2 (C(5)), 22.4 (C(3)), 26.2 (C(2)), 31.2 (C(4)), 53.7 (3 × C(1′)), 57.7 (C(1)). 

*N*,*N*,*N*-Triethyloctan-1-aminium bromide (**7b**). White foam. η = 84%.^1^H NMR (CDCl_3_): δ = 0.88 (3H, *t*, *J* = 6.9 Hz, H(8)), 1.17–1.49 (10H, *m*, H(3)–H(7)), 1.40 (9H, *t*, *J* = 7.3 Hz, 3 × H(2′)), 1.62–1.78 (2H, *m*, H(2)), 3.25–3.30 (2H, *m*, H(1)), 3.52 (6H, *q*, *J* = 7.3 Hz, 3 × H(1′)). ^13^C NMR (CDCl_3_): δ = 8.2 (3 × C(2′)), 14.0 (C(8)), 22.1 (C(7)), 22.6 (C(2)), 26.5 (C(3)), 29.1 (C(5)), 29.2 (C(4)), 31.8 (C(6)), 53.7 (3 × C(1′)), 57.7 (C(1)). 

*N*,*N*,*N*-Triethyldecan-1-aminium bromide (**7c**). White foam. η = 81%.^1^H NMR (CDCl_3_): δ = 0.88 (3H, *t*, *J* = 6.9 Hz, H(10)), 1.18–1.50 (14H, *m*, H(3)–H(9)), 1.40 (9H, *t*, *J* = 7.3 Hz, 3 × H(2′)), 1.63–1.77 (2H, *m*, H(2)), 3.10–3.32 (2H, *m*, H(1)), 3.53 (6H, *m*, *J* = 7.3 Hz, 3 × H(1′)). ^13^C NMR (CDCl_3_): δ = 8.2 (3 × C(2′)), 14.1 (C(10)), 22.1 (C(9)), 22.7 (C(2)), 26.5 (C(3)), 29.2 (C(7), C(4)), 29.4 (C(5)), 31.8 (C(8)), 53.6 (3 × C(1′)), 57.6 (C(1)).

*N*,*N*,*N*-Triethyldodecan-1-aminium bromide (**7d**). White foam. η = 62%. ^1^H NMR (400 MHz, CDCl_3_): δ = 0.87 (3H, *t*, *J* = 6.9 Hz, H(12)), 1.21–1.48 (18H, *m*, H(3)–H(11)), 1.41 (9H, *t*, *J* = 7.3 Hz, 3 × H(2′)), 1.64–1.76 (2H, *m*, H(2)), 3.13–3.31 (2H, *m*, H(1)), 3.52 (6H, *q*, *J* = 7.3 Hz, 3 × H(1′)). ^13^C NMR (101 MHz, CDCl_3_): δ = 8.2 (3 × C(2′)), 14.1 (C(12)), 22.1 (C(11)), 22.7 (C(2)), 26.5 (C(3)), 29.2 (C(4)), 29.3 (C(9)), 29.4 (C(7)), 29.5 (C(8)), 29.6 (C(5), C(6)), 31.9 (C(10)), 53.7 (3 × C(1′)), 57.6 (C(1)).

*N*,*N*,*N*-Triethyltetradecan-1-aminium bromide (**7e**). White foam. η = 42%. ^1^H NMR (400 MHz, CDCl_3_): δ = 0.88 (3H, *t*, *J* = 6.9 Hz, H(14)), 1.21–1.48 (22H, *m*, H(3)–H(13)), 1.40 (9H, *t*, *J* = 7.3 Hz, 3 × H(2′)), 1.64–1.76 (2H, *m*, H(2)), 3.11–3.32 (2H, *m*, H(1)), 3.52 (6H, *q*, *J* = 7.3 Hz, 3 × H(1′)). ^13^C NMR (101 MHz, CDCl_3_): δ = 8.2 (3 × C(2′)), 14.1 (C(14)), 22.1 (C(13)), 22.7 (C(2)), 26.5 (C(3)), 29.2 (C(4)), 29.4 (C(11)), 29.4 (C(9)), 29.5 (C(10)), 29.6 (C(7)), 29.6 (C(8)), 29.7 (C(5), C(6)), 31.9 (C(12)), 53.6 (3 × C(1′)), 57.6 (C(1)).

*N*,*N*,*N*-Triethylhexadecan-1-aminium bromide (**7f**). White foam. η = 55%. ^1^H NMR (400 MHz, CDCl_3_): δ = 0.88 (3H, *t*, *J* = 6.9 Hz, H(16)), 1.19–1.46 (26H, *m*, H(3)–H(15)), 1.40 (9H, *t*, *J* = 7.3 Hz, 3 × H(2′)), 1.63–1.75 (2H, *m*, H(2)), 3.20–3.31 (2H, *m*, H(1)), 3.52 (6H, *q*, *J* = 7.3 Hz, 3 × H(1′)). ^13^C NMR (101 MHz, CDCl_3_): δ = 8.2 (3 × C(2′)), 14.1 (C(16)), 22.1 (C(15)), 22.7 (C(2)), 26.5 (C(3)), 29.2 (C(4)), 29.4 (C(13)), 29.4–29.7 (C(5)–C(12)), 31.9 (C(14)), 53.7 (3 × C(1′)), 57.6 (C(1)).

*N*,*N*,*N*-Triethyloctadecan-1-aminium bromide (**7g**). White foam. η = 91%. ^1^H NMR (400 MHz, CDCl_3_): δ = 0.87 (3H, *t*, *J* = 6.9 Hz, H(18)), 1.20–1.46 (30H, *m*, H(3)–H(17)), 1.40 (9H, *t*, *J* = 7.3 Hz, 3 × H(2′)), 1.64–1.76 (2H, *m*, H(2)), 3.18–3.32 (2H, *m*, H(1)), 3.52 (6H, *q*, *J* = 7.3 Hz, 3 × H(1′)). ^13^C NMR (101 MHz, CDCl_3_): δ = 8.2 (3 × C(2′)), 14.1 (C(18)), 22.1 (C(17)), 22.7 (C(2)), 26.5 (C(3)), 29.2 (C(4)), 29.4 (C(15)), 29.4–29.7 (C(5)–C(14)), 31.9 (C(16)), 53.7 (3 × C(1′)), 57.6 (C(1)).

#### 3.1.3. Evaluation of the Chromatographic Hydrophobicity Index

The chromatographic hydrophobicity index (CHI) values were determined using the retention times (t_R_) of the samples under study and of a mixture of standard compounds on a UHPLC NEXERA SERIES LC-40 with a Luna C18 (2) column of 150 × 4.6 mm, 5 μm (Phenomenex, Torrance, CA, USA). Stock solutions of the test compounds (10 mM) were prepared in dimethyl sulfoxide (DMSO, 99.9%; Carlo Erba) and diluted in acetonitrile/water (1:1) to obtain a final concentration of 500 μM. The mobile phase A was 1.0% (*v*/*v*) aqueous acetic acid solution (pH = 2.6), and the mobile phase B was acetonitrile. The following gradient programme was applied: 1–7 min 0–100% B, 7–17 min 100% B, 17–19 min 100–0% B, 1 mL/min flow; rt, injection volume 40 μL.

A calibration line was obtained using a mixture of the following compounds: benzimidazole, theophylline, paracetamol, caffeine, colchicine, carbamazepine, indole, propiophenone, butyrophenone, valerophenone, and heptanophenone (Table S1 and Figure S50, Supplementary Materials). The experimental CHI values of the compounds tested at pH 2.6 were then converted to the logarithm of octanol/water partition coefficient (CHI LogP) values using Equation (1) [40,41].

CHI LogP = 0.047 × CHI + 0.36 × HBC − 1.10 (1)

where HBC is the hydrogen bond donor count.

### 3.2. Microbiological Studies 

#### 3.2.1. Preparation of the Quaternary Ammonium and Phosphonium Salts

Stock solutions of triethylammonium salts were prepared in sterile distilled water (DW) under aseptic conditions. For the other compounds, dimethyl sulfoxide (DMSO; VWR, Fontenay-sous-Bois, France) was used as the solvent. The percentage of DMSO in the final solutions never exceeded 10% (*v*/*v*).

#### 3.2.2. Bacterial Strains and Culture Conditions

*S. aureus* CECT 976, obtained from the Spanish Type Culture Collection, was used as the standard susceptibility testing control strain. This strain has been previously employed in diverse studies to validate the antimicrobial effects of novel molecules [45,83]. Additional antibiotic-resistant strains, *S. aureus* XU212, SA1199B, and RN4220, generously provided by S. Gibbons (University College London, London, UK) were tested. The RN4220 strain exhibits resistance to 14- and 15-membered macrolides, including erythromycin, and harbours multi-copies of plasmid pUL5054, which carries the gene encoding the MsrA macrolide efflux protein. The SA1199B strain is characterized by its resistance to fluoroquinolones due to the overexpression of the NorA efflux pump and its ciprofloxacin resistance. The XU212 strain possesses the TetK efflux pump and is additionally an MSRA strain [83]. 

All bacterial strains were cryopreserved at −80 °C in a mixture of Mueller–Hinton broth (MHB; Merck) and 30% (*v*/*v*) glycerol. Prior to testing, the strains were sub-cultured on Plate Count Agar (PCA; VWR, Leuven, Belgium) and incubated at 37 °C for 24 h. Single colonies were then inoculated in 50 mL MHB and grown at 37 °C with agitation (150 rpm; INCU-Line IL 150R) until the logarithmic (exponential growth) phase was reached (≈16–18 h). These cultures were used for each experiment. 

#### 3.2.3. Evaluation of Minimum Inhibitory Concentration and Minimum Bactericidal Concentration

The minimum inhibitory concentration (MIC) and minimum bactericidal concentration (MBC) values of all QHSs were determined by the broth microdilution method, according to the approved M7-A7 (aerobes) standards of the Clinical and Laboratory Standards Institute (CLSI) guidelines [45]. Overnight cell cultures of *S. aureus*, in the exponential phase of growth, were adjusted to an optical density (OD) of 0.132 ± 0.02 (λ = 600 nm). Then, a volume of 180 µL of the cell suspension was added to sterile, flat, clear-bottomed polystyrene (PS) 96-well microtiter plates (VWR—Radnor, PA, USA), already containing 20 µL of each QHS under study at concentrations ranging from 0.0625 to 64 µg/mL. In the end, the volume of each compound never exceeded 10% (*v*/*v*) of the well volume. Microtiter plates were then incubated for 24 h at 37 °C under agitation (150 rpm; INCU-Line IL 150R incubator). Absorbance measurements were performed at the beginning (*t* = 0 h) and end (*t* = 24 h) of the incubation period using a microtiter plate absorbance reader (BMG LABTECH FLUOstar Omega). Bacterial suspensions with DMSO and without QHSs were used as negative controls. The MIC was defined as the lowest concentration of the compound at which no bacterial growth was detected (i.e., the final OD was equal to or lower than the initial OD). All tests were performed with six replicates and a minimum of three independent repeats. 

After MIC determination, a volume of 10 µL of each concentration tested for MIC assessment was plated out on PCA. Plates were incubated at 37 °C for 24 h, and growth was visually inspected. The MBC was determined as the lowest concentration of compound in which the total inhibition of growth on solid medium was observed (no colony forming units (CFUs) were detected). All tests were performed in triplicate, and with a minimum of three independent repeats. 

#### 3.2.4. Antimicrobial Susceptibility Test: Dose–Response

A quantitative suspension test to explore the dose–response relationships of QHSs by evaluating bactericidal activity was performed [84]. For this method, overnight grown cultures were centrifuged (VWR Mega Star 600R) at 3772× *g* for 10 min and washed twice with phosphate-buffered saline (PBS; 1.44 g/L of sodium thiosulfate—Labkem, Barcelona, Spain; 8 g/L of sodium chloride, 0.2 g/L of potassium chloride, and 0.24 g/L of potassium dihydrogen phosphate—VWR, Leuven, Belgium) at pH 7.4. The collected cells were resuspended in PBS and adjusted to an optical density (OD) of 0.132 ± 0.02 (λ = 600 nm). Afterwards, 100 µL of this cell suspension was aseptically transferred to 800 µL of DW and allowed to stand for 2 min. To this mixture, 100 µL of each QHS solution diluted in DMSO at the desired concentration was added. The dose–response evaluation took place for 30 min at 37 °C. In the case of the negative control (untreated cells), the QHS solution was replaced by an equal volume of DMSO or DW. At the end of the contact time, QHS neutralization was performed through the dilution-neutralization method [84] using a universal neutralizer (1 g/L L-histidine (Merck—Tokyo, Japan), 3 g/L lecithin (Alfa Aesar—Karlsruhe, Germany), 5 g/L sodium thiosulphate (Labkem—Barcelona, Spain), 30 g/L saponin (VWR Chemicals—Leuven, Belgium), and 30 g/L polysorbate 80 (VWR Chemicals—Le Havre, France) in 0.0025 M phosphate buffer). For neutralization, the test blend (100 µL) was added to 800 µL sterile aqueous neutralizing solution and 100 µL of DW. The mixture was gently agitated and allowed to stand for 10 min, after which serial dilutions (up to 10^−5^) in sterile saline solution (0.85% *w*/*v* NaCl) were prepared. Then, 10 µL of each serial dilution was plated into Petri dishes containing PCA by the drop-plating method. The plates were allowed to set, then inverted and placed in an incubator at 37 °C for 24 h. The surviving cells on the QHS neutralized samples were determined by CFU counting on each plate, ensuring the counting of dilution plates that gave between 10 and 100 CFUs. The antibacterial activity was evaluated by quantification of logarithmic reduction (log CFU/mL), according to Equation (2).

log CFU/mL reduction = log (X) − log (X_0_), (2)

where X_0_ and X are the counts of CFU/mL before (untreated control cells) and after quaternary salt exposure, respectively.

The QHS concentrations chosen for conducting the dose–response tests comprise of the MBC, which causes the complete reduction of planktonic cells, as well as lower concentrations. Specifically, compound **1e** was evaluated at concentrations of 0.25, 0.5, 0.75, 1, 2, 4, 8, and 16 µg/mL, compound **7e** at concentrations of 1, 2, 3.2, 3.5, 4, 8, and 16 µg/mL, compound **5e** at concentrations of 0.5, 1, 1.3, 1.6, 2, 4, and 8 µg/mL, and compound **3e** at concentrations of 0.5, 0.75, 1, 1.3, 1.6, 2, 4, and 8 µg/mL. In the case of compounds **2e**, **6e**, and **4e**, concentrations tested were 0.5, 1, 1.6, 2, 4, and 8 µg/mL. All tests were performed in duplicate, and with a minimum of three independent repeats.

The universal neutralizer was validated in terms of neutralizing efficacy against the QHSs tested and for the absence of antibacterial activity.Effective QHS neutralization and no detectable neutralizer activity were observed for *S. aureus* exposed to QHSs. 

The Chick–Watson mathematical model, provided in Equation (3), is considered an adequate approach to validate the kinetics of dose–response data [85].

log X/X_0_ = − *K*_dis_ C*^n^ t* (3)

where X_0_ and X are the counts of CFU/mL before and after quaternary salt exposure, respectively; *K*_dis_ is a killing rate coefficient ((mL.µg^−1^)*^n^*.min^−1^); C is the quaternary salt concentration (µg.mL^−1^); *n* is the concentration exponent and *t* is the exposure time (min). The concentration dependence of killing (*n*) was determined using a toolbox for linear regression in GraphPad Prism (version 8.4.0, GraphPad Software, La Jolla, CA, USA). The quality of the model was evaluated through the regression coefficient (*R*^2^).

### 3.3. Cytotoxicity Cell-Based Studies 

#### 3.3.1. Reagents and Solvents

Neutral red (NR) solution, trypan blue solution [0.4% (*w*/*v*)], and high-glucose Dulbecco’s modified Eagle’s medium (DMEM, D7777) were acquired from Sigma (St. Louis, MO, USA). The reagents used in the cell cultures, including heat-inactivated fetal bovine serum (FBS), non-essential amino acids (NEAA), 0.25% trypsin/1 mM EDTA, antibiotics (10,000 U/mL penicillin and 10,000 µg/mL streptomycin), PBS, and Hank’s balanced salt solution without calcium and magnesium [HBSS (−/−)] were purchased from Gibco Laboratories (Lenexa, KS, USA). DMSO, methanol, absolute ethanol, and acetic acid were obtained from Merck (Darmstadt, Germany). 

#### 3.3.2. Preparation of the Quaternary Ammonium and Phosphonium Salts 

Stock solutions of the test QHSs (32 µg/mL) were prepared under aseptic conditions in DMSO and stored at −20 °C. Test dilutions were freshly prepared in complete cell culture medium immediately before use. The percentage of DMSO in the final solutions never exceeded 0.1% (*v*/*v*) of the total volume. 

#### 3.3.3. Cell Culture and Conditions

The evaluation of the cytotoxicity effects of QHSs was performed using hepatocellular carcinoma (HepG2) cells. HepG2 cells (ATCC^®^, HB-8065^TM^) were routinely cultured in 75 cm^2^ flasks using DMEM with 25 mM glucose, supplemented with 44 mM sodium bicarbonate, 10% FBS, 100 U/mL penicillin, and 100 µg/mL streptomycin. The cells were maintained at 37 °C in a humidified atmosphere of 95% air/5% CO_2_, and the medium was changed every 3 days. The cells used for all experiments were taken between the 9th and 13th passages, to avoid phenotypic changes. The cultures were passaged weekly by trypsinization (0.25% trypsin). In all experiments, the cells were seeded at the density of 2.5 × 10^5^ cells/mL in a 96-well plate and allowed to proliferate for 24 h before treatment.

#### 3.3.4. Evaluation of the Cytotoxicity

The cytotoxicity profile of the compounds under study was evaluated in HepG2 cells (2.5 × 10^5^ cells/mL) seeded in 96-well plates. The cells were then exposed to three different concentrations of the test compounds (2, 16, or 32 µg/mL) for 24 h, and cytotoxicity was evaluated using the NR uptake [86,87]. Control experiments were performed for viability endpoints by adding the vehicle (medium with 0.1% DMSO) instead of the compound solution. 

In the NR uptake assay, following a 24 h incubation period with the test compounds, the cell culture medium was aspirated, and fresh medium containing 150 µL of the dye (at a concentration of 50 mg/mL) was added to the cells. The cells were then incubated at 37 °C in a humified 5% CO_2_/95% air atmosphere for 2 h. After incubation, the cell culture medium was discarded, and a mixture of absolute ethanol and distilled water (1:1) containing 5% acetic acid was added to extract the dye accumulated within the lysosomes of viable cells. The absorbance at 540 nm was measured using a microplate reader (Synergy HTX Multi-Mode Reader—BioTek, Winooski, VT, USA). 

For all assays, results are expressed as a percentage of the control (nontreated cells), taken as 100%. Results are presented as means ± standard deviation (SD) of four independent experiments [86,87]. 

#### 3.3.5. Data Analysis and Statistics

The experimental data were analyzed using the statistical program GraphPad Prism 8.4.0 (GraphPad Software—La Jolla, CA, USA). At least three independent experiments with replicates were performed for each tested condition. The mean and standard deviation within samples were calculated for all cases. The normality of the data distribution was evaluated using three normality tests: the KS normality test, D’Agostino & Pearson omnibus normality test, and Shapiro–Wilk normality test. For data with a parametric distribution, statistical comparisons between groups were estimated using the parametric method one-way analysis of variance (ANOVA), followed by Dunnett’s multiple comparison test. For data with a non-parametric distribution, statistical comparisons were estimated using the nonparametric method of Kruskal–Wallis [one-way ANOVA on ranks] followed by Dunn’s *post hoc* test. Statistically significant differences were established for a probability level of 5% (*p* < 0.05).

## 4. Conclusions

In this study, a library of 49 structurally related QHSs was designed, synthesized, and evaluated for their antibacterial activity against *S. aureus,* including antibiotic-resistant strains. Intentionally including MDR *S. aureus* bacteria sought to assess QHS efficacy against the most challenging bacterial infections, addressing a critical public health aspect. To our knowledge, this is the first systematic SAR study of different types of QHSs adopting standard assays for antimicrobial susceptibility testing. The systematic exploration of SAR involved varying alkyl chain lengths and cation types, providing unique insights into the factors influencing antibacterial activity. The QHSs were obtained in moderate-to-high yields through a simple, solvent-free nucleophilic substitution reaction. The structural elucidation by NMR (^1^H, ^13^C, and DEPT–135) spectroscopic techniques confirmed the compounds’ structure and established a valuable database for accurate identification. Triphenylphosphonium and methylimidazolium derivatives exhibited superior activity against *S. aureus* CECT 976 among the cationic counterparts. QHSs with hexyl alkyl side-chains exhibited a detrimental effect on antibacterial activity, rendering them inactive; however, QHSs with tetradecyl side-chains demonstrated the highest efficiency, highlighting the central role of this chain length. The SAR study revealed a clear dependence on the length of the alkyl side-chain of the cation and its structure, which overall points to their lipophilicity and the consequent ease of penetrating the cell membrane. Notably, there appears to be a limit to the increase in antibacterial activity with the increment of the alkyl side-chain length, beyond which a plateau or even decreased activity occurs. The QHSs, particularly compounds **1e**, **3e**, and **5e**, exhibited high antibacterial activity at low concentrations, emphasizing their potential for control microbial growth. Furthermore, by correlating the MIC and in vitro human cellular data, it was determined that compounds **2e**, **4e**, and **5e** possess a favourable safety profile at concentrations equal to or below 2 µg/mL. Due to their potency, these compounds hold promise as effective agents against both susceptible and resistant bacterial strains. They can provide an invaluable foundation for the identification and design of antimicrobial QHSs tailored to counteract MDR bacteria.

## Figures and Tables

**Table 1:** Retention times (t_R_) obtained by LC/UV at pH 2.6, calculated CHI, and CHI LogP parameters for all QHSs tested.

Compound	t_R_/min	CHI ^1^	CHI LogP ^2^
**1a**	6.599	60.58	1.747
**1b**	7.096	71.06	2.240
**1c**	7.642	82.58	2.781
**1d**	8.207	94.50	3.341
**1e**	8.706	105.0	3.836
**1f**	9.202	115.5	4.328
**1g**	9.806	128.2	4.926
**2a**	5.118	29.35	0.2794
**2b**	5.727	42.19	0.8830
**2c**	6.311	54.51	1.462
**2d**	6.915	67.25	2.061
**2e**	7.584	81.36	2.724
**2f**	8.274	95.91	3.408
**2g**	8.906	109.2	4.034
**3a**	5.519	37.81	0.6769
**3b**	6.098	50.02	1.251
**3c**	6.648	61.62	1.796
**3d**	7.258	74.48	2.401
**3e**	7.898	87.98	3.035
**3f**	8.508	100.8	3.640
**3g**	9.087	113.1	4.214
**4a**	5.200	31.08	0.3607
**4b**	5.808	43.90	0.9633
**4c**	6.381	55.99	1.531
**4d**	6.996	68.96	2.141
**4e**	7.657	82.90	2.796
**4f**	8.315	96.77	3.448
**4g**	8.902	109.2	4.030
**5a**	5.480	36.98	0.6382
**5b**	6.061	49.24	1.214
**5c**	6.640	61.45	1.788
**5d**	7.245	74.21	2.388
**5e**	7.899	88.00	3.036
**5f**	8.519	101.1	3.651
**5g**	9.081	112.9	4.208
**6a**	5.010	27.07	0.1723
**6b**	5.660	40.78	0.8166
**6c**	6.243	53.07	1.395
**6d**	6.836	65.58	1.982
**6e**	7.515	79.90	2.655
**6f**	8.206	94.47	3.340
**6g**	8.853	108.1	3.982

^1^ CHI values were calculated using the equation obtained in the linear correlation (Figure S50, Supplementary Materials). ^2^ CHI logP were back-calculated using the equation CHI LogP = 0.047 × CHI + 0.36 × HBC − 1.10.

**Table 2:** Minimum inhibitory (MIC) and minimum bactericidal (MBC) concentration values (µg/mL) of quaternary salts and common antibiotics against *S. aureus* CECT 976.

Compound	CECT 976	
	MIC ^1^	MBC ^2^
**1a**	8	>64
**1b**	2	16
**1c**	1	8
**1d**	1	16
**1e**	1	16
**1f**	2	16
**1g**	4	64
**2a**	>64	>64
**2b**	>64	>64
**2c**	16	>64
**2d**	1	16
**2e**	1	8
**2f**	4	8
**2g**	8	16
**3a**	>64	>64
**3b**	64	>64
**3c**	16	64
**3d**	2	16
**3e**	2	8
**3f**	4	8
**3g**	8	16
**4a**	>64	>64
**4b**	>64	>64
**4c**	32	>64
**4d**	2	16
**4e**	2	8
**4f**	4	8
**4g**	8	16
**5a**	>64	>64
**5b**	16	>64
**5c**	16	32
**5d**	4	16
**5e**	2	8
**5f**	4	8
**5g**	16	16
**6a**	>64	>64
**6b**	>64	>64
**6c**	32	>64
**6d**	2	8
**6e**	4	8
**6f**	4	8
**6g**	8	16
**7a**	>64	>64
**7b**	>64	>64
**7c**	64	>64
**7d**	4	16
**7e**	2	16
**7f**	64	64
**7g**	16	16
[ERY]	0.24	ND
[TET]	0.96	ND
[CIP]	1	ND
[AMP]	1.5	ND
[OXA]	0.48	ND

^1^ MIC: minimum inhibitory concentration (µg/mL). ^2^ MBC: minimum bactericidal concentration (µg/mL). For concentrations higher than 64 µg/mL, MIC and MBC were not tested. ND: not determined. [ERY]: erythromycin; [TET]: tetracycline; [CIP]: ciprofloxacin; [AMP]: ampicillin; [OXA]: oxacillin. The MIC values of antibiotics reported here were obtained from the study conducted by Abreu et al. [44] Both the MIC values of antibiotics and quaternary salts were determined using the broth microdilution method, following the approved M7-A7 (aerobes) standards outlined in the Clinical and Laboratory Standards Institute (CLSI) guidelines [45]. All tests were performed with six replicates and a minimum of three independent repeats.

**Table 3:** Minimum inhibitory (MIC) and minimum bactericidal (MBC) concentration values (µg/mL) of the most active quaternary salts and common antibiotics against antibiotic-resistant *S. aureus* XU212, SA1199B, and RN4220 strains.

Compound	XU212		SA1199B		RN4220	
	MIC ^1^	MBC ^2^	MIC ^1^	MBC ^2^	MIC ^1^	MBC ^2^
**1e**	2	2	1	2	1	2
**2e**	32	64	8	32	4	8
**3e**	4	8	2	8	2	4
**4e**	32	32	8	32	4	8
**5e**	8	8	4	8	2	8
**6e**	32	64	16	16	4	8
**7e**	32	32	16	32	4	8
[ERY]	ND	ND	ND	ND	256	ND
[TET]	128	ND	ND	ND	ND	ND
[CIP]	ND	ND	128	ND	ND	ND

^1^ MIC: minimum inhibitory concentration (µg/mL). ^2^ MBC: minimum bactericidal concentration (µg/mL). ND: not determined. [ERY]: erythromycin; [TET]: tetracycline; [CIP]: ciprofloxacin. The MIC values of antibiotics reported here were obtained from the study conducted by Abreu et al. [44]. Both the MIC values of antibiotics and quaternary salts were determined using the broth microdilution method, following the approved M7-A7 (aerobes) standards outlined in the Clinical and Laboratory Standards Institute (CLSI) guidelines [45]. All tests were performed with six replicates and a minimum of three independent repeats.

**Table 4:** Evaluation of lysosomal activity in HepG2 cells after 24 h of exposure to the most active quaternary salts and common antibiotics.

Compound	Concentration (µg/mL)			
	1	2	16	32
**1e**	53.86 ± 4.854% (****)	72.84 ± 7.906% (****)	12.29 ± 3.775% (****)	13.78 ± 2.666% (****)
**2e**	90.88 ± 10.08% (**)	86.71 ± 4.103% (****)	12.85 ± 2.005% (****)	13.64 ± 2.776% (****)
**3e**	ND	79.86 ± 9.893% (****)	12.68 ± 2.385% (****)	12.77 ± 1.368% (****)
**4e**	ND	92.62 ± 4.355% (*)	14.45 ± 2.437% (****)	21.52 ± 19.06% (****)
**5e**	ND	88.20 ± 3.101% (****)	12.06 ± 2.988% (****)	14.38 ± 4.045% (****)
**6e**	ND	94.21 ± 10.35% (ns)	10.46 ± 3.129% (****)	12.93 ± 3.760% (****)
**7e**	ND	86.24 ± 1.502% (****)	18.51 ± 2.592% (****)	16.59 ± 4.378% (****)
[ERY]	ND	108.4 ± 4.998% (ns)	107.1 ± 7.642% (ns)	110.2 ± 9.546% (ns)
[TET]	ND	109.0 ± 5.251% (ns)	107.5 ± 9.694% (ns)	106.8 ± 6.699% (ns)
[CIP]	ND	106.7 ± 11.16% (ns)	96.82 ± 5.237% (ns)	92.35 ± 6.807% (ns)

ND: not determined. [ERY]: erythromycin; [TET]: tetracycline; [CIP]: ciprofloxacin. All values are expressed as mean ± standard deviation (SD) of four independent experiments. Statistical comparisons were made using one-way ANOVA. In all cases, *p* values lower than 0.05 were considered significant (* *p* < 0.005, ** *p* < 0.01, **** *p* < 0.0001).

## Data Availability

The data presented in this study are available on request from the corresponding author. The data are not publicly available due to intellectual property concerns.

## Supplementary Materials

The following supporting information can be downloaded at: https://www.mdpi.com/article/10.3390/ijms25010504/s1. This supplementary material provides the structural characterizations of the synthesized QHSs using 1H, 13C, and DEPT135 spectra, along with details on the evaluation of the chromatographic hydrophobicity index.
