# Supplementary material for: Efficacy of Novel Quaternary Ammonium and Phosphonium Salts Differing in Cation Type and Alkyl Chain Length against Antibiotic-Resistant Staphylococcus aureus
Source: Int J Mol Sci. 2023 Dec 29;25(1):504. doi: 10.3390/ijms25010504 (PMC10778626; doi:10.3390/ijms25010504)
Supplement: Supplementary file 1 [file ijms-25-00504-s001.zip › ijms-2752554-supplementary.pdf]

Supplementary Materials

Efficacy of Novel Quaternary Ammonium and Phosphonium Salts Differing in Cation Type and Alkyl Chain Length against Antibiotic-Resistant *Staphylococcus aureus*

Bárbara Nunes [1,2,3], Fernando Cagide [3], Carlos Fernandes [3], Anabela Borges [1,2], Fernanda Borges [3] and Manuel Simões [1,2,*]

[1] LEPABE—Laboratory for Process Engineering, Environment, Biotechnology and Energy, Faculty of Engineering, University of Porto, Rua Dr. Roberto Frias, s/n, 4200-465 Porto, Portugal; up201804372@edu.fe.up.pt (B.N.); apborges@fe.up.pt (A.B.)
[2] ALiCE—Associate Laboratory in Chemical Engineering, Faculty of Engineering, University of Porto, Rua Dr. Roberto Frias, 4200-465 Porto, Portugal
[3] CIQUP-IMS, Department of Chemistry and Biochemistry, Faculty of Sciences, University of Porto, Rua do Campo Alegre, s/n, 4169-007 Porto, Portugal; carlos.fernandes@fc.up.pt (C.F.); fborges@fc.up.pt (F.B.)
* Correspondence: mvs@fe.up.pt

1. Experimental Section

1.1 Nuclear Magnetic Resonance Data

a)

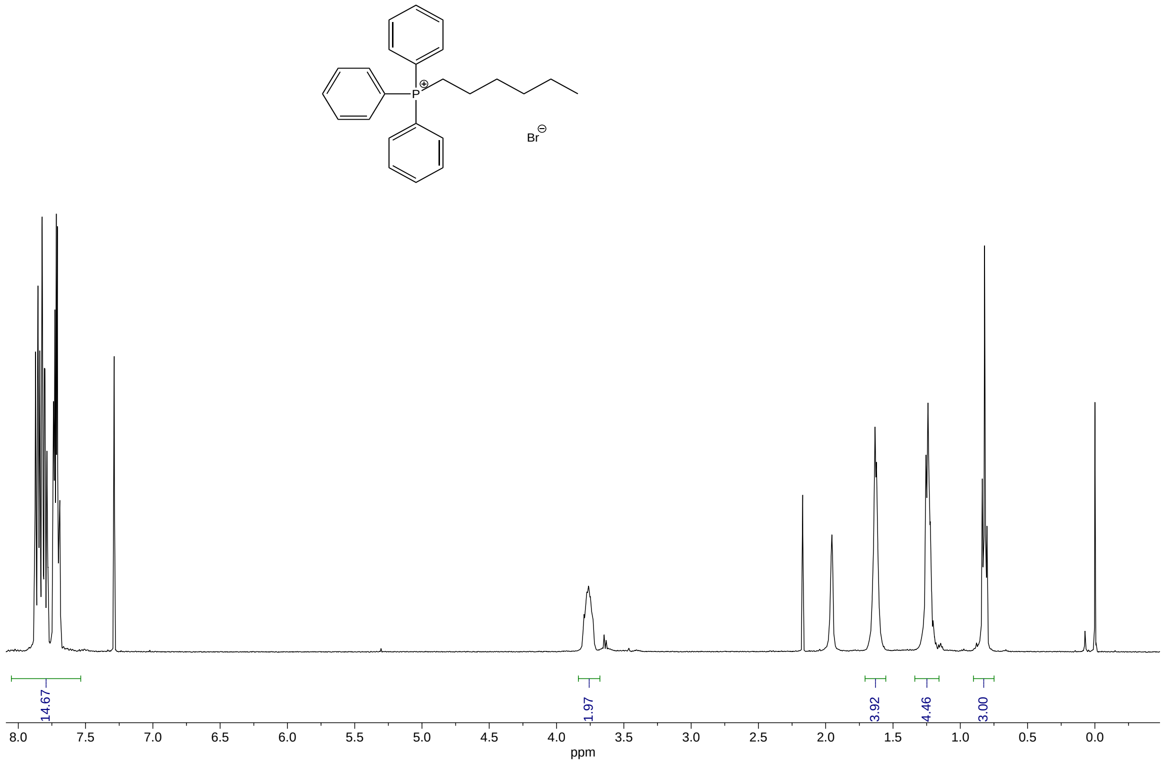

b)

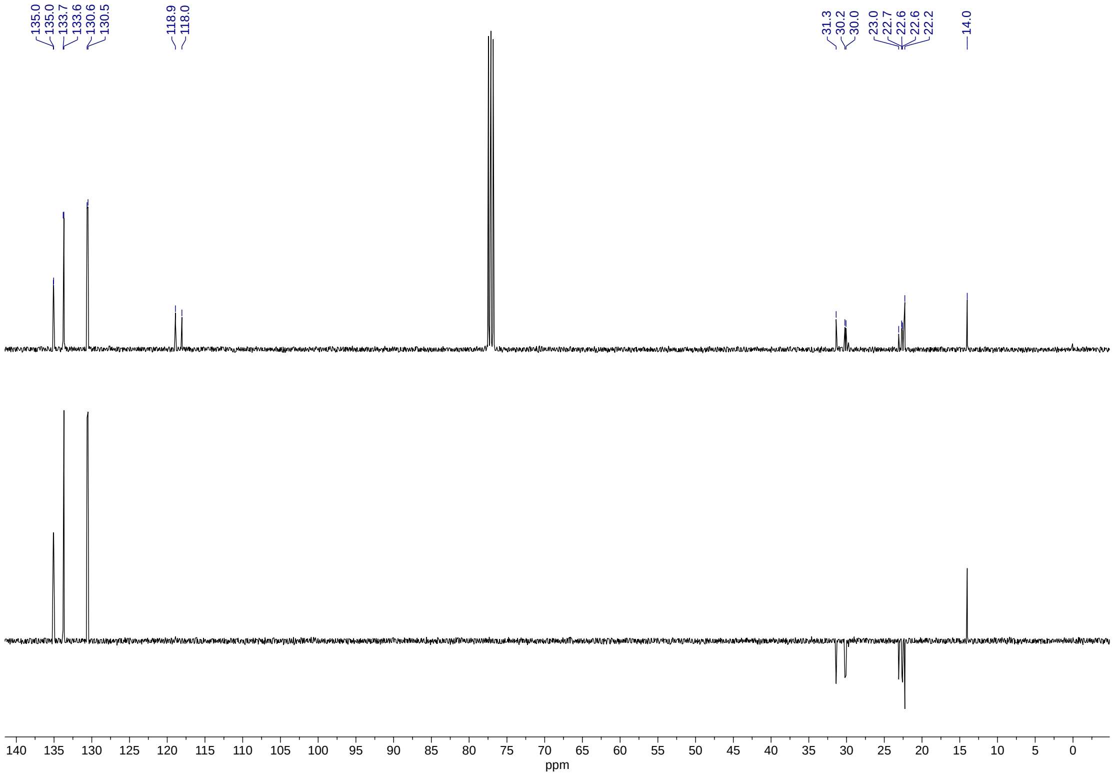

Figure S1. ^{1}H NMR **(a)**, ^{13}C NMR and DEPT135 **(b)** spectra of compound **1a**.

a)

b)

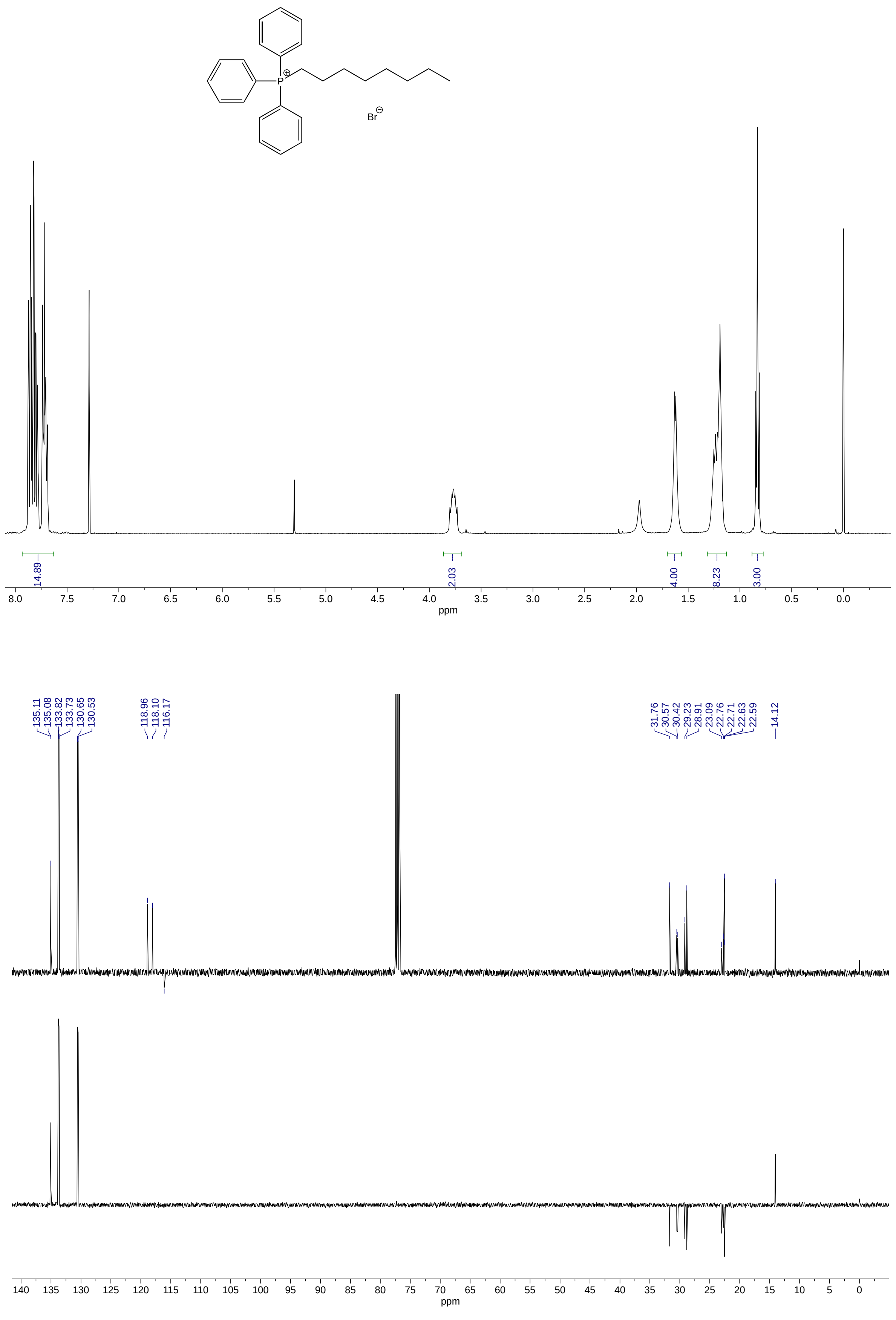

Figure S2. ^{1}H NMR **(a)**, ^{13}C NMR and DEPT135 **(b)** spectra of compound **1b**.

a)

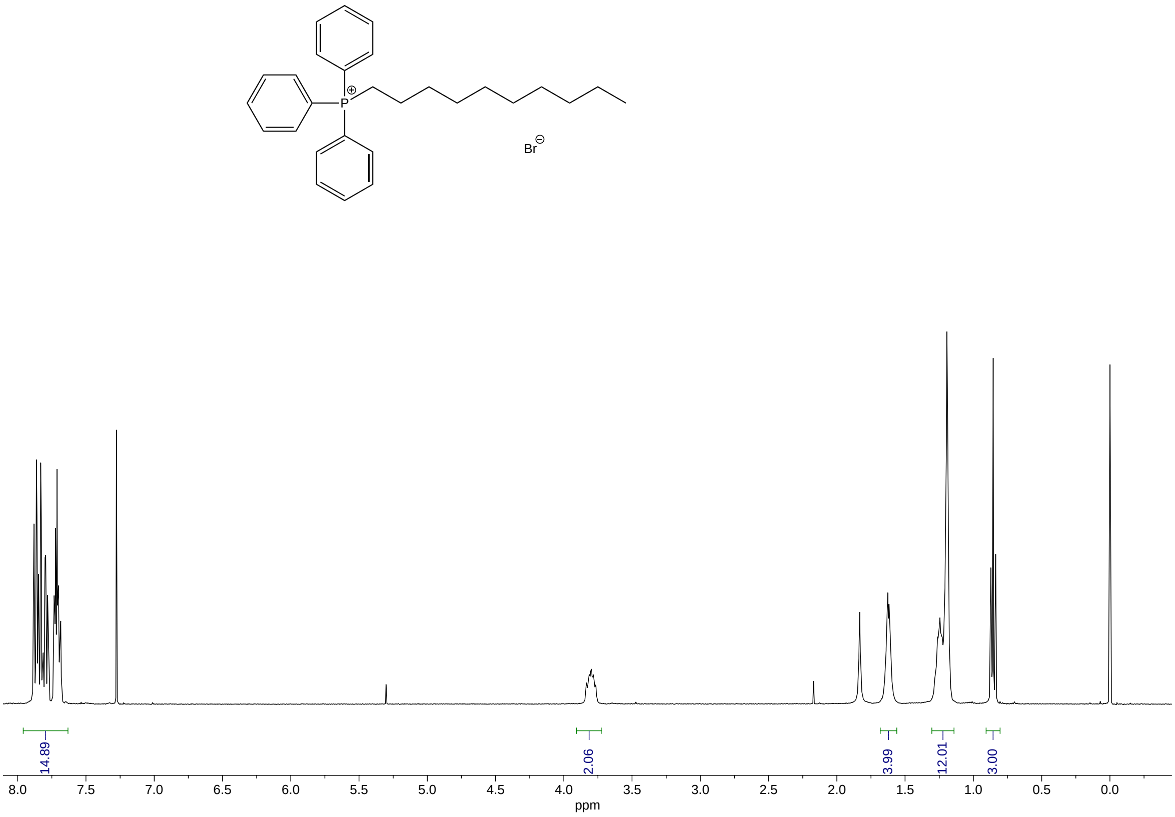

b)

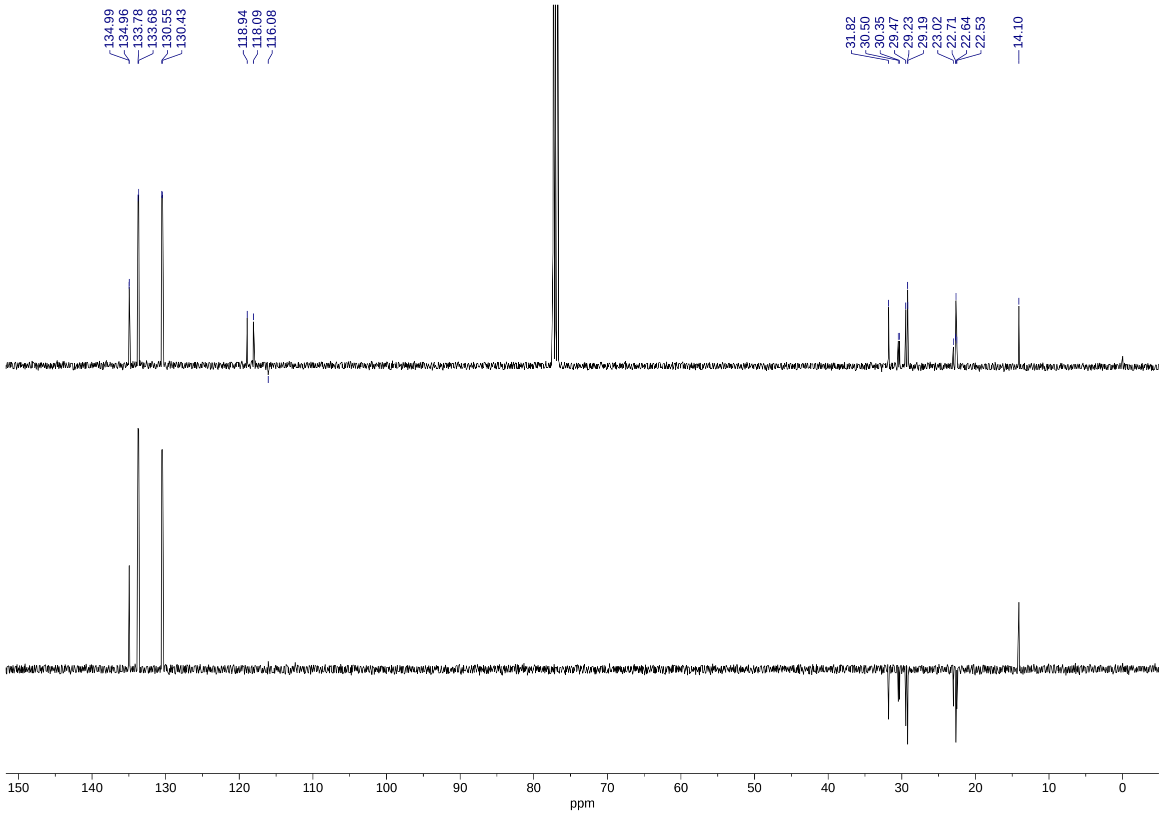

Figure S3. ^{1}H NMR **(a)**, ^{13}C NMR and DEPT135 **(b)** spectra of compound **1c**.

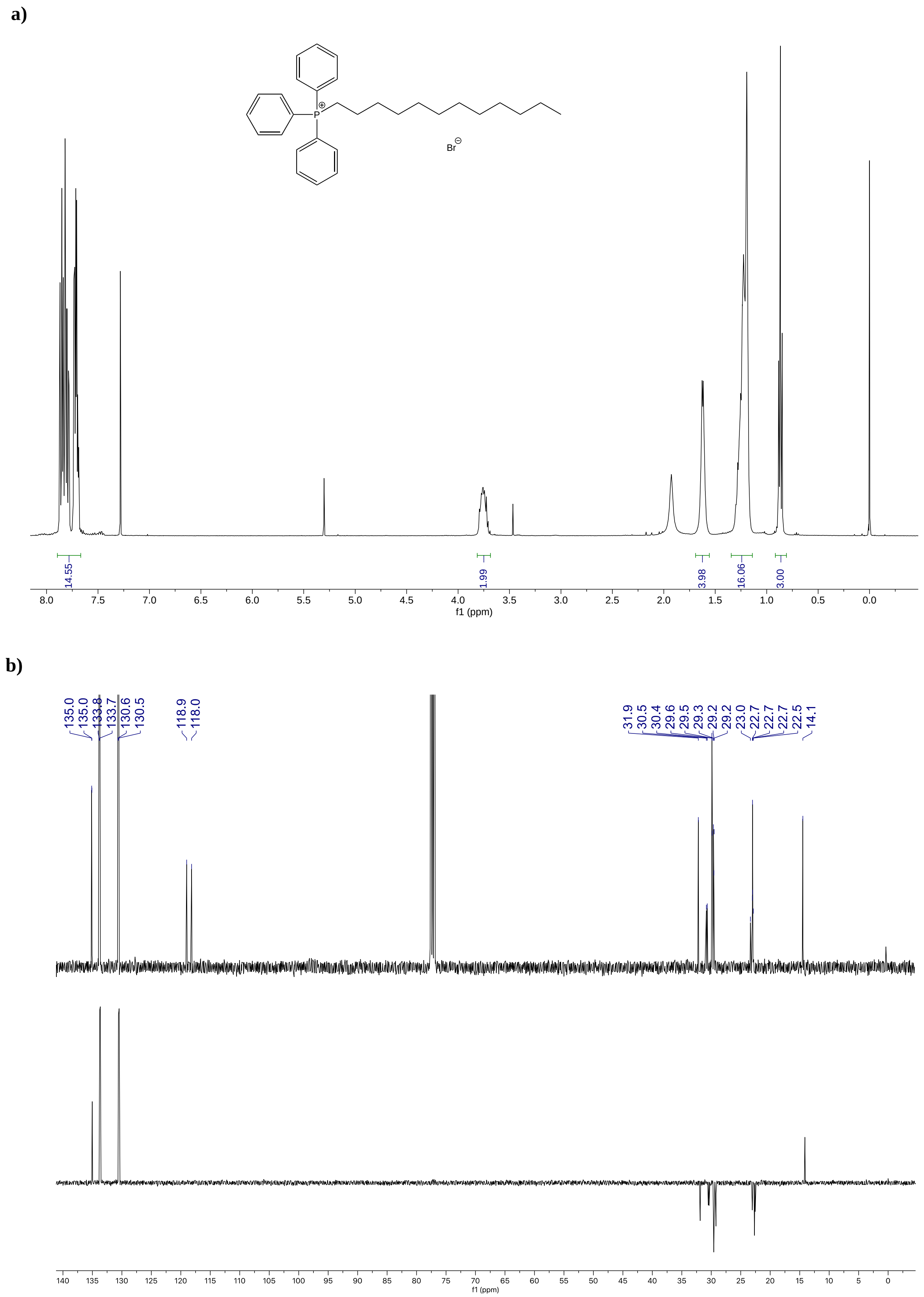

Figure S4. ^{1}H NMR **(a)**, ^{13}C NMR and DEPT135 **(b)** spectra of compound **1d**.

a)

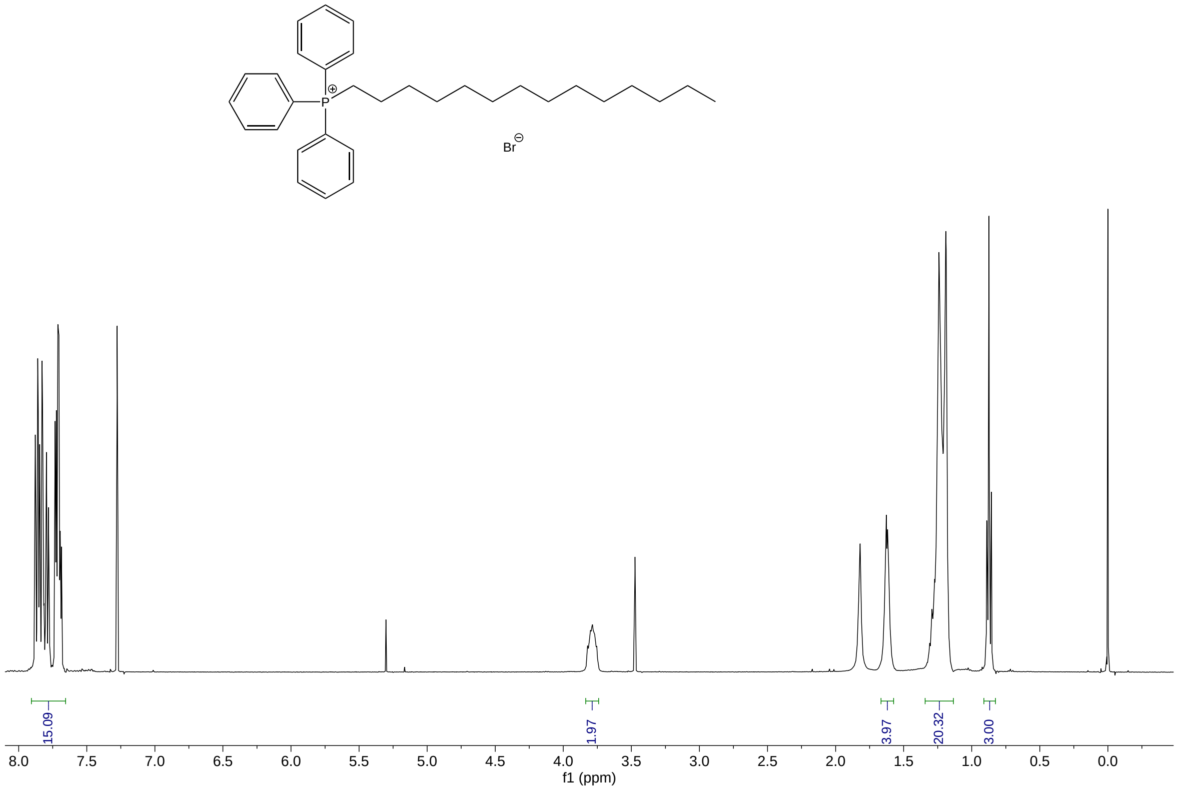

b)

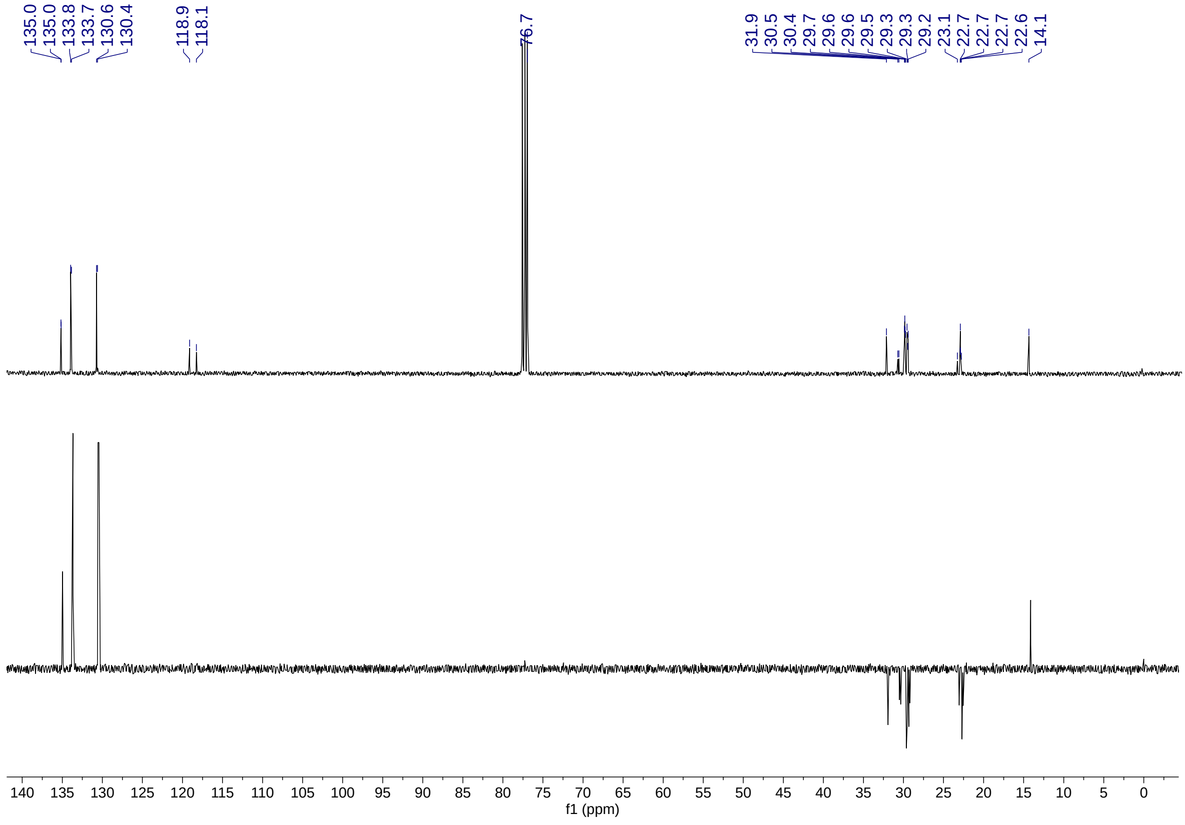

Figure S5. ^{1}H NMR **(a)**, ^{13}C NMR and DEPT135 **(b)** spectra of compound **1e**.

a)

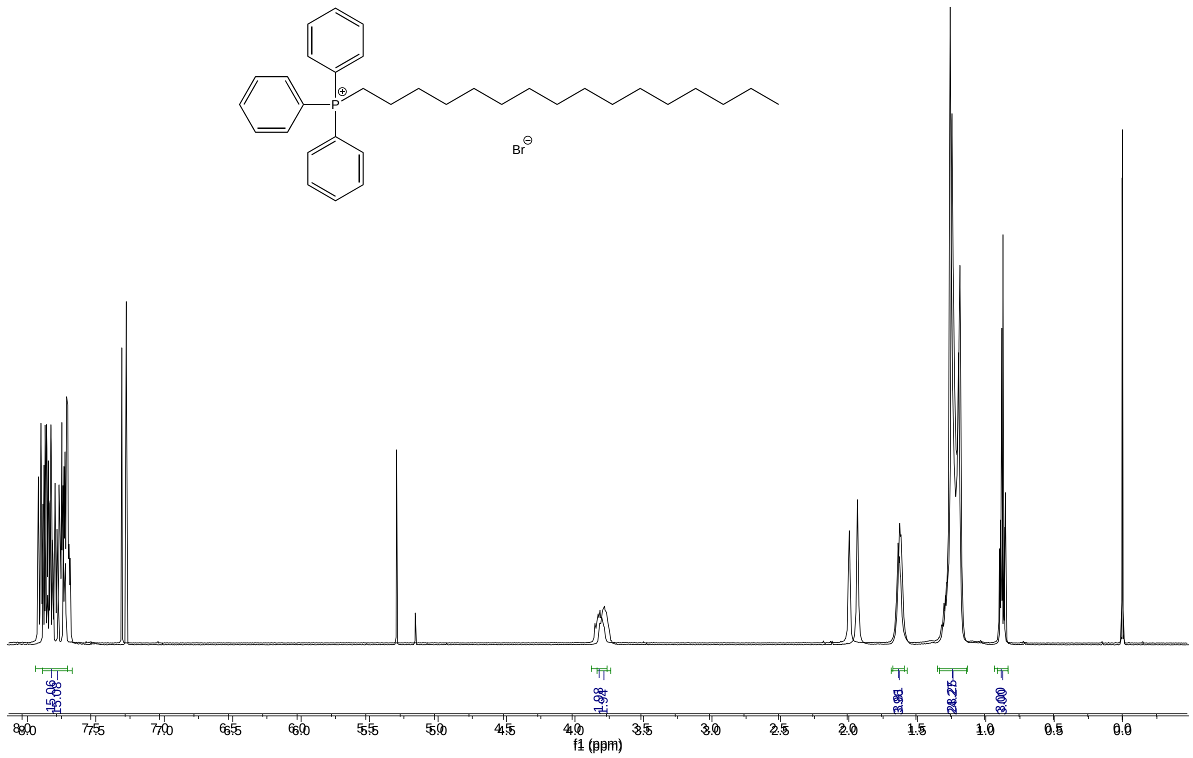

b)

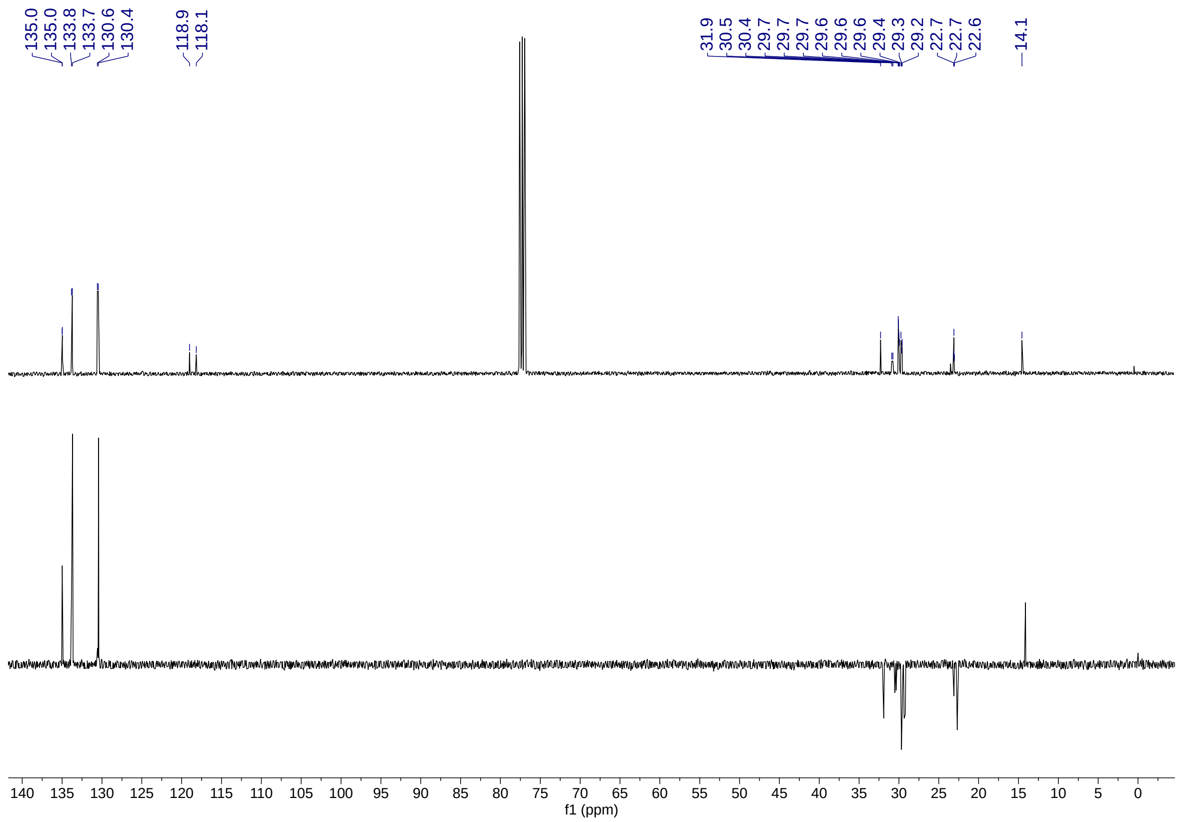

Figure S6. ^{1}H NMR **(a)**, ^{13}C NMR and DEPT135 **(b)** spectra of compound **1f**.

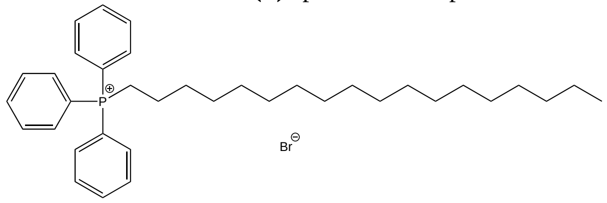

b)

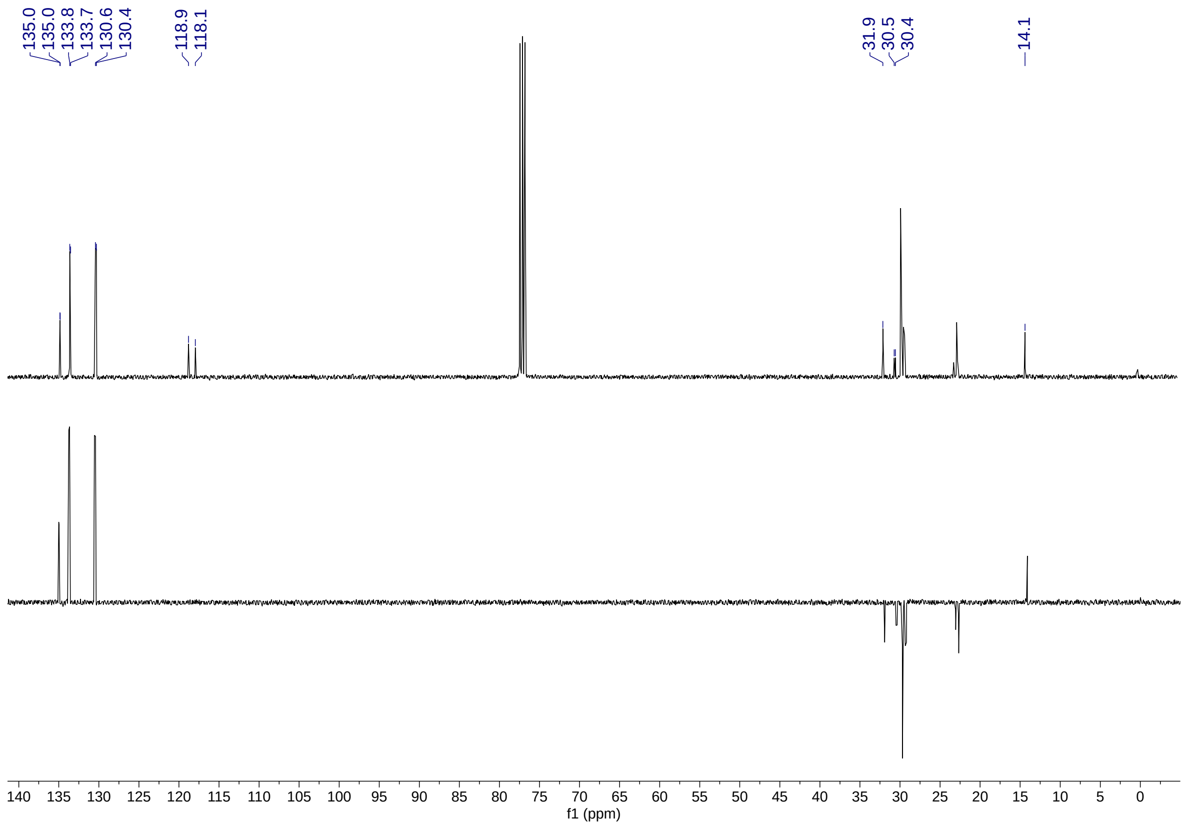

Figure S7. ^{1}H NMR **(a)**, ^{13}C NMR and DEPT135 **(b)** spectra of compound **1g**.

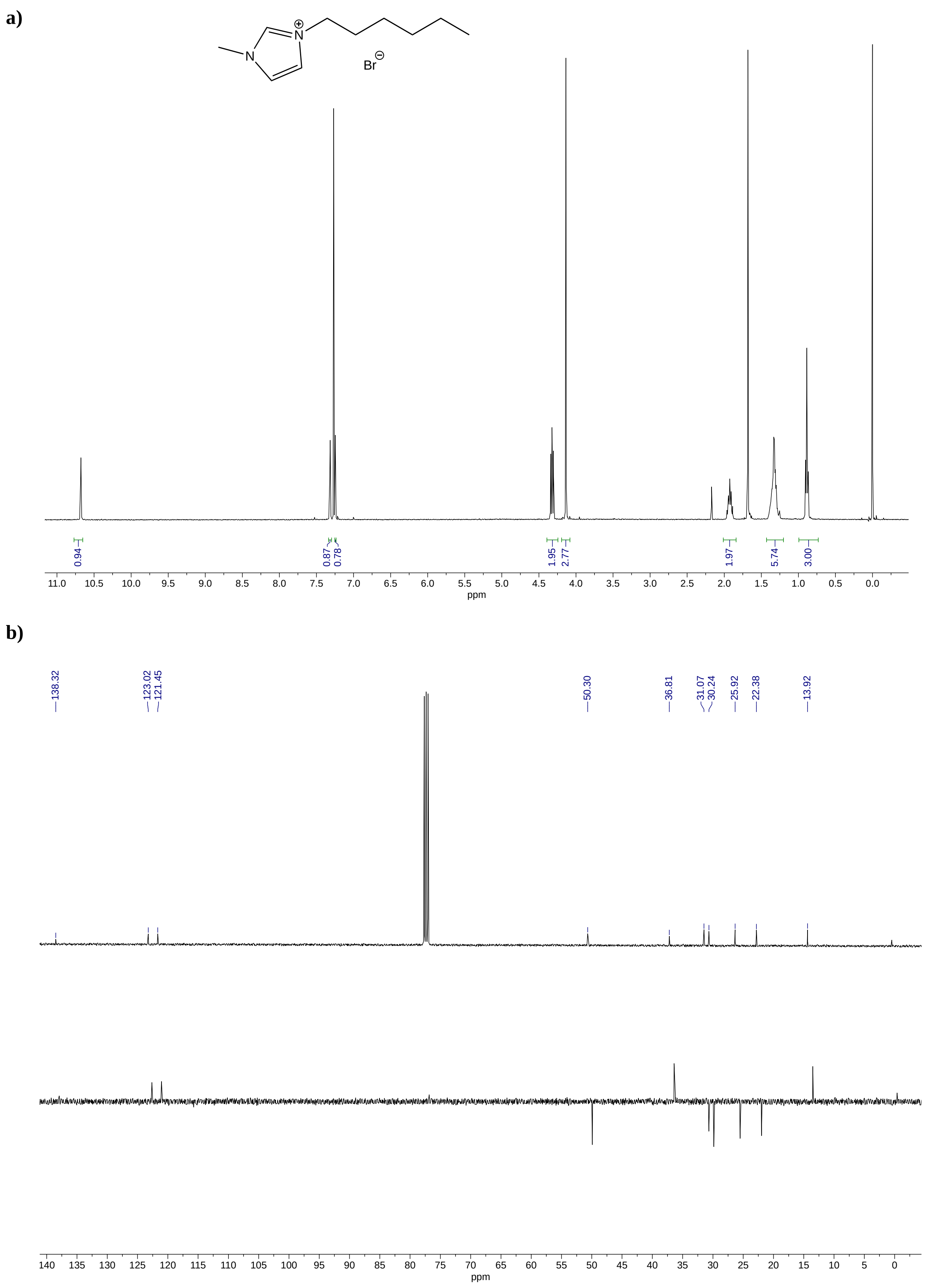

Figure S8. ^{1}H NMR **(a)**, ^{13}C NMR and DEPT135 **(b)** spectra of compound **2a**.

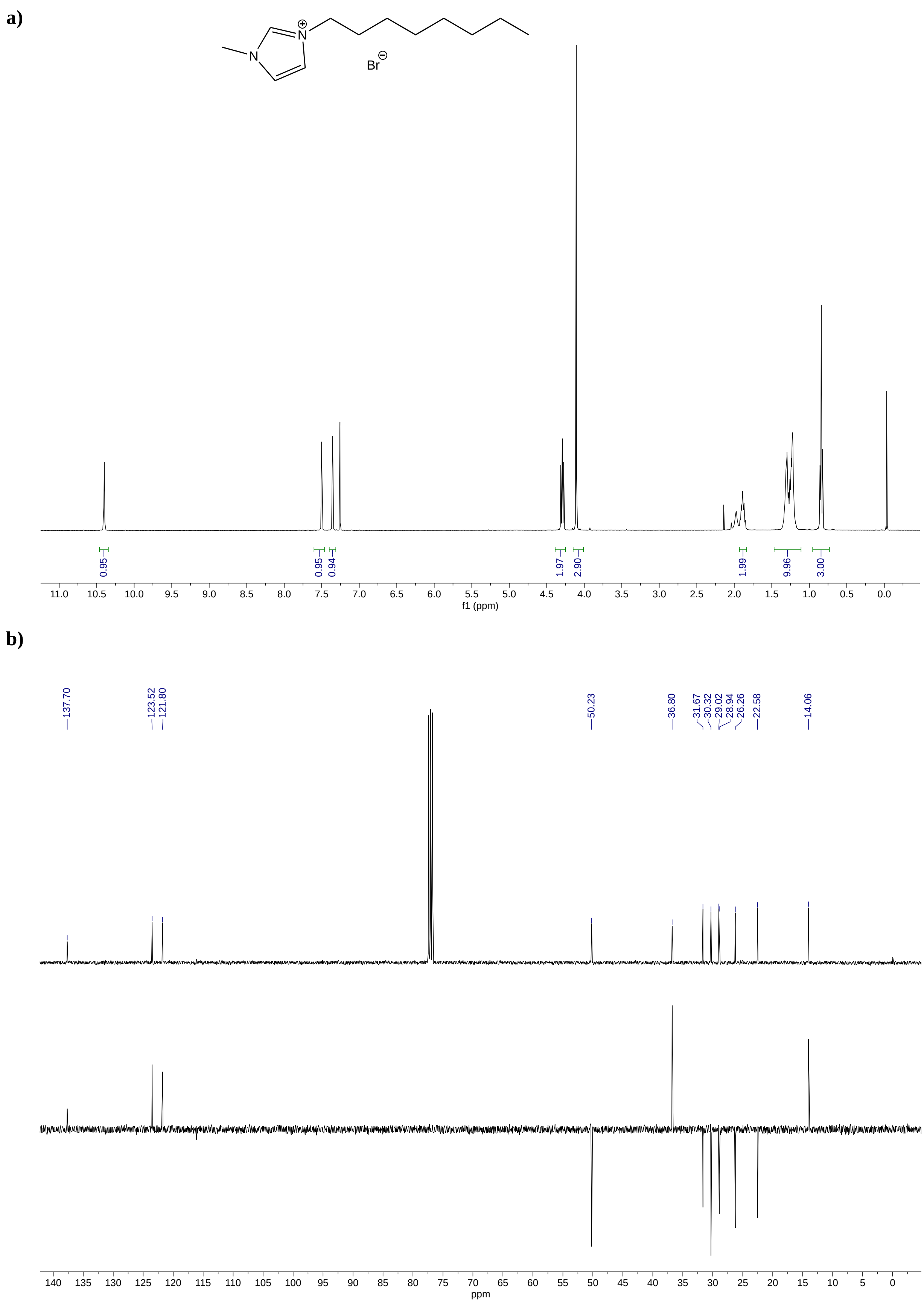

Figure S9. ^{1}H NMR **(a)**, ^{13}C NMR and DEPT135 **(b)** spectra of compound **2b**.

a)

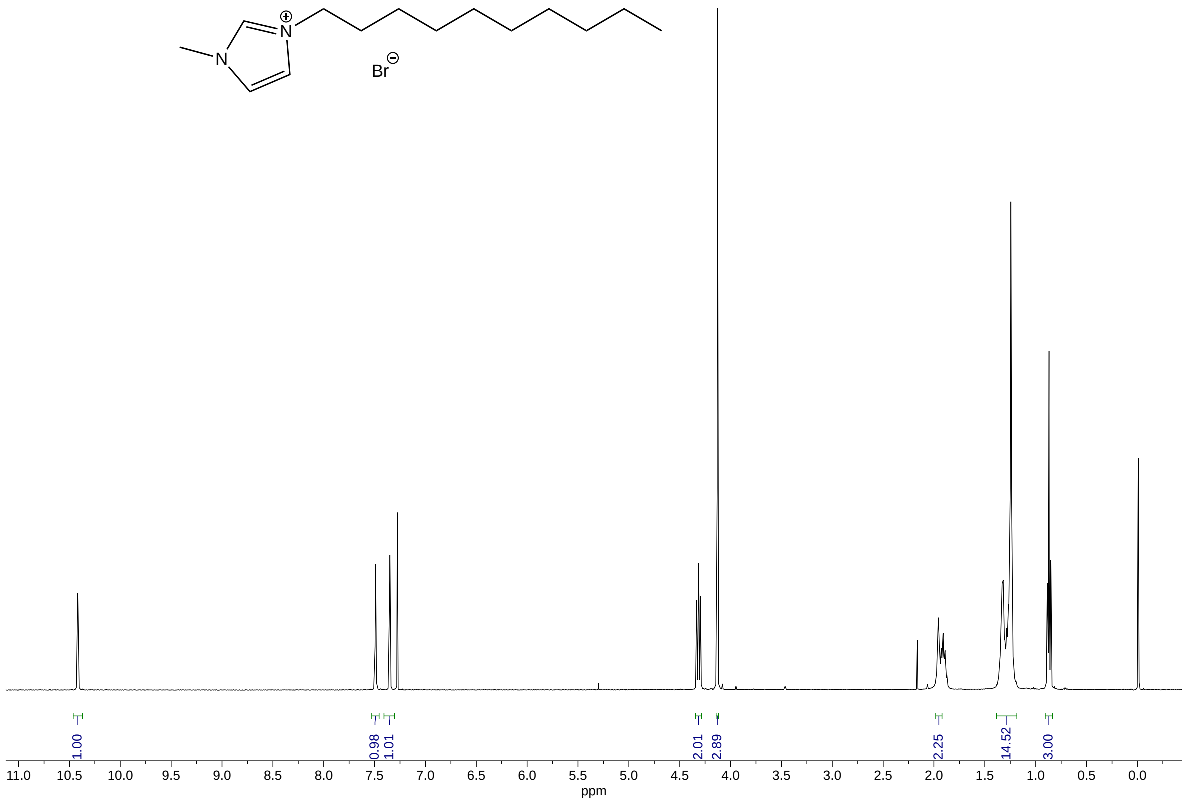

b)

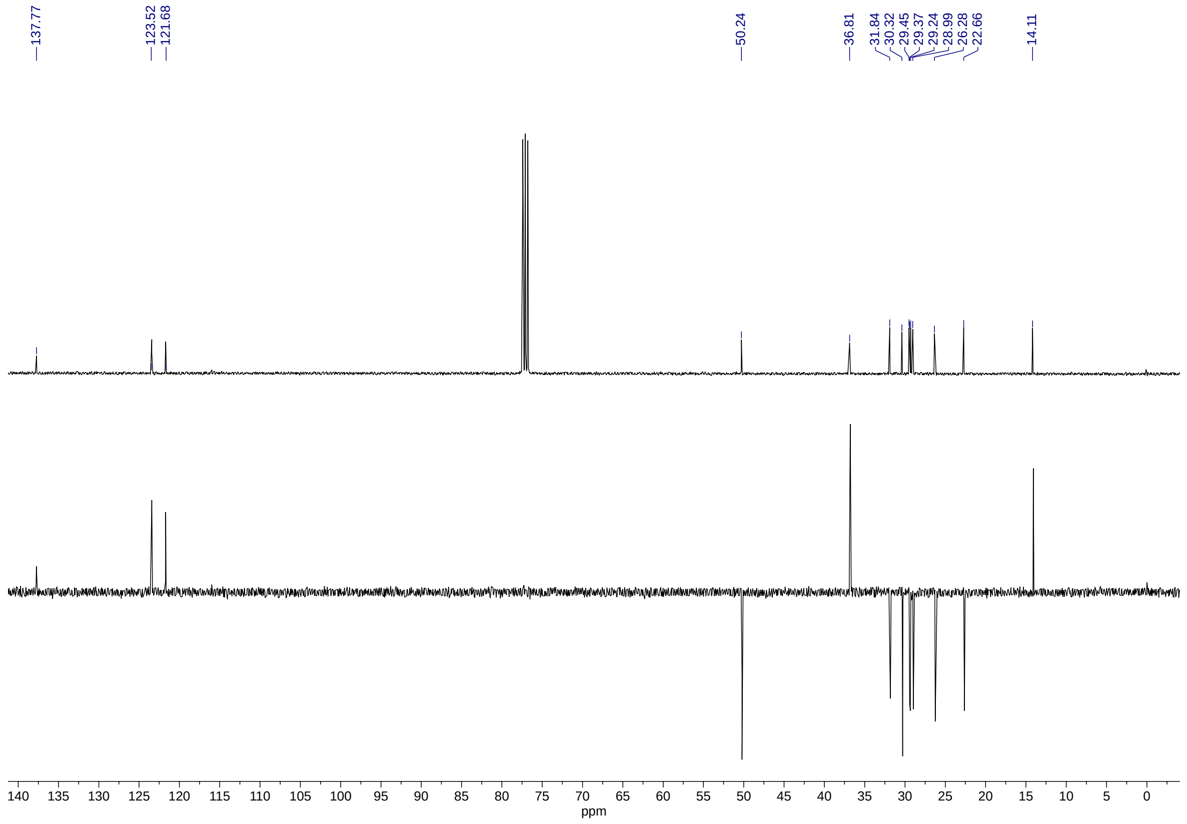

Figure S10. ^{1}H NMR **(a)**, ^{13}C NMR and DEPT135 **(b)** spectra of compound **2c**.

a)

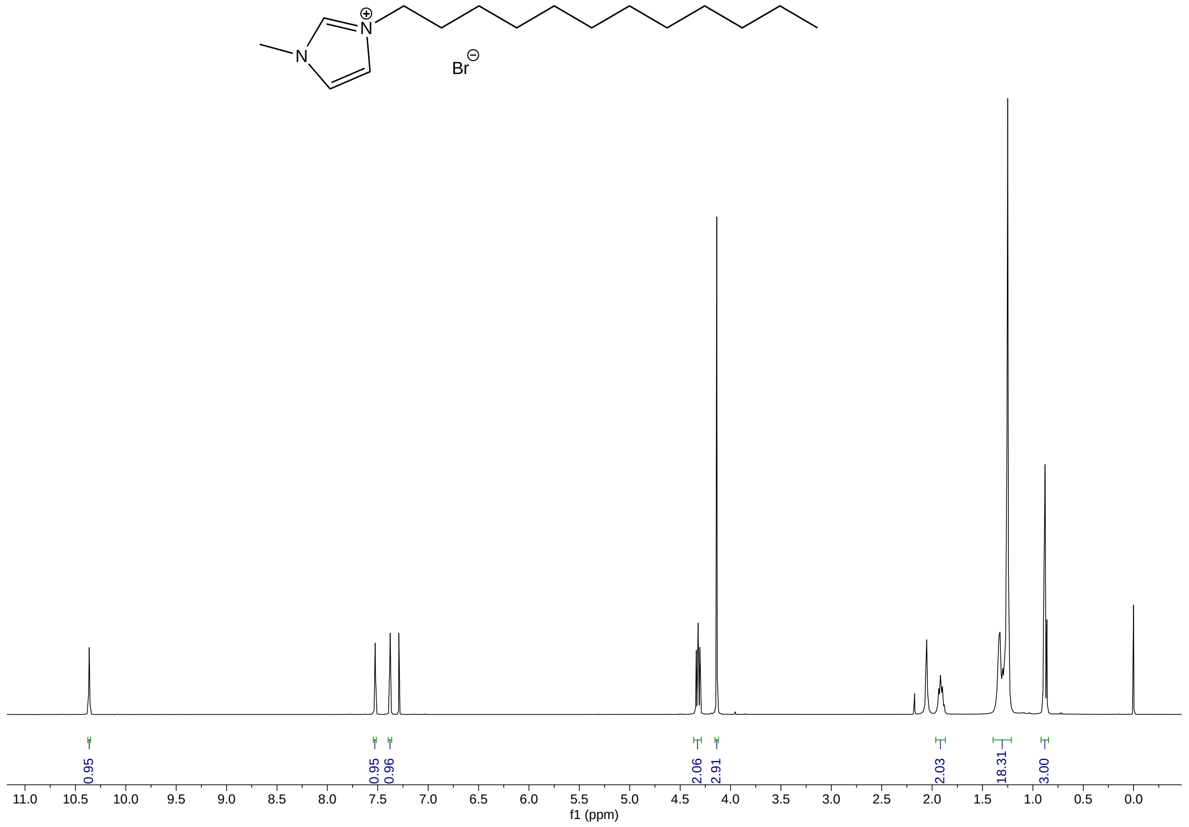

b)

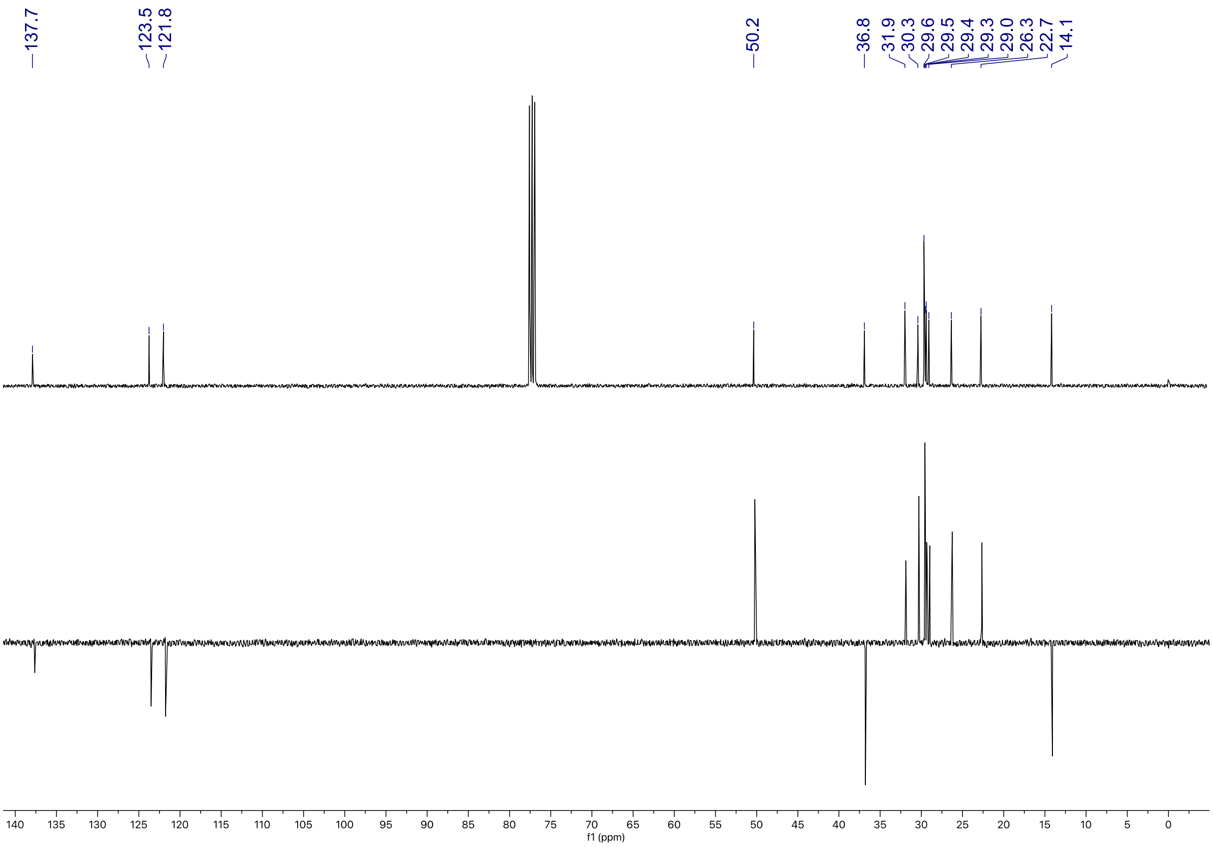

Figure S11. ^{1}H NMR **(a)**, ^{13}C NMR and DEPT135 **(b)** spectra of compound **2d**.

a)

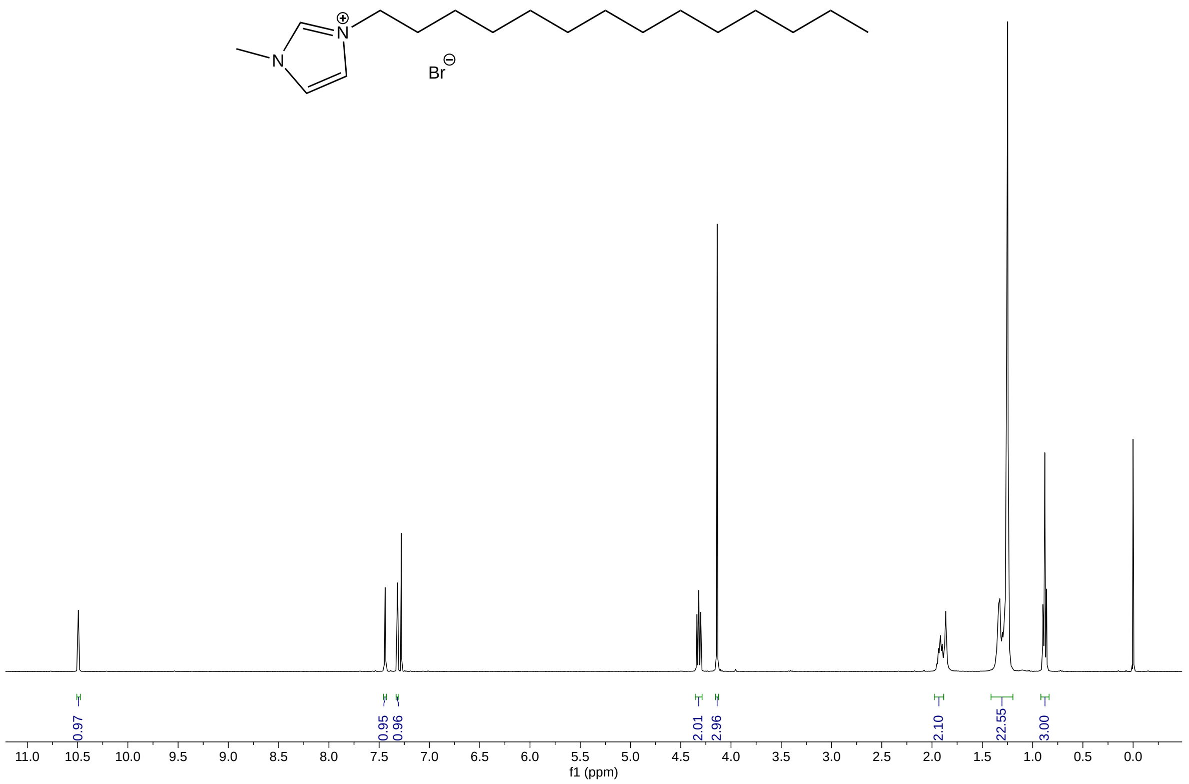

b)

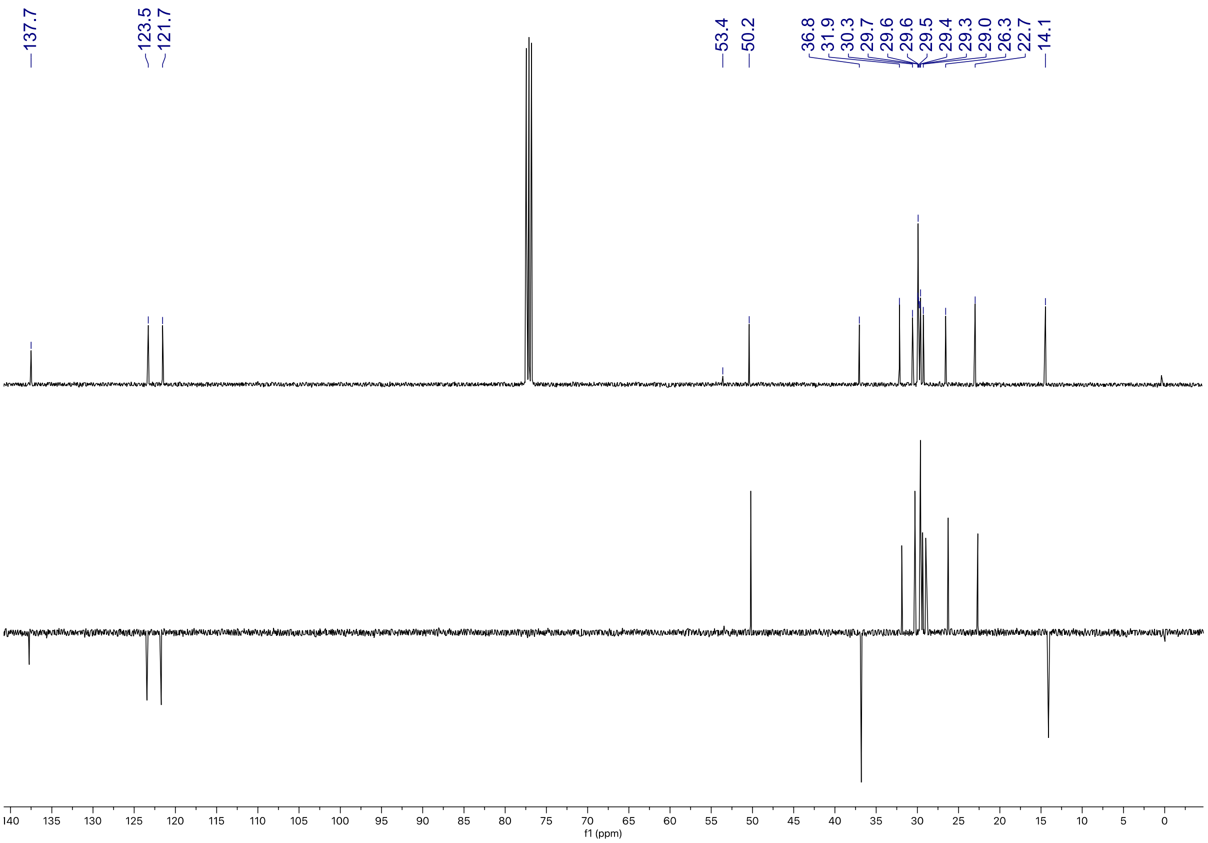

Figure S12. ¹H NMR **(a)**, ¹³C NMR and DEPT135 **(b)** spectra of compound **2e**.

a)

b)

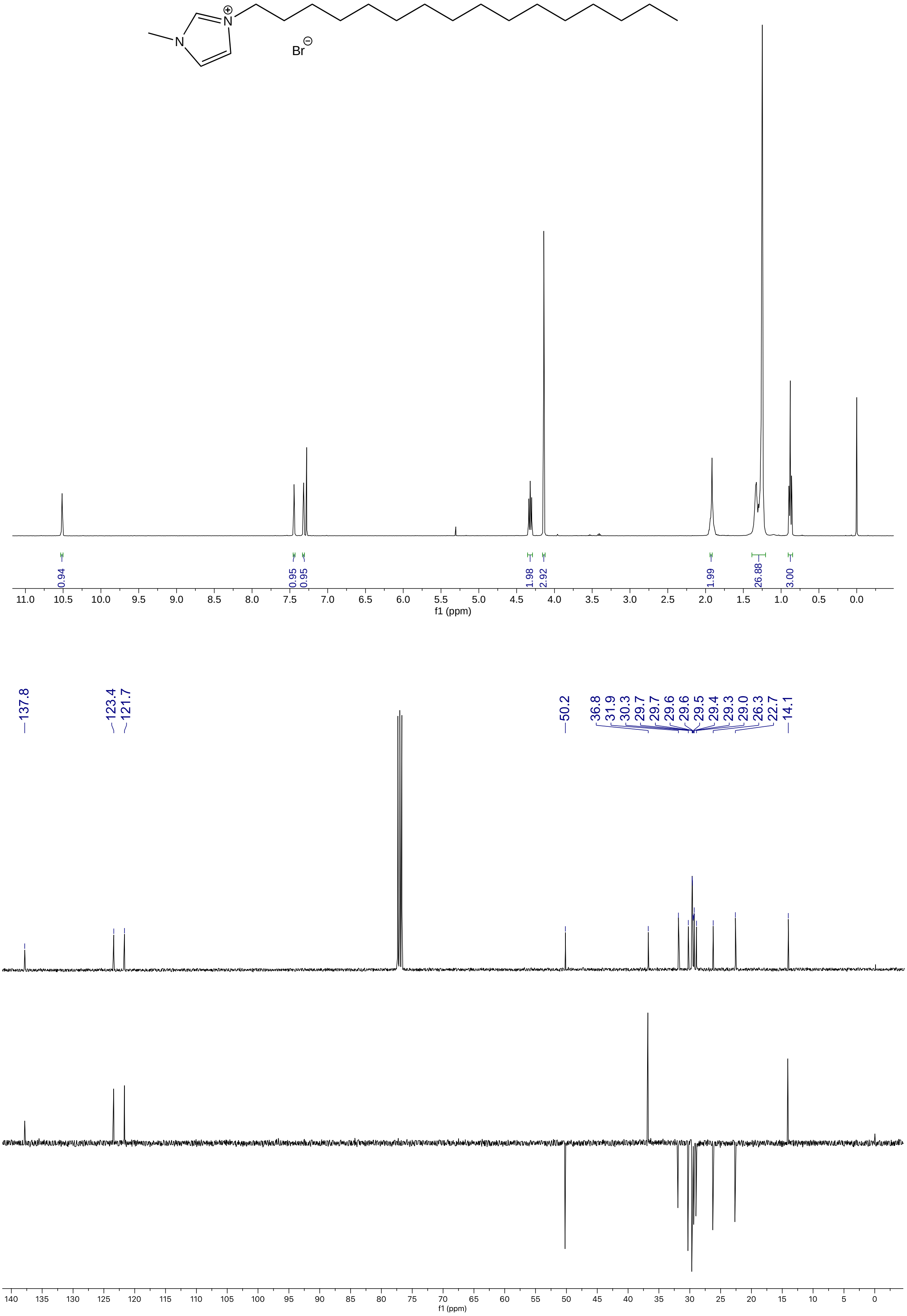

Figure S13. ^{1}H NMR **(a)**, ^{13}C NMR and DEPT135 **(b)** spectra of compound **2f**.

a)

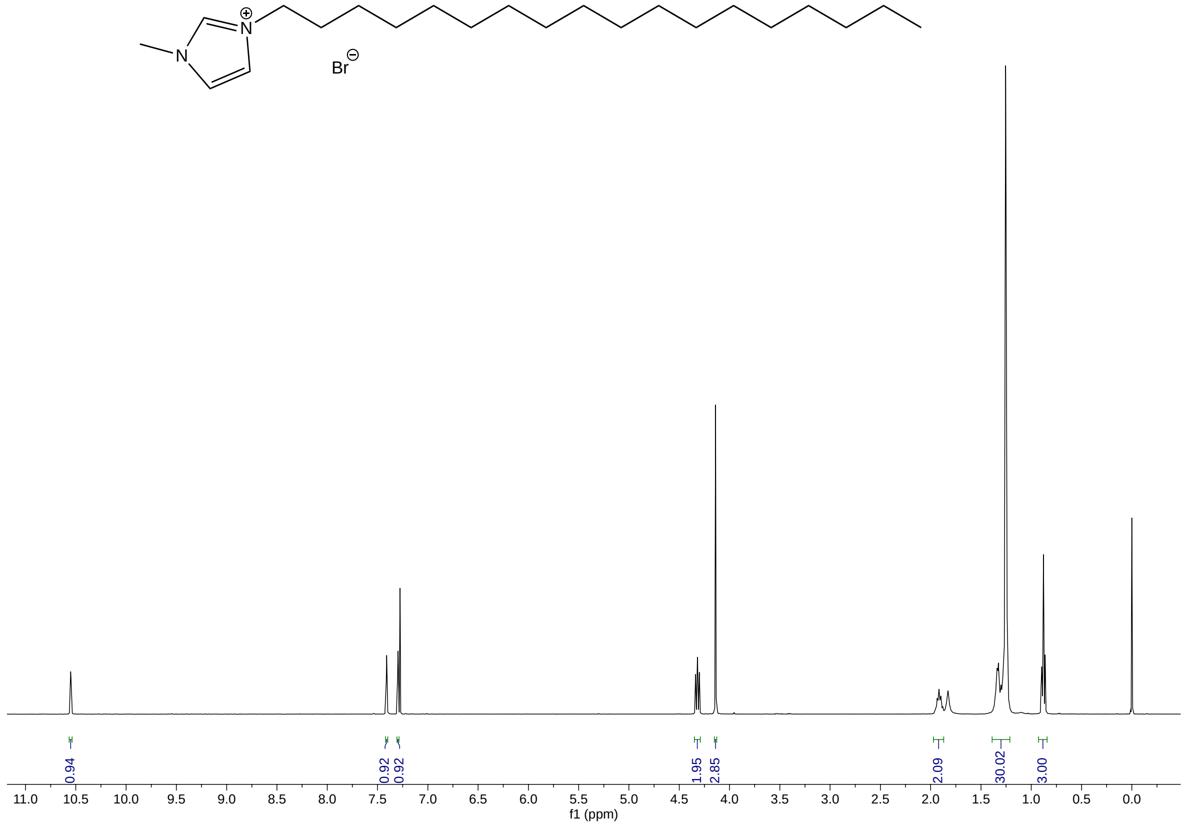

b)

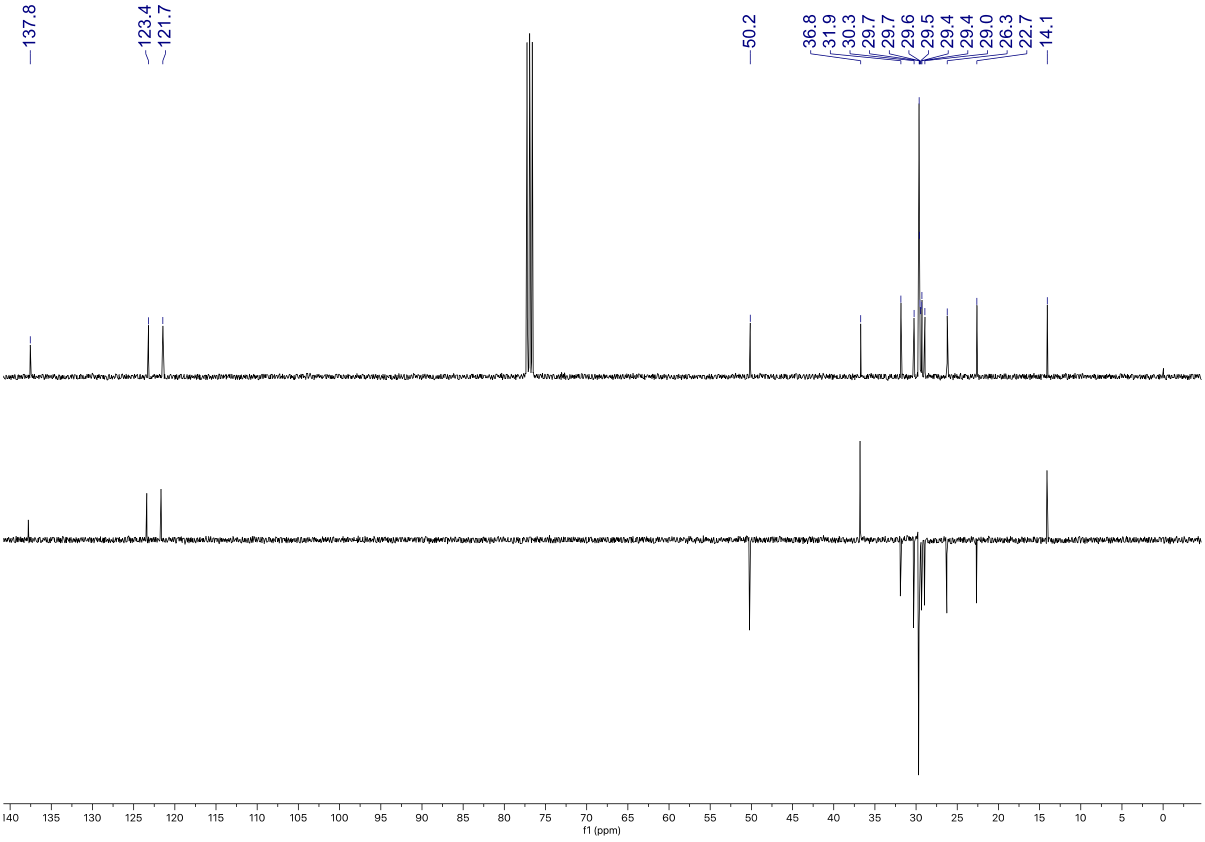

Figure S14. ^{1}H NMR **(a)**, ^{13}C NMR and DEPT135 **(b)** spectra of compound **2g**.

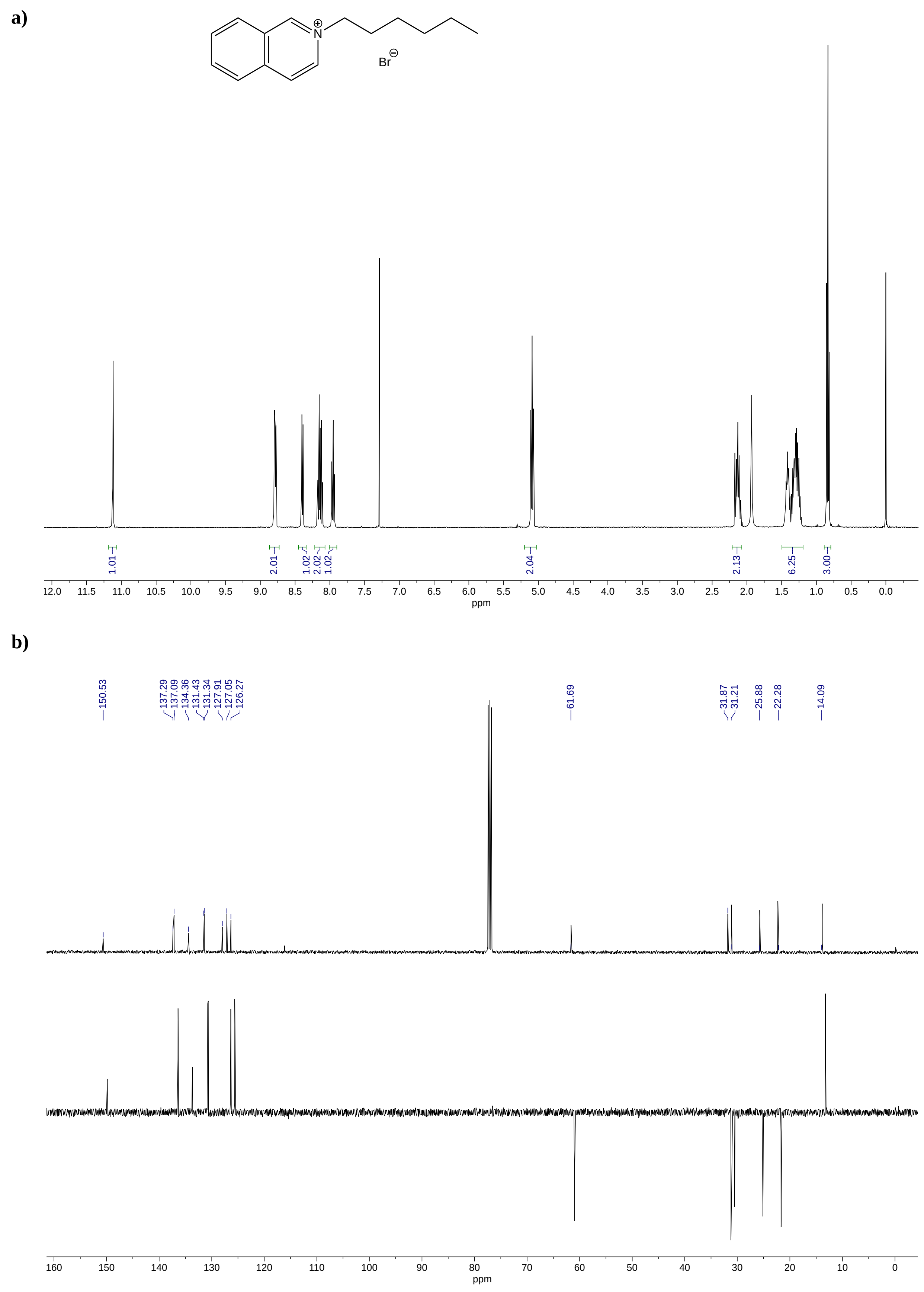

Figure S15. ^{1}H NMR **(a)**, ^{13}C NMR and DEPT135 **(b)** spectra of compound **3a**.

a)

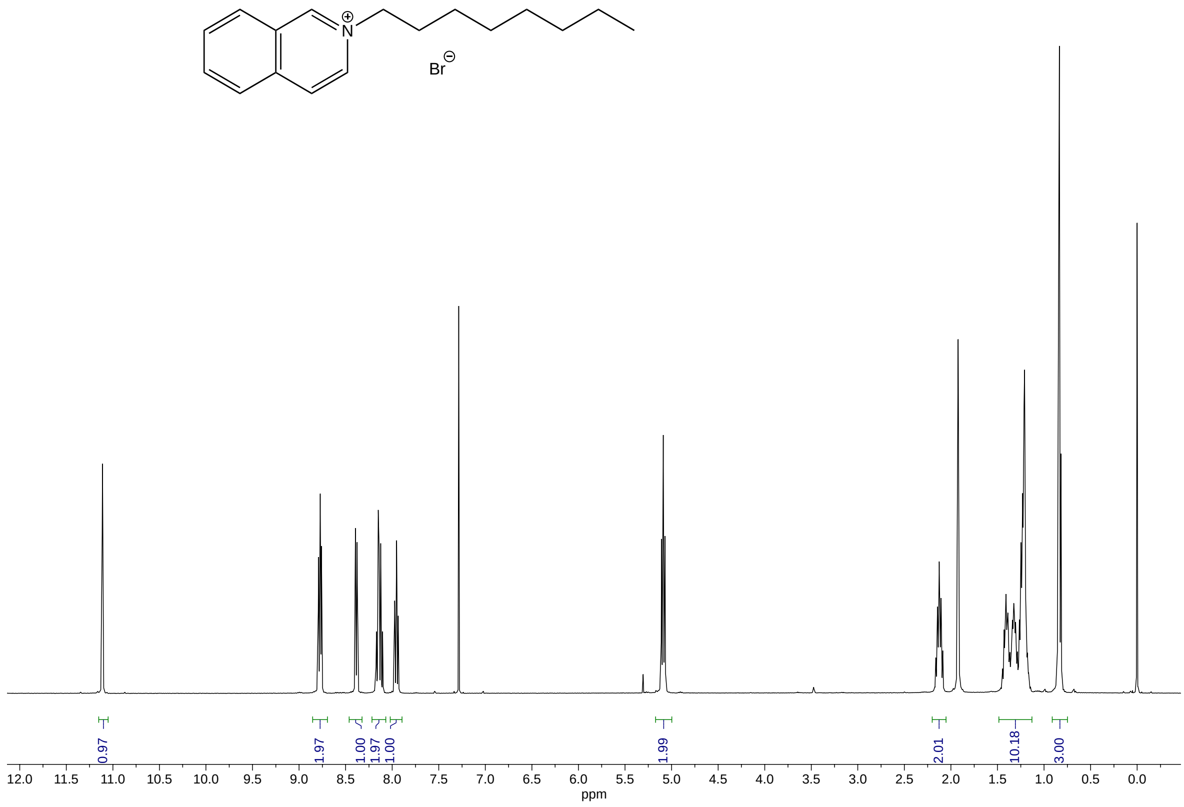

b)

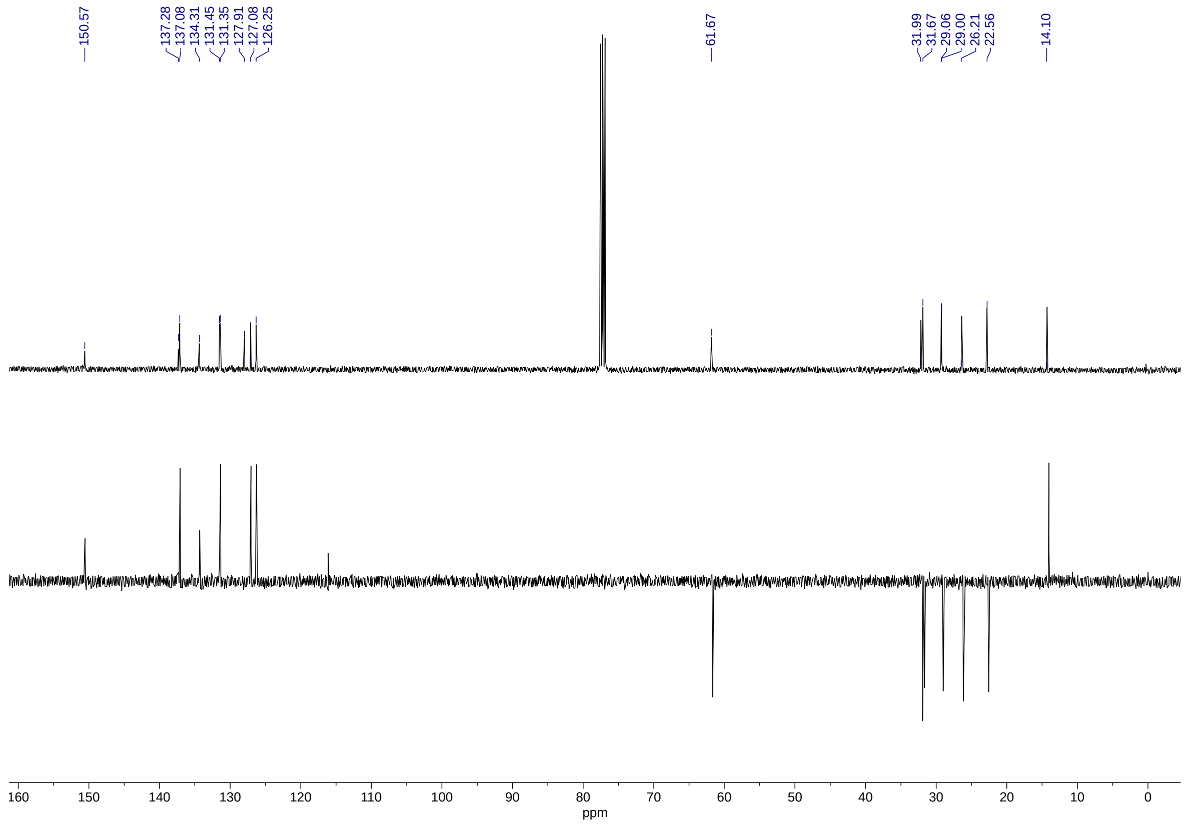

Figure S16. ^{1}H NMR **(a)**, ^{13}C NMR and DEPT135 **(b)** spectra of compound **3b**.

a)

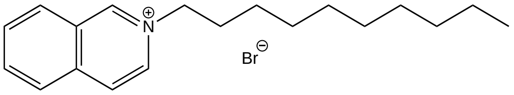

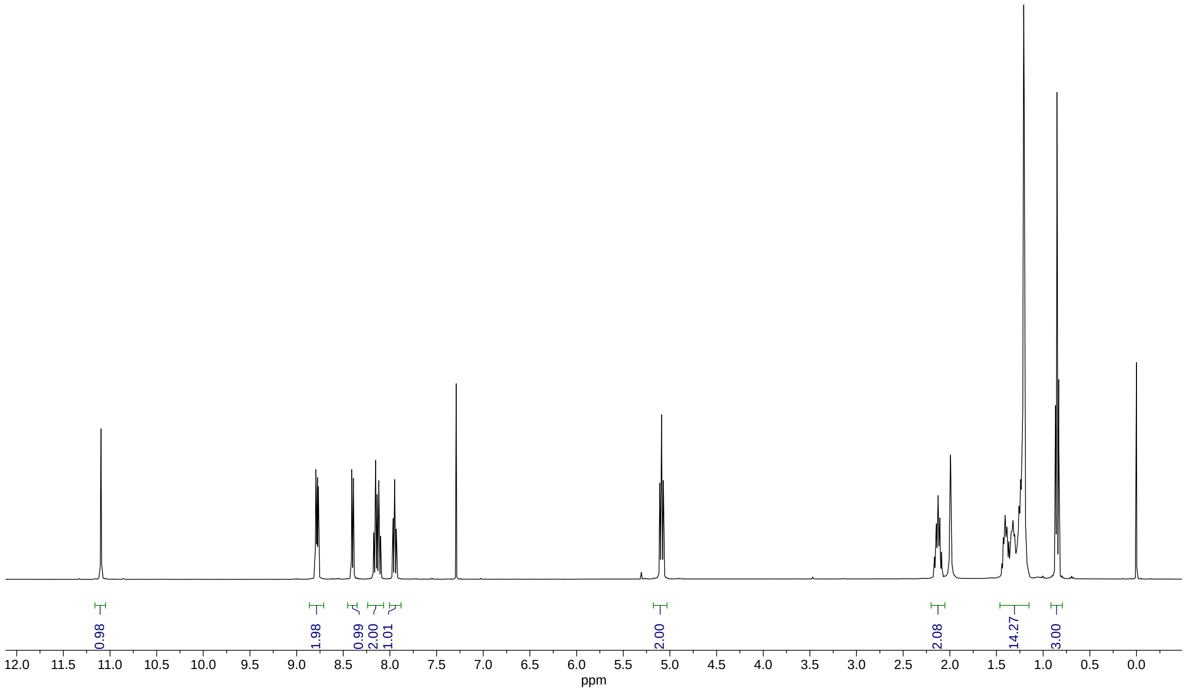

b)

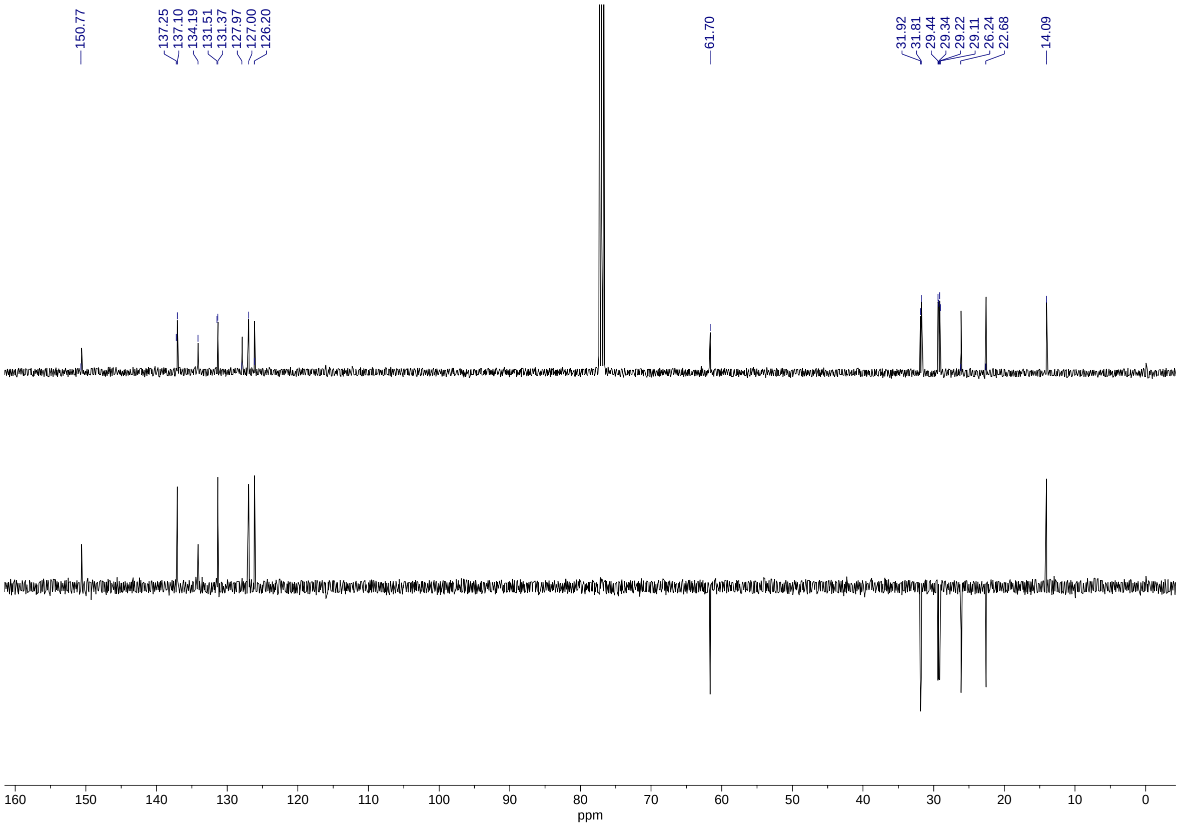

Figure S17. ^{1}H NMR **(a)**, ^{13}C NMR and DEPT135 **(b)** spectra of compound **3c**.

a)

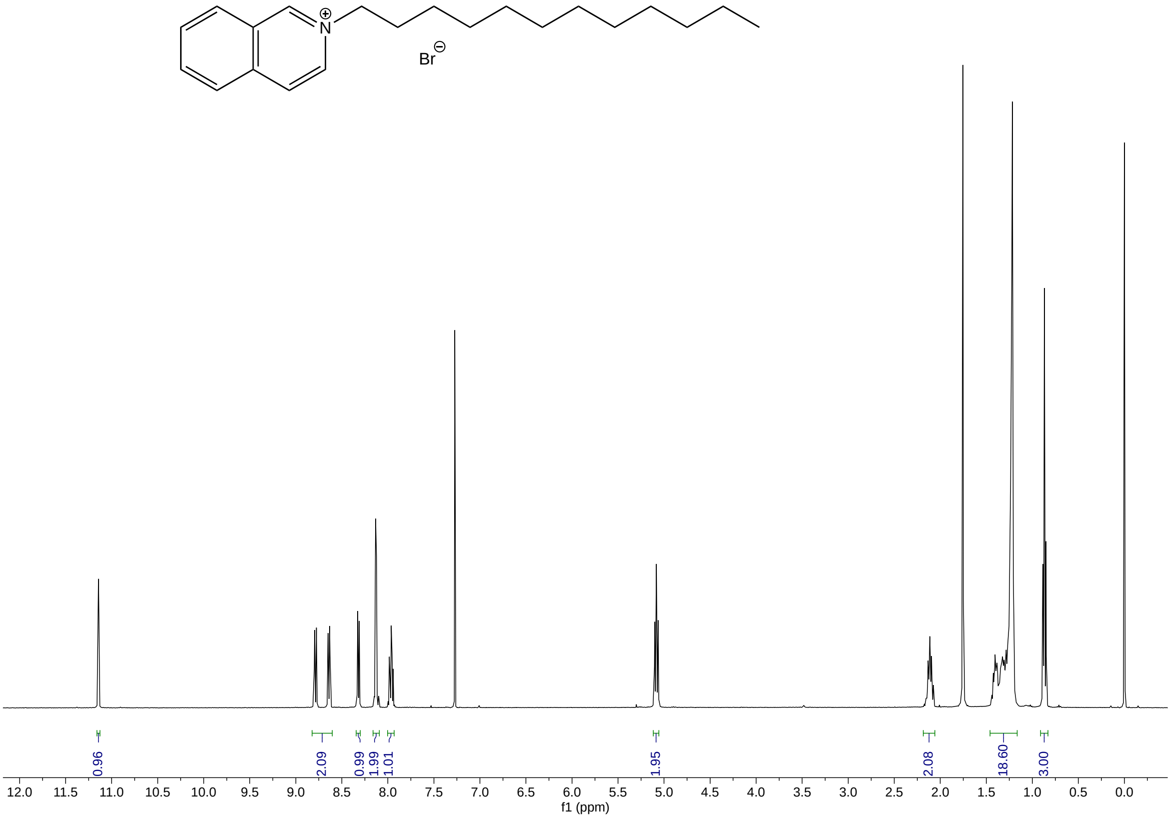

b)

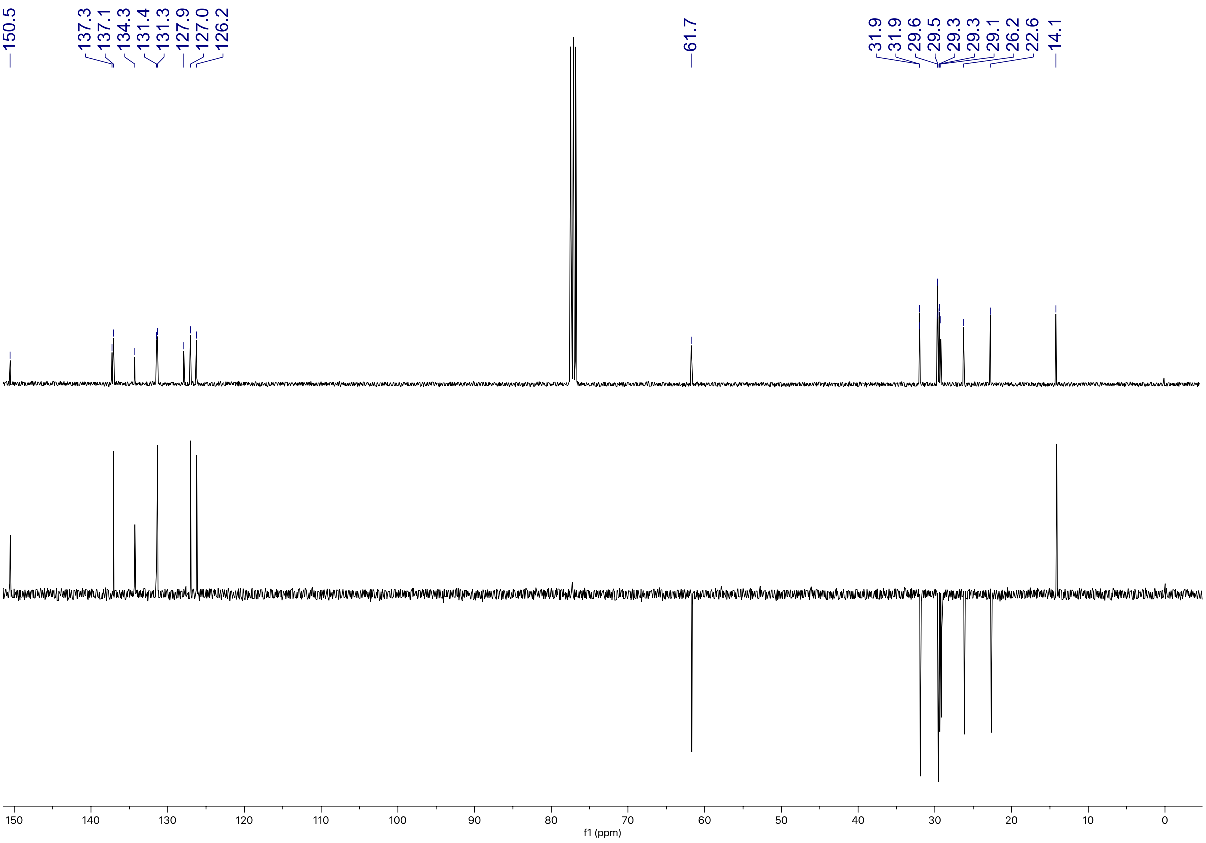

Figure S18. ^{1}H NMR **(a)**, ^{13}C NMR and DEPT135 **(b)** spectra of compound **3d**.

a)

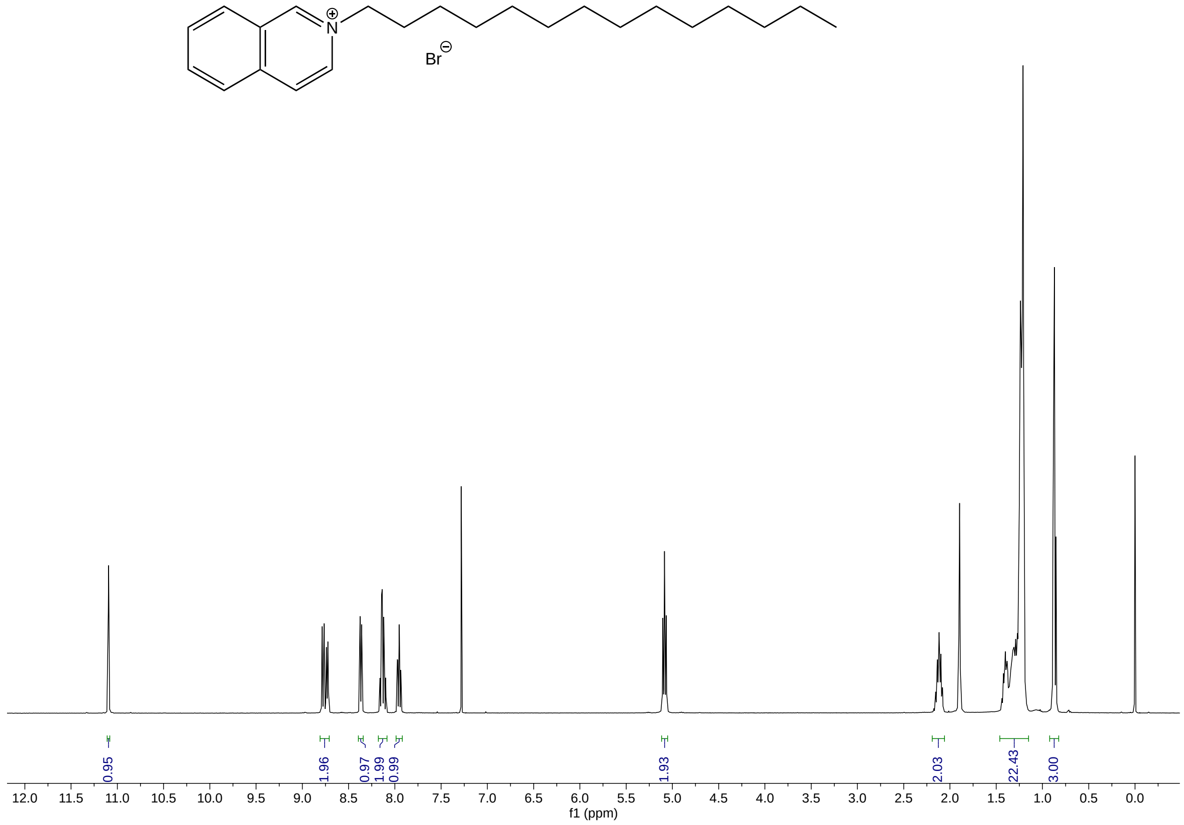

b)

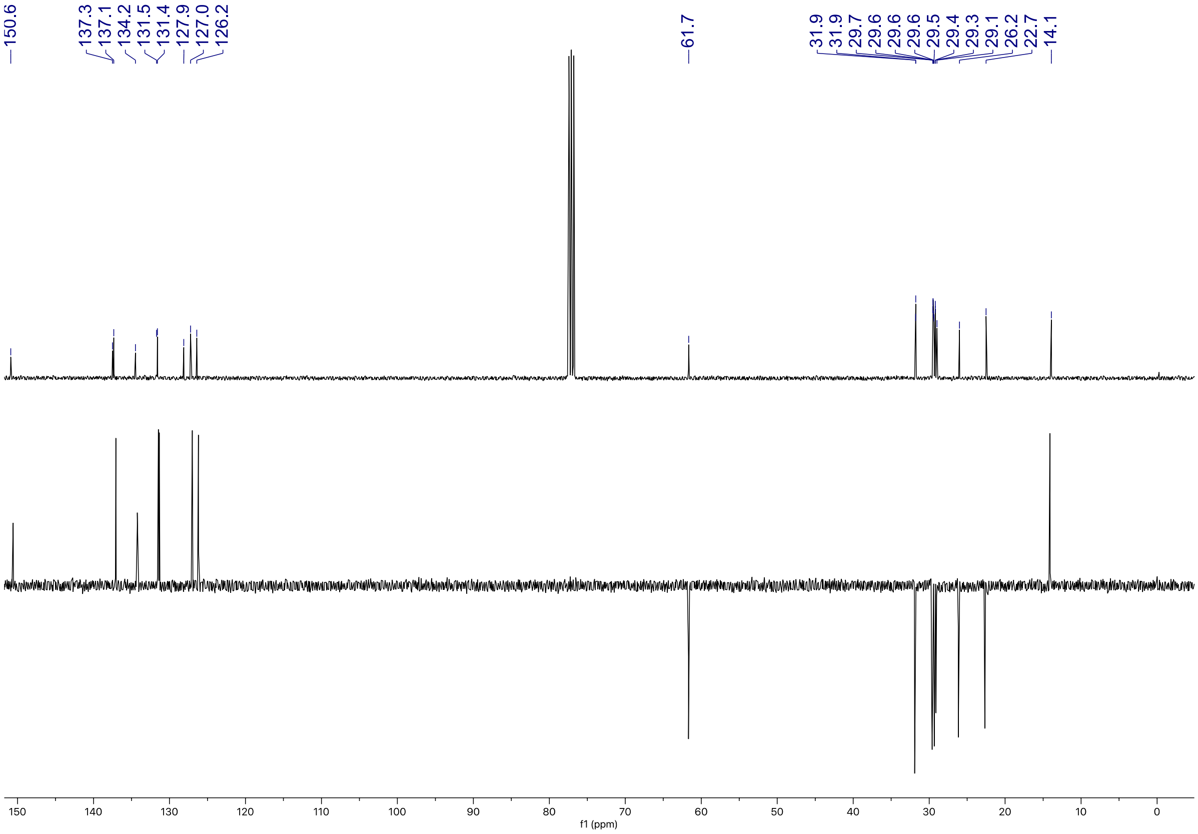

Figure S19. ^{1}H NMR **(a)**, ^{13}C NMR and DEPT135 **(b)** spectra of compound **3e**.

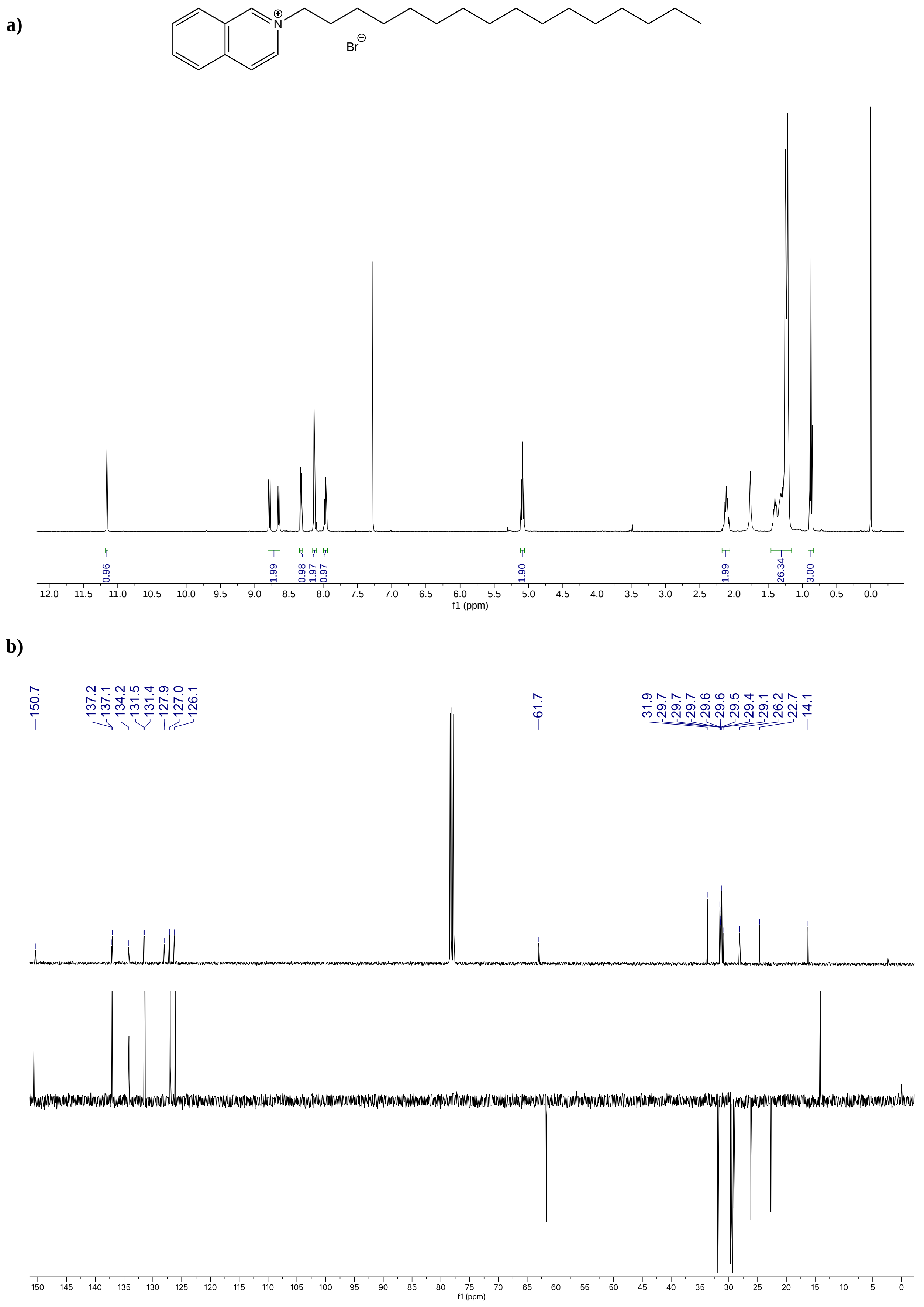

Figure S20. ^{1}H NMR **(a)**, ^{13}C NMR and DEPT135 **(b)** spectra of compound **3f**.

a)

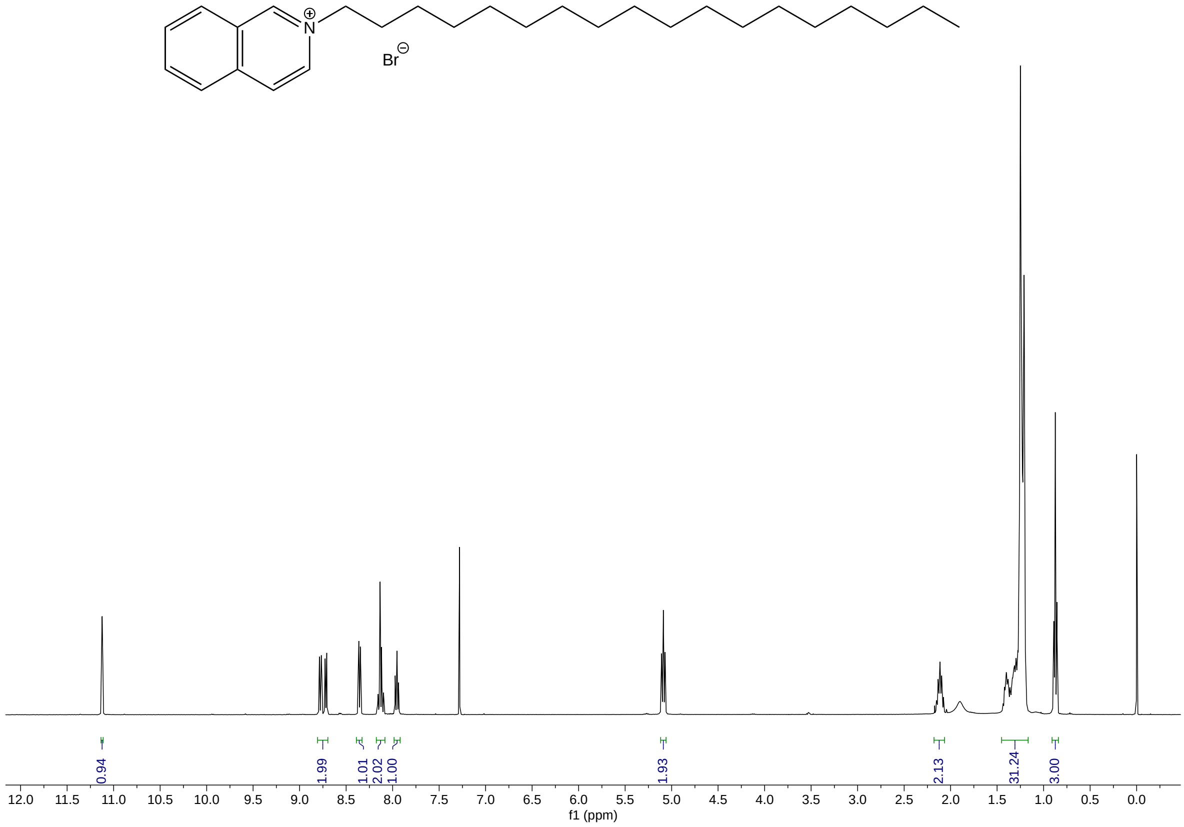

b)

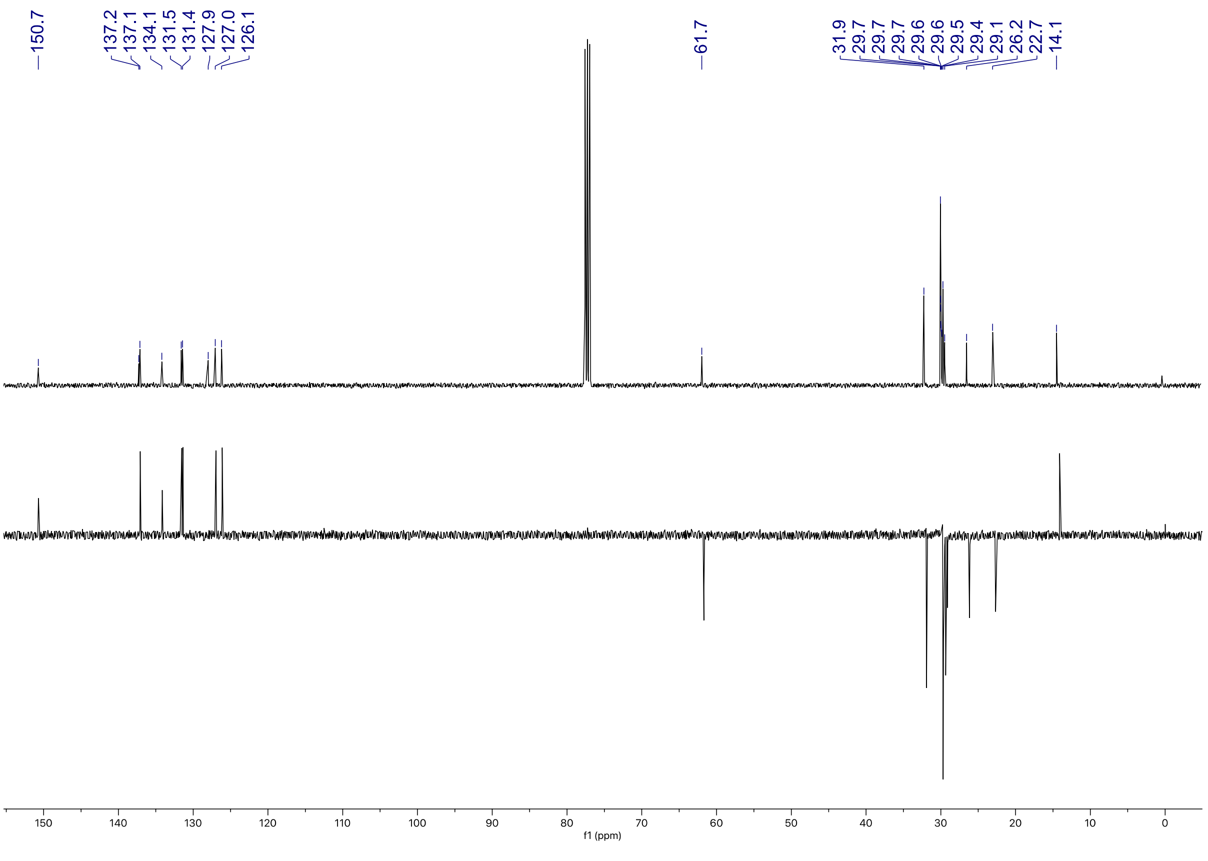

Figure S21. ^{1}H NMR **(a)**, ^{13}C NMR and DEPT135 **(b)** spectra of compound **3g**.

a)

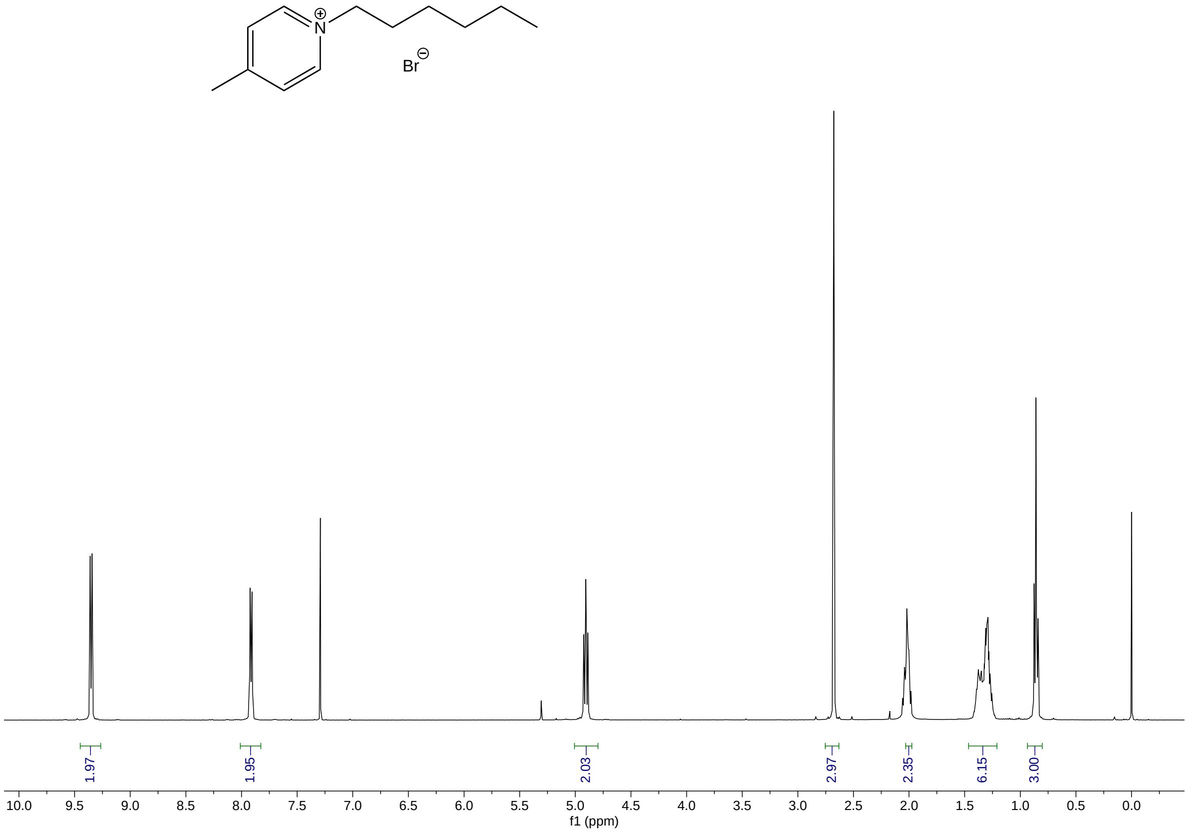

b)

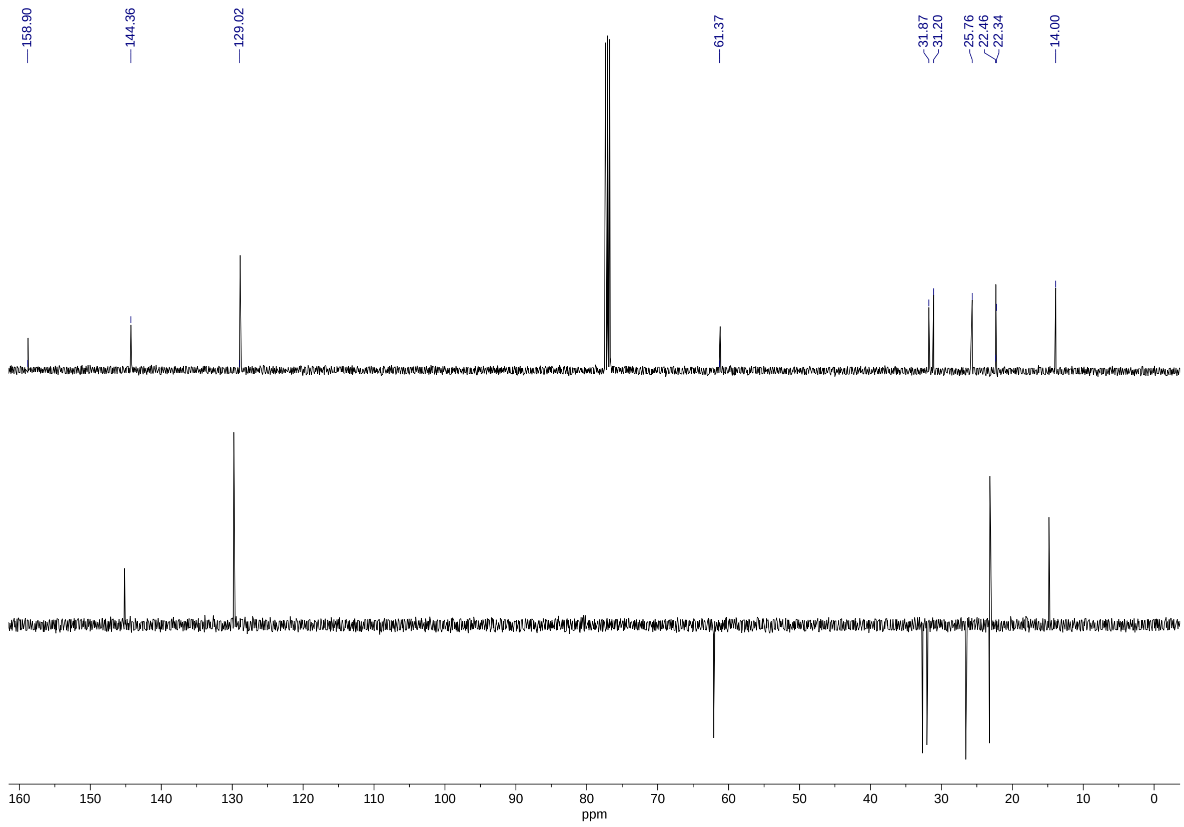

Figure S22. ^{1}H NMR **(a)**, ^{13}C NMR and DEPT135 **(b)** spectra of compound **4a**.

a)

b)

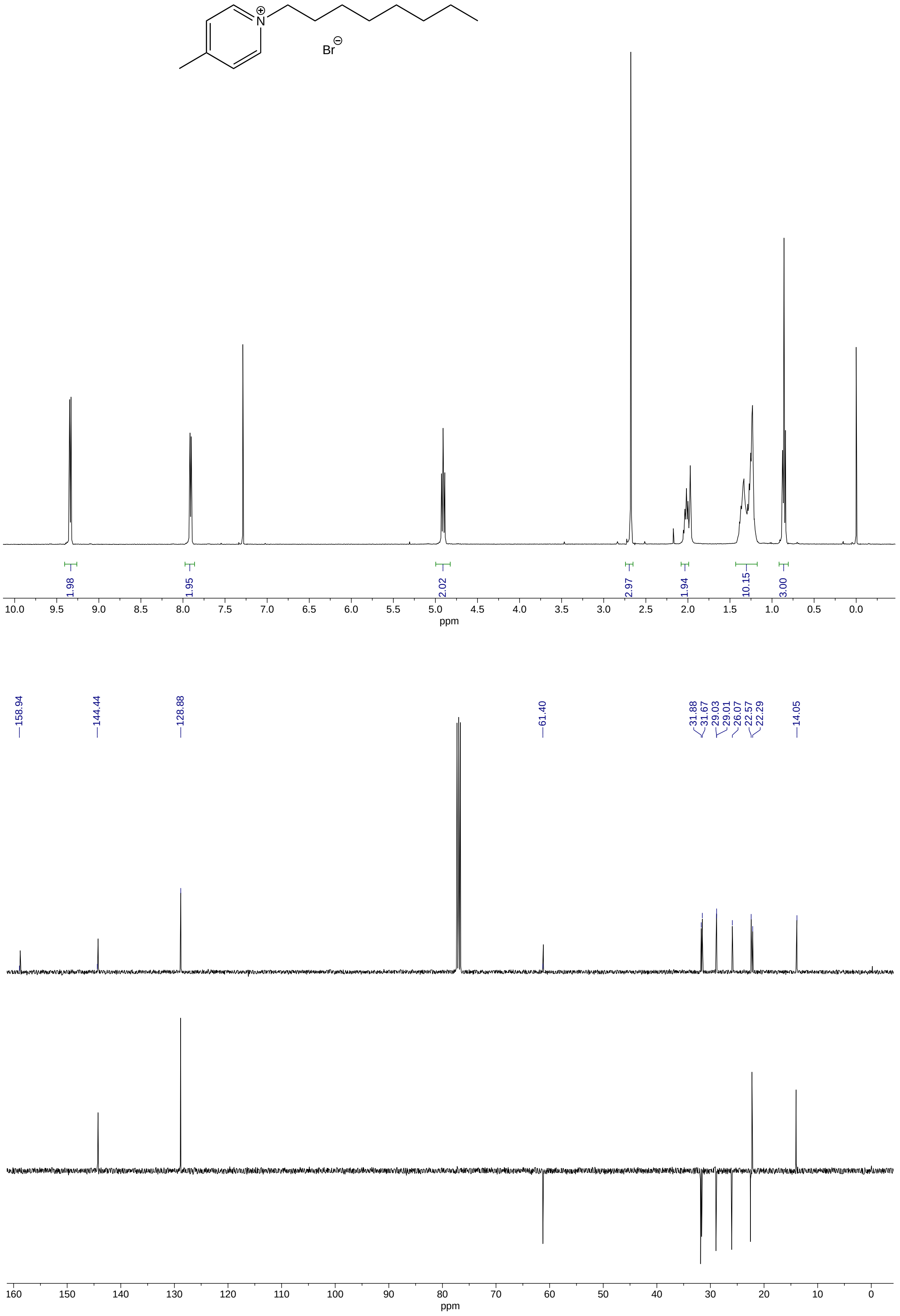

Figure S23. ^{1}H NMR **(a)**, ^{13}C NMR and DEPT135 **(b)** spectra of compound **4b**.

a)

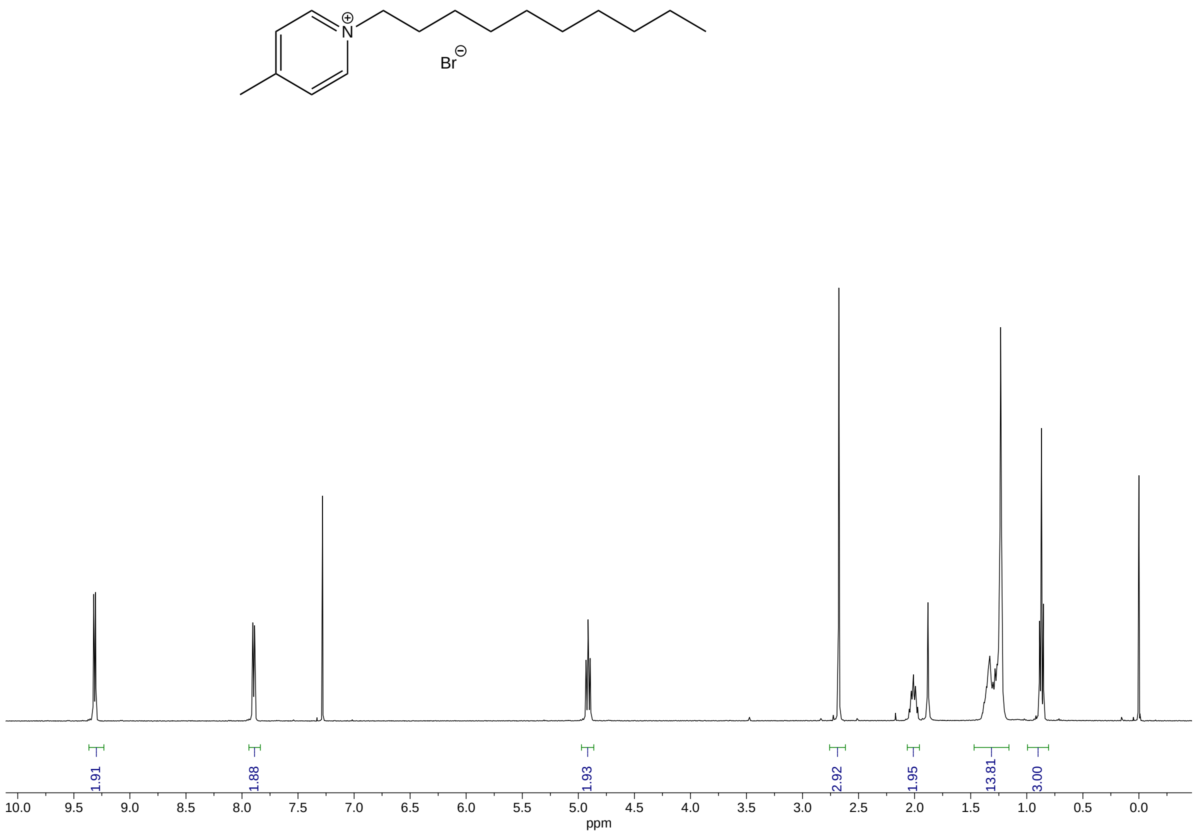

b)

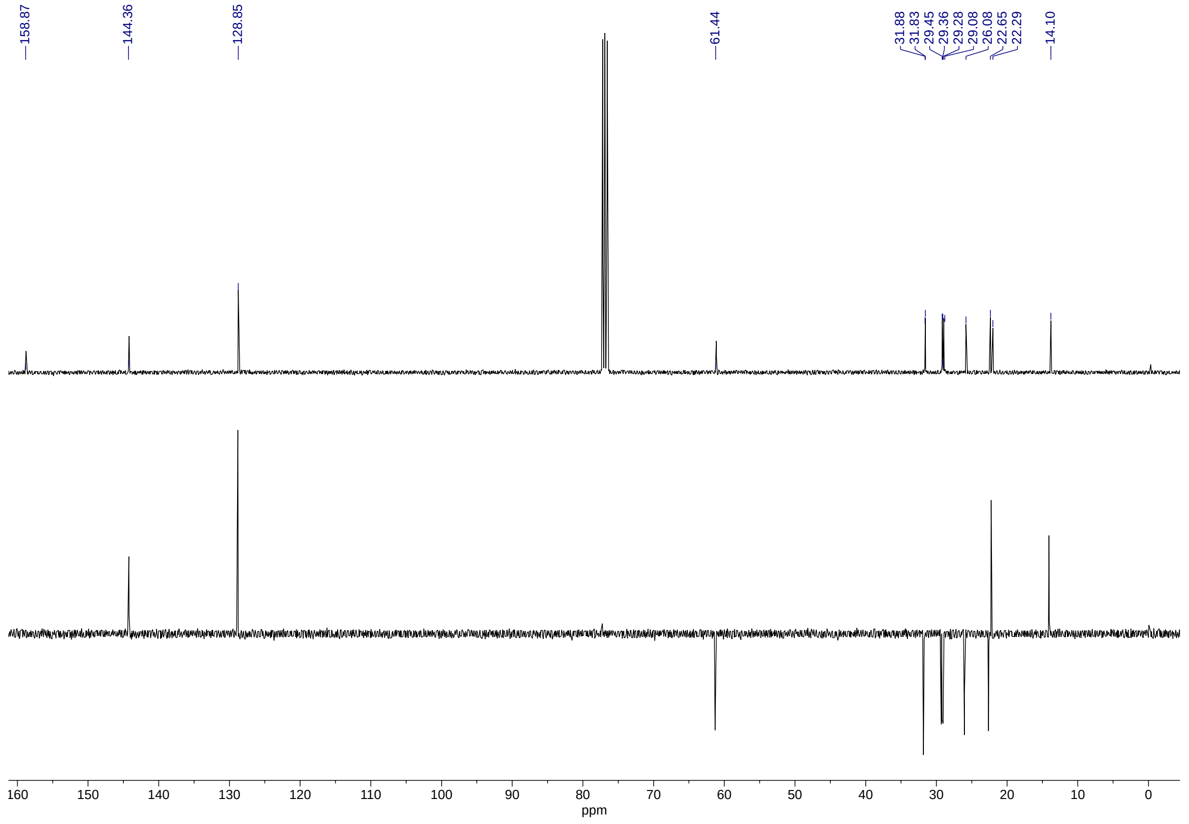

Figure S24. ^{1}H NMR **(a)**, ^{13}C NMR and DEPT135 **(b)** spectra of compound **4c**.

a)

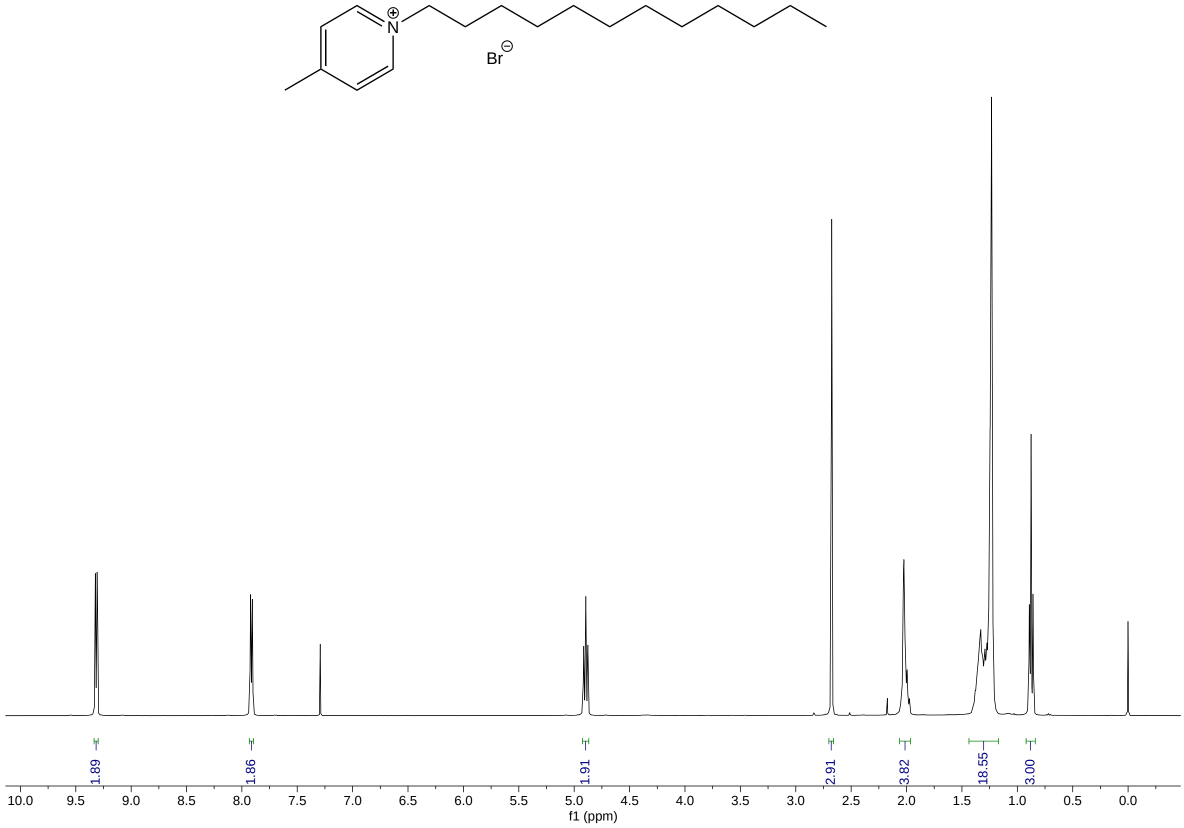

b)

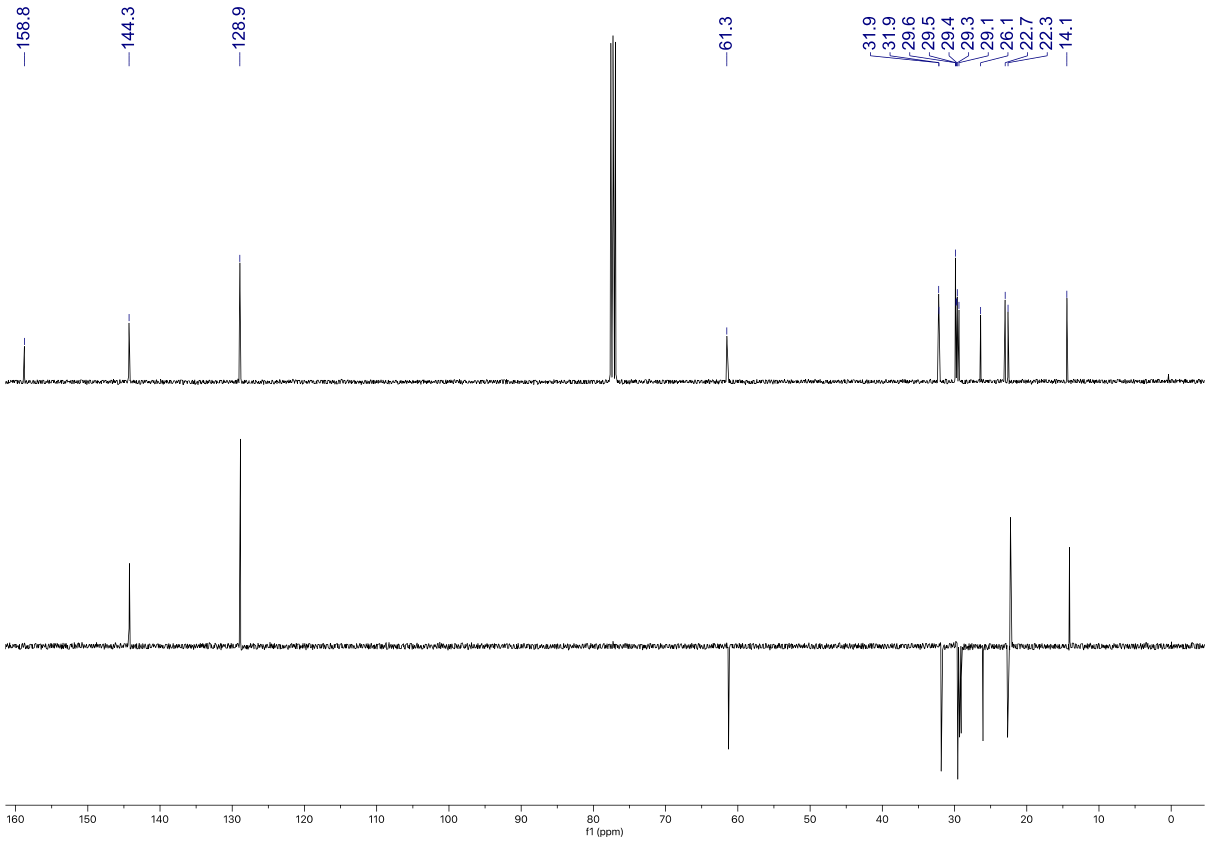

Figure S25. ^{1}H NMR **(a)**, ^{13}C NMR and DEPT135 **(b)** spectra of compound **4d**.

a)

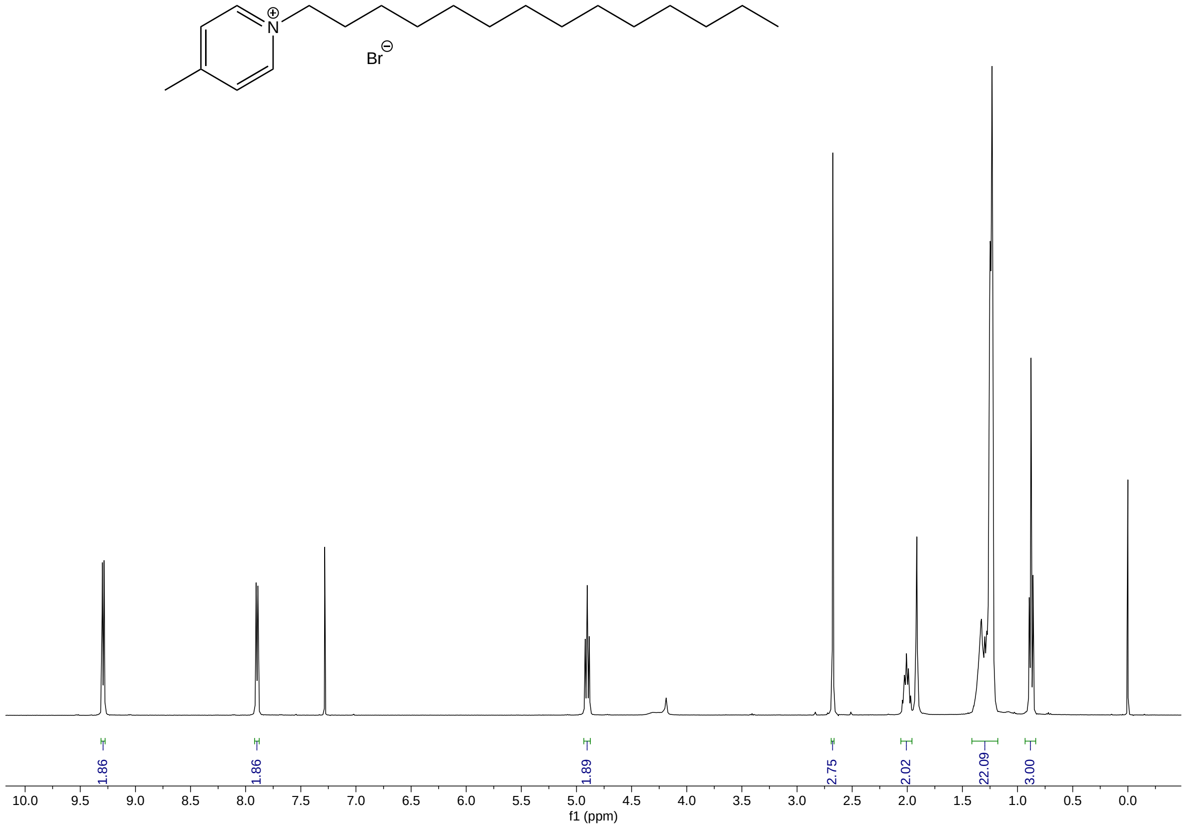

b)

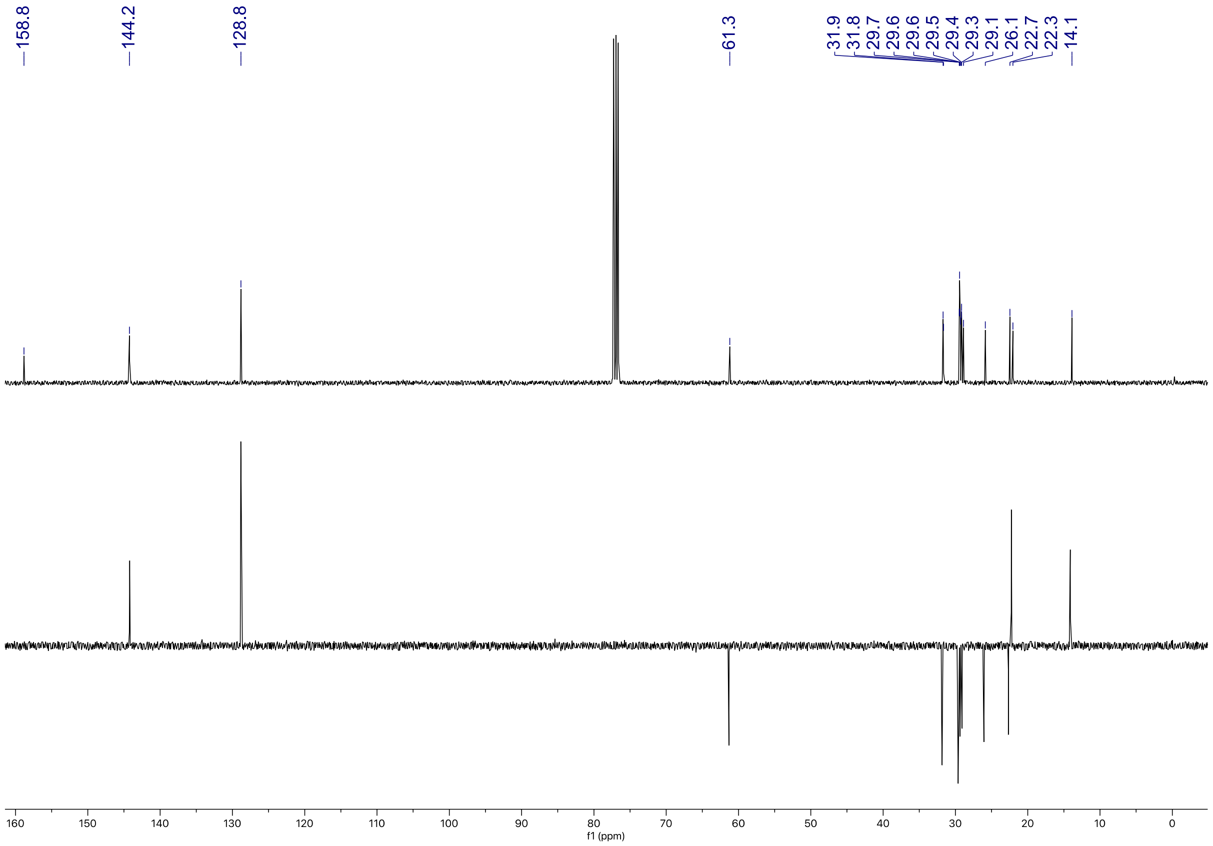

Figure S26. ^{1}H NMR **(a)**, ^{13}C NMR and DEPT135 **(b)** spectra of compound **4e**.

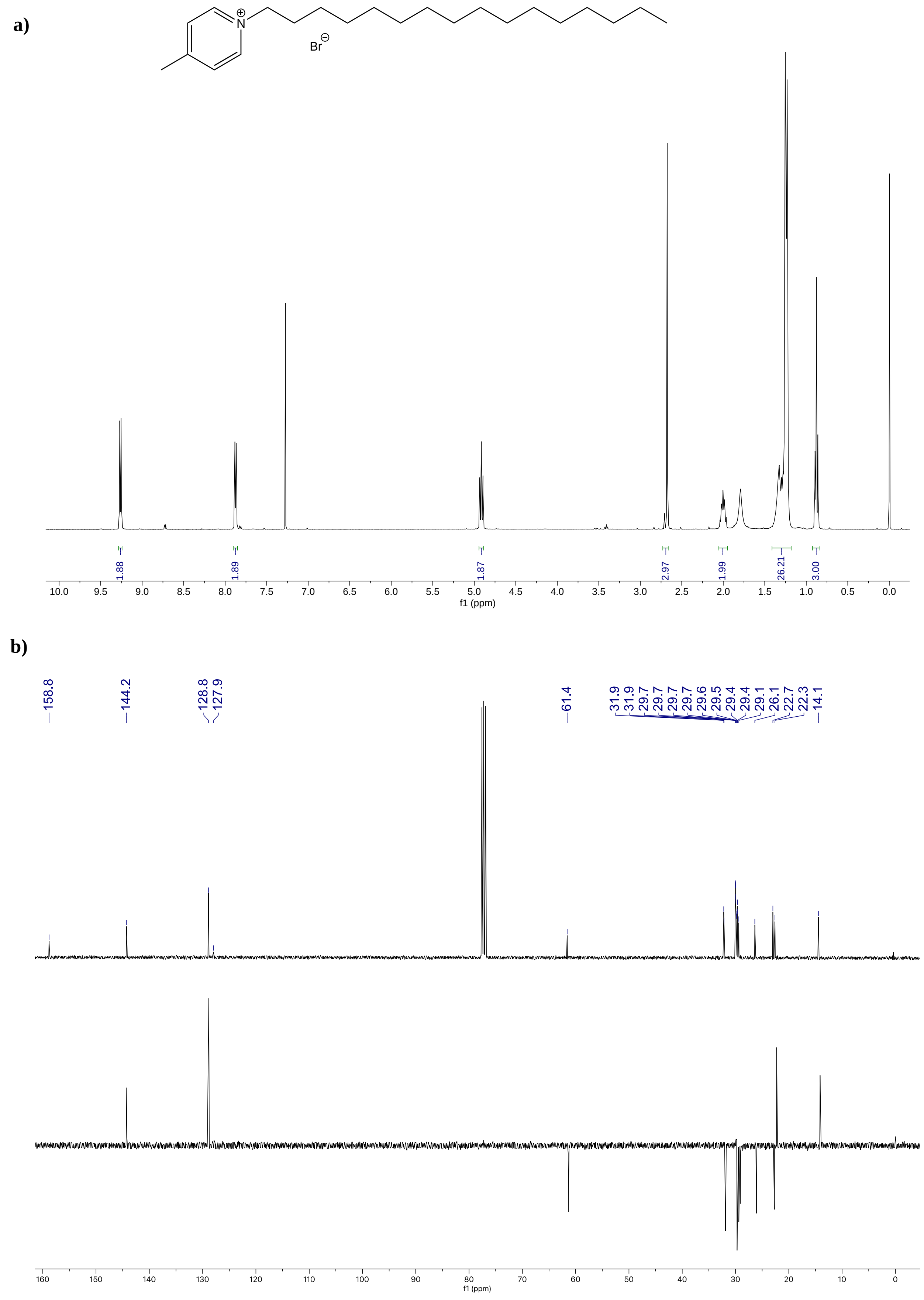

Figure S27. ^{1}H NMR **(a)**, ^{13}C NMR and DEPT135 **(b)** spectra of compound **4f**.

a)

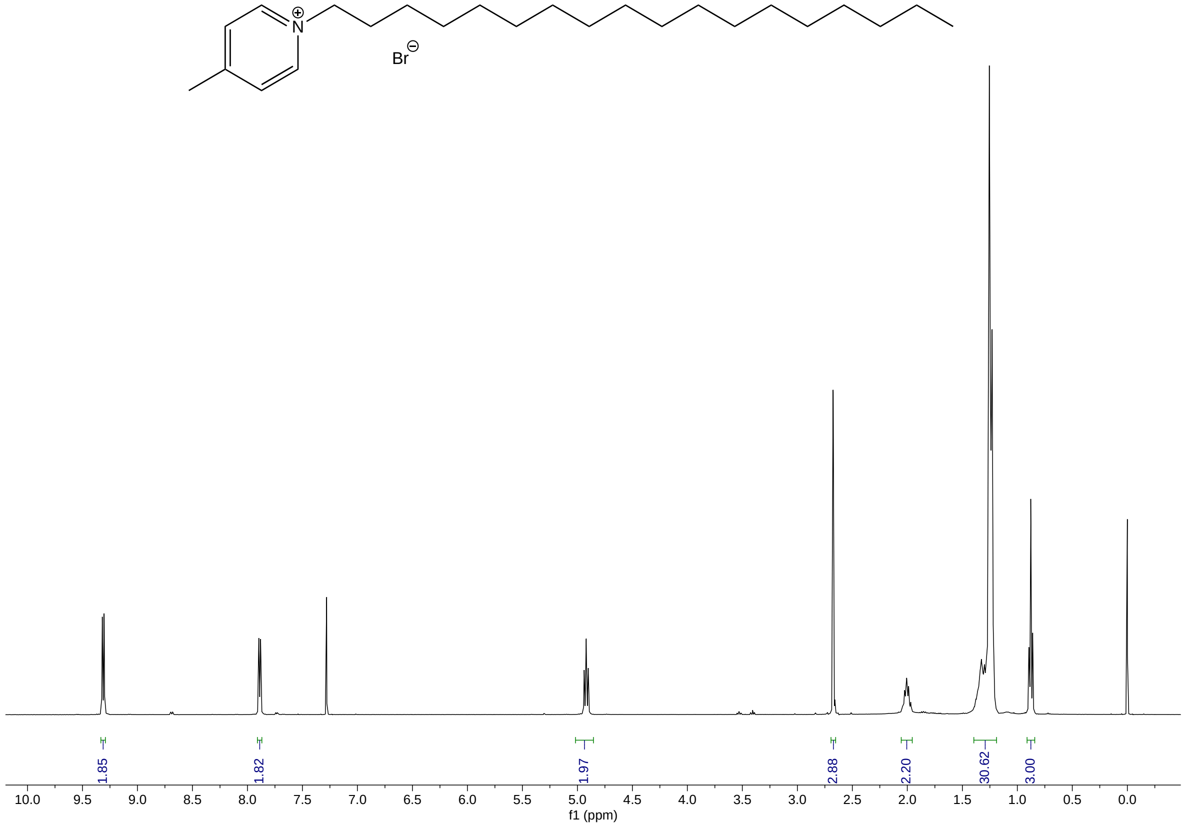

b)

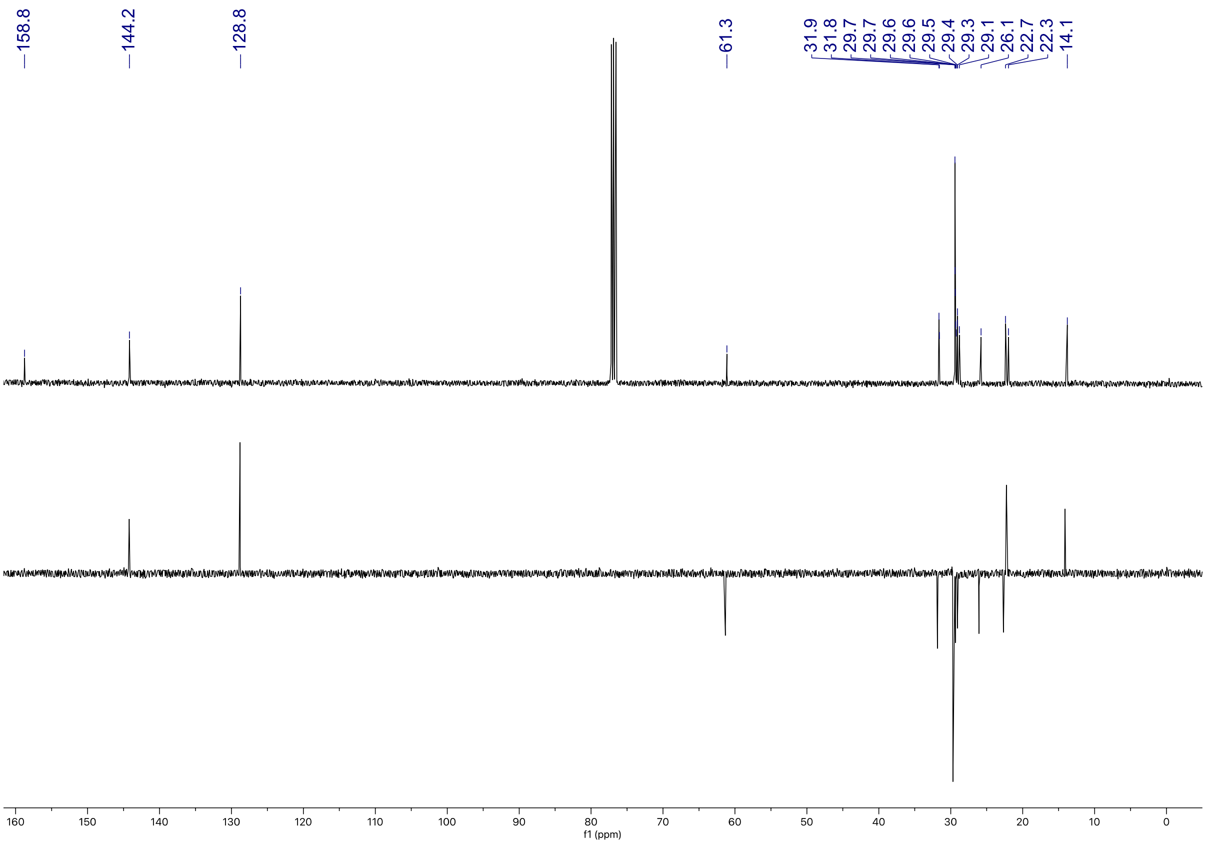

Figure S28. ^{1}H NMR **(a)**, ^{13}C NMR and DEPT135 **(b)** spectra of compound **4g**.

a)

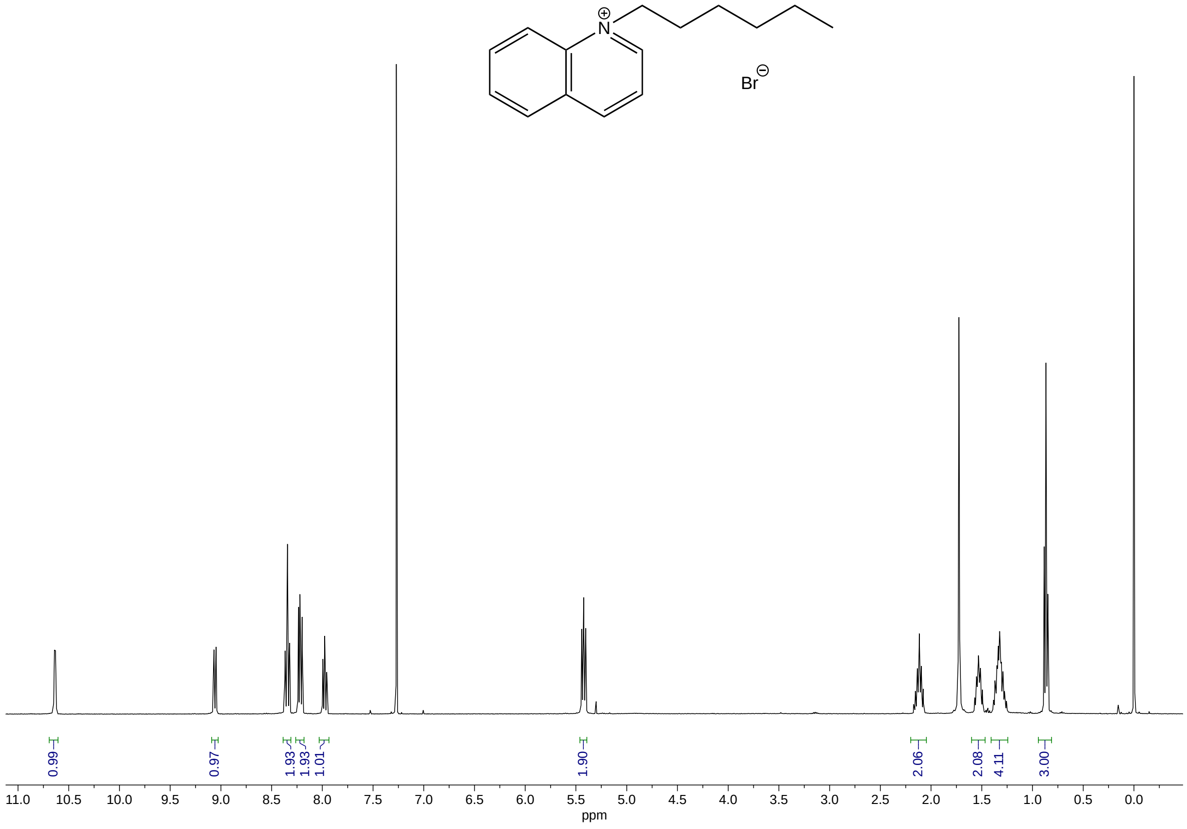

b)

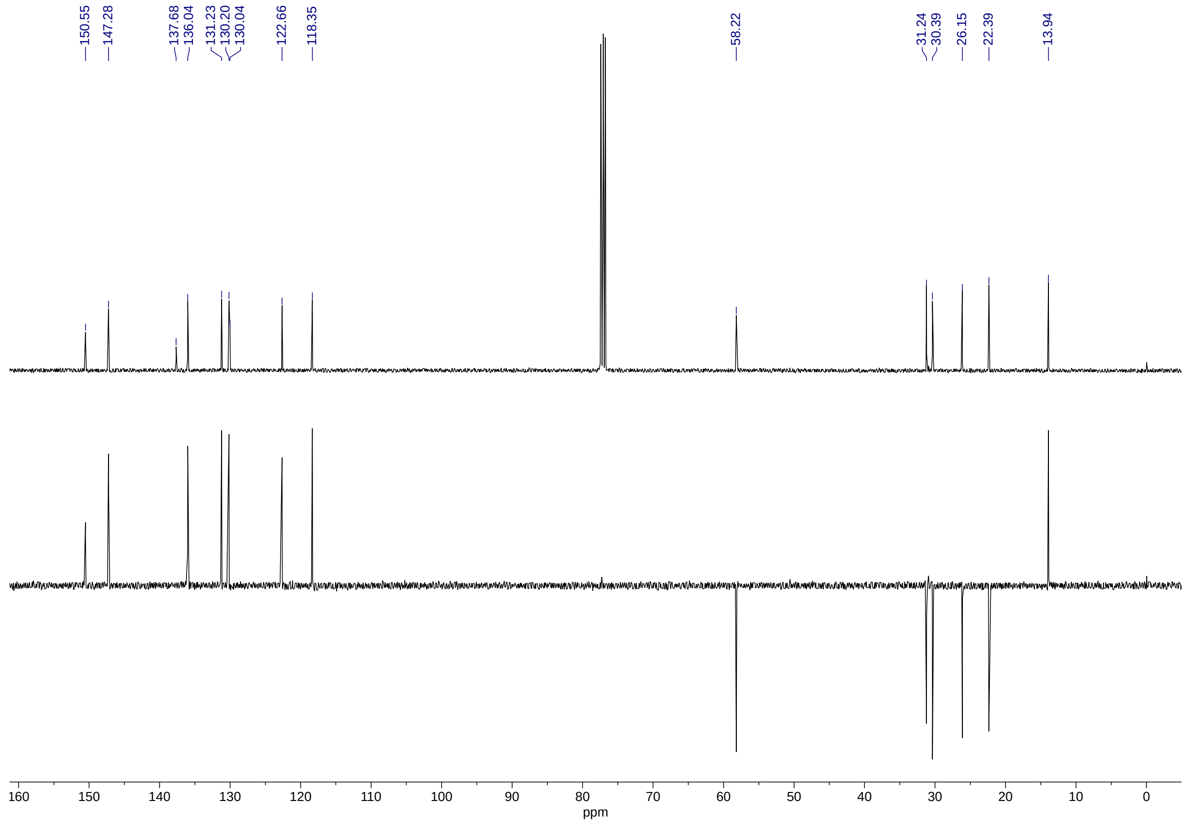

Figure S29. ^{1}H NMR **(a)**, ^{13}C NMR and DEPT135 **(b)** spectra of compound **5a**.

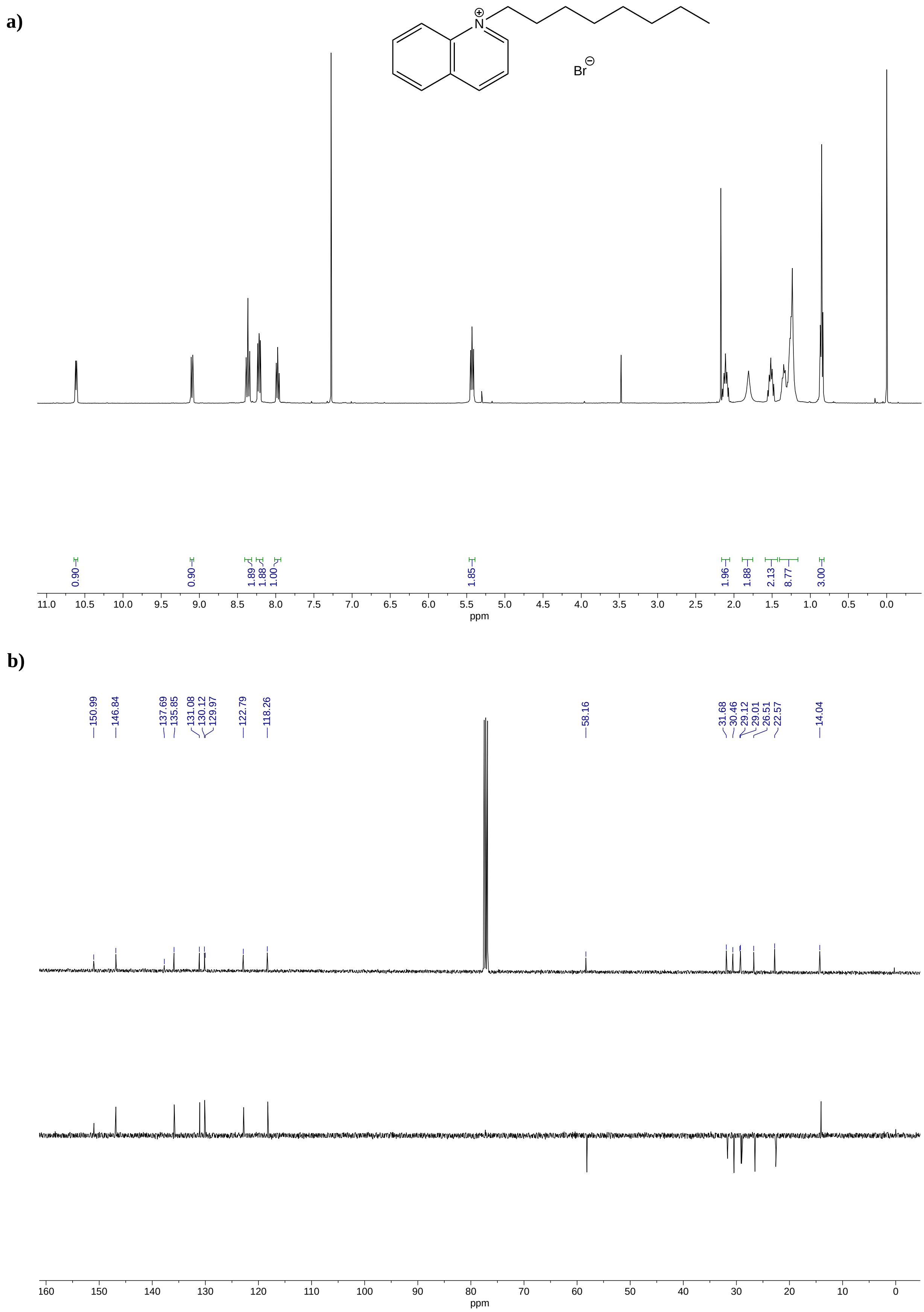

Figure S30. ^{1}H NMR **(a)**, ^{13}C NMR and DEPT135 **(b)** spectra of compound **5b**.

a)

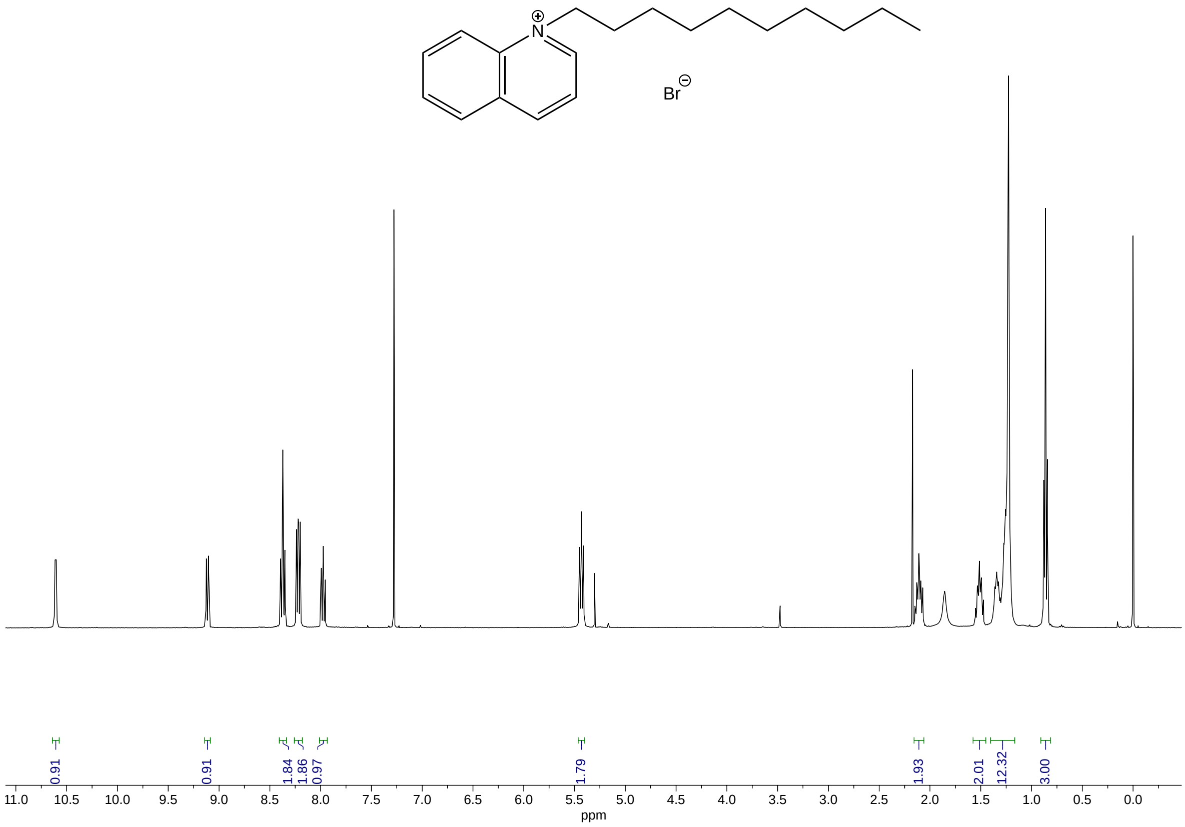

b)

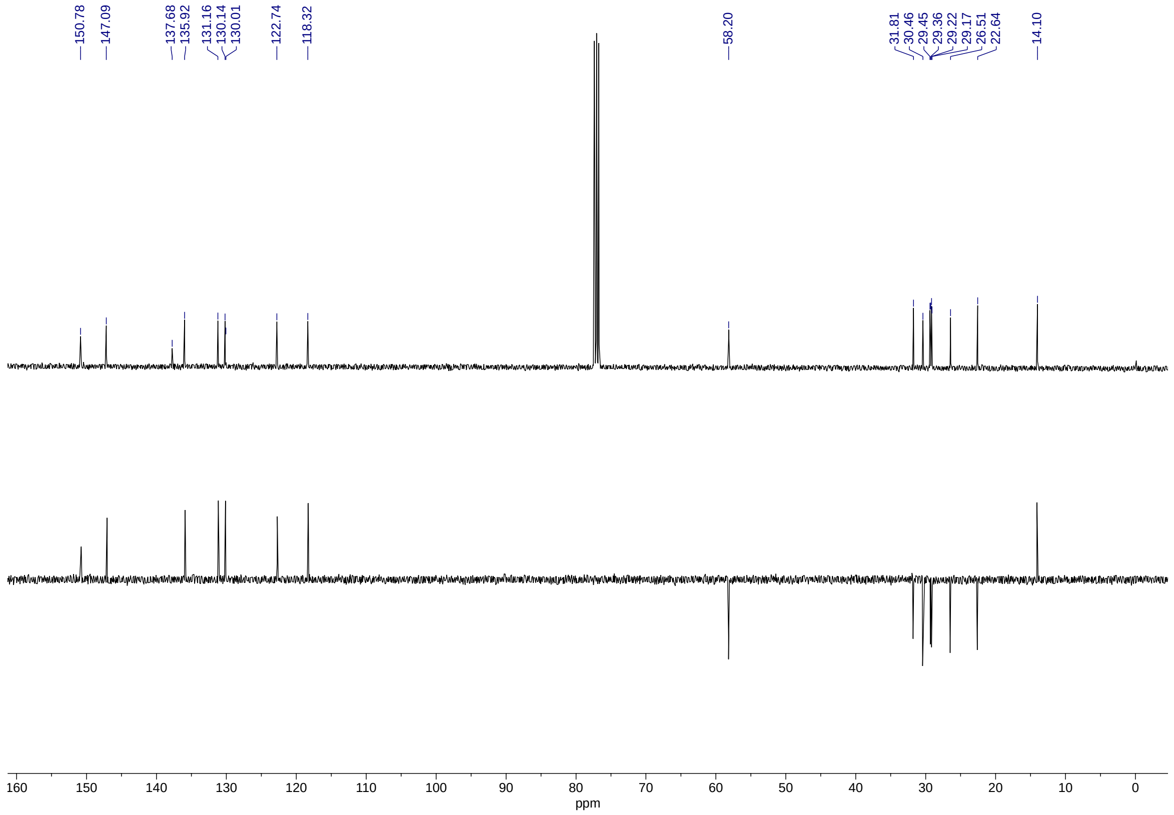

Figure S31. ^{1}H NMR **(a)**, ^{13}C NMR and DEPT135 **(b)** spectra of compound **5c**.

a)

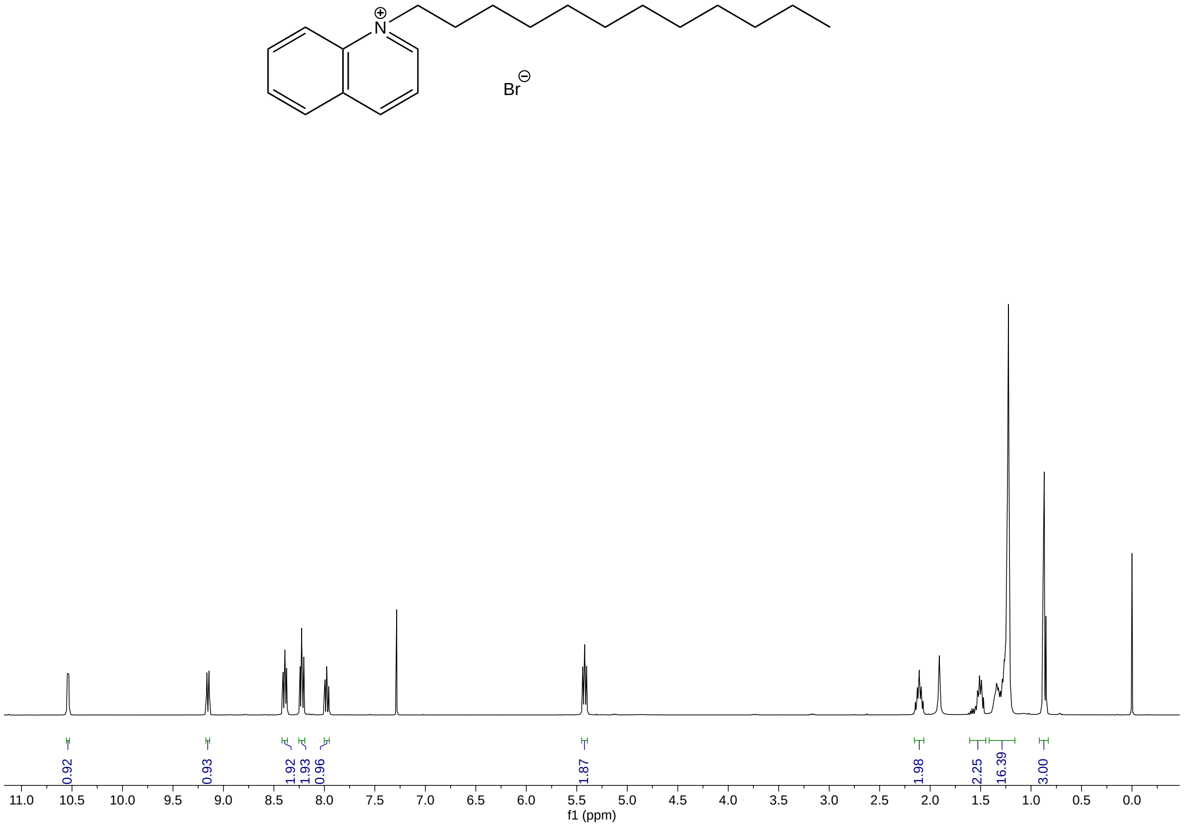

b)

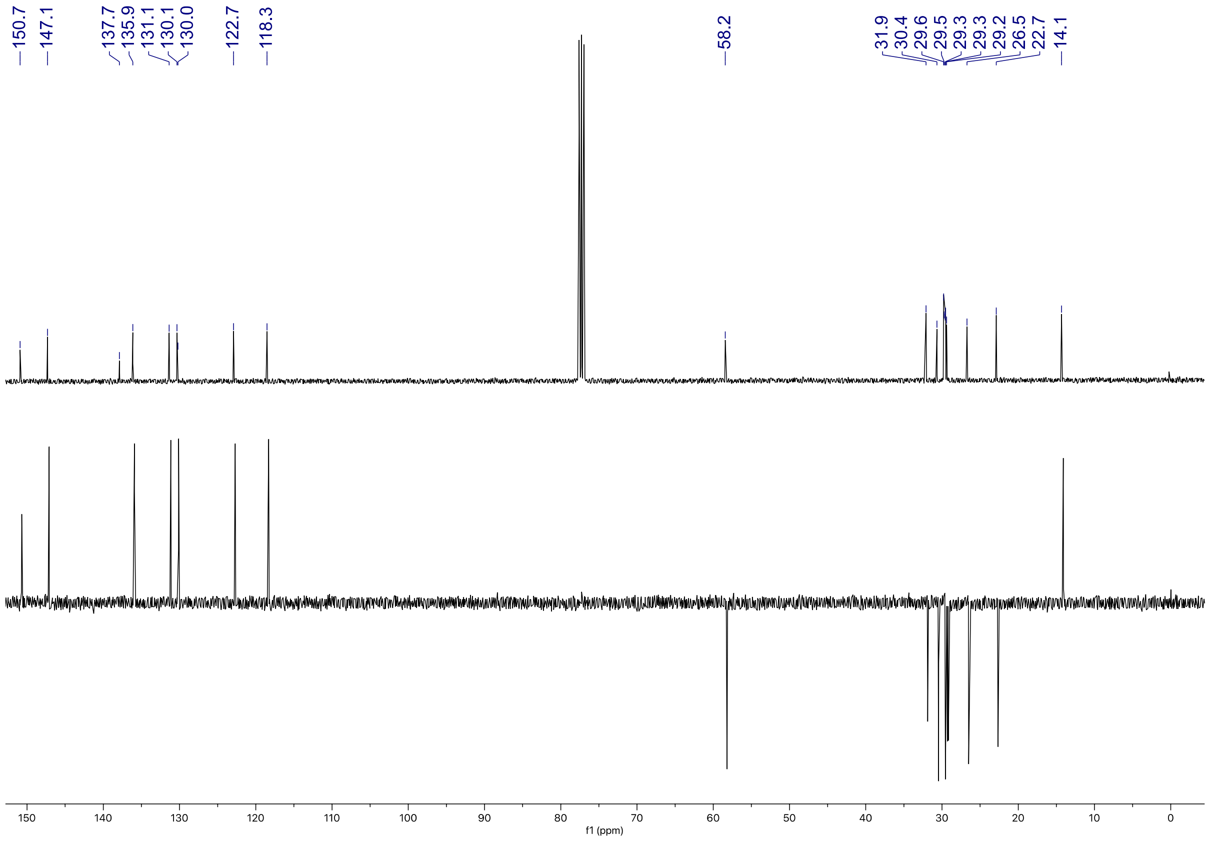

Figure S32. ^{1}H NMR **(a)**, ^{13}C NMR and DEPT135 **(b)** spectra of compound **5d**.

a)

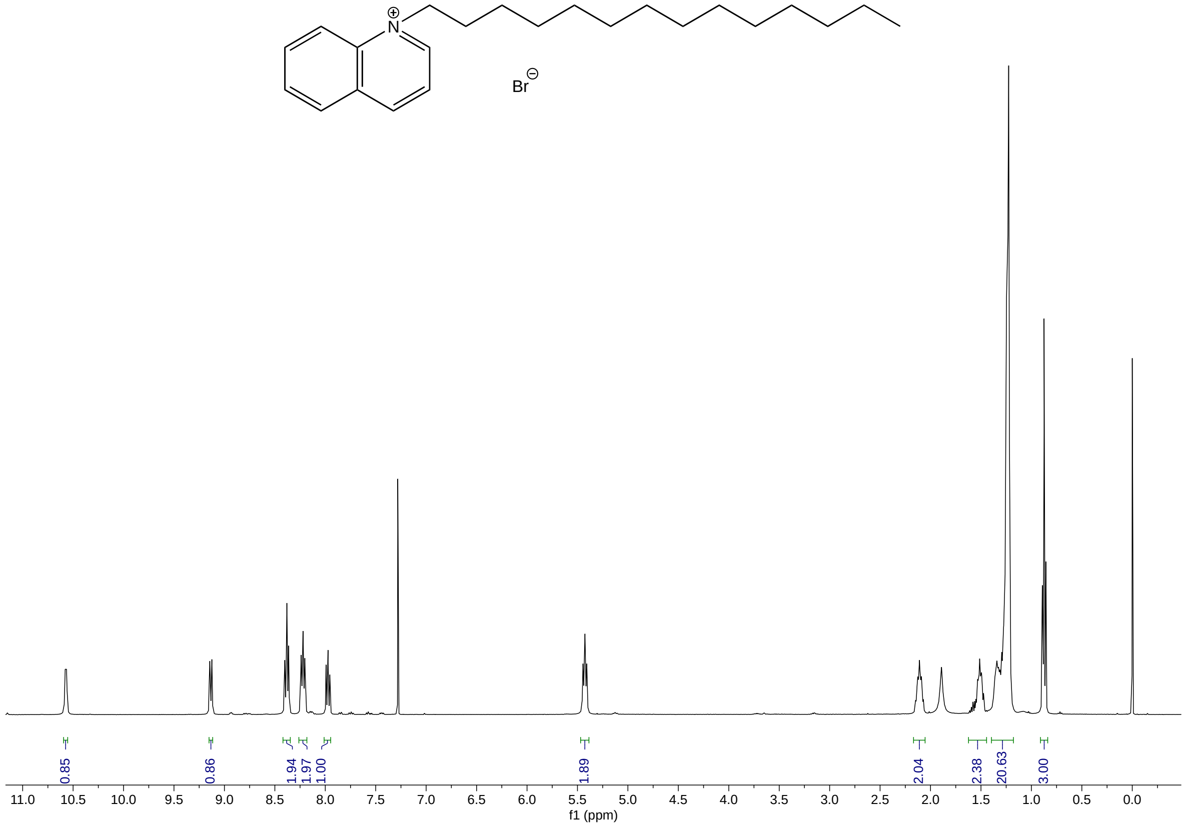

b)

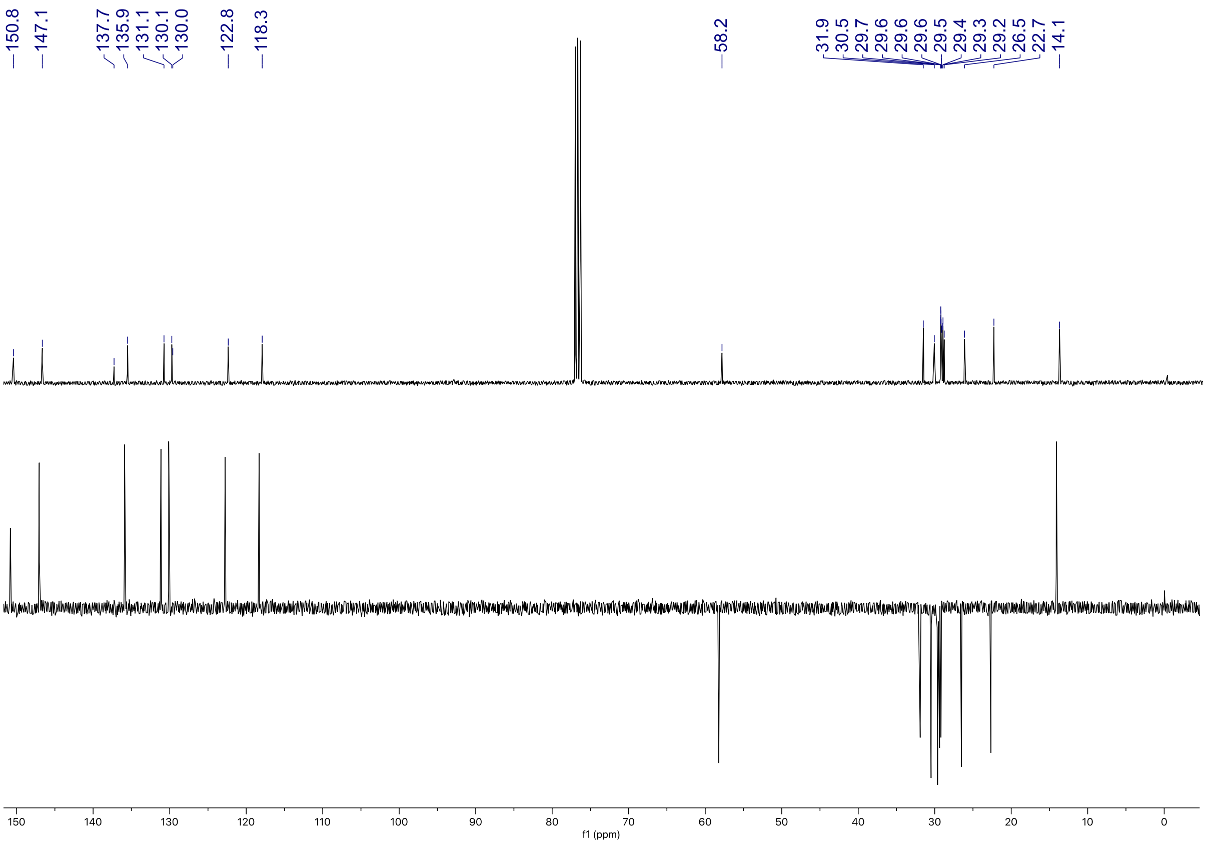

Figure S33. ^{1}H NMR **(a)**, ^{13}C NMR and DEPT135 **(b)** spectra of compound **5e**.

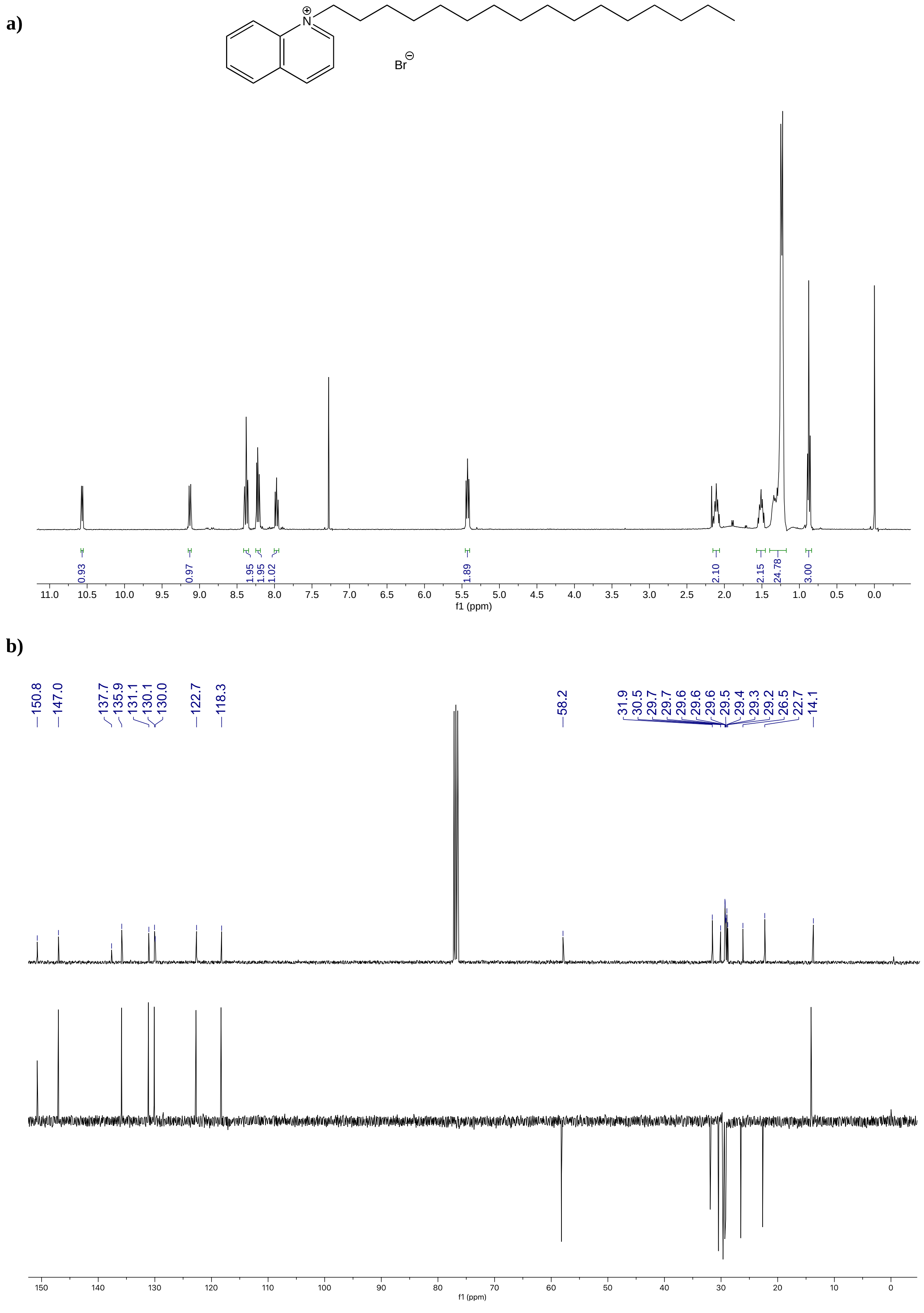

Figure S34. ^{1}H NMR **(a)**, ^{13}C NMR and DEPT135 **(b)** spectra of compound **5f**.

a)

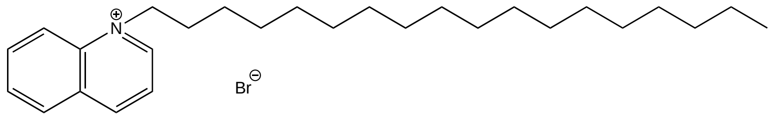

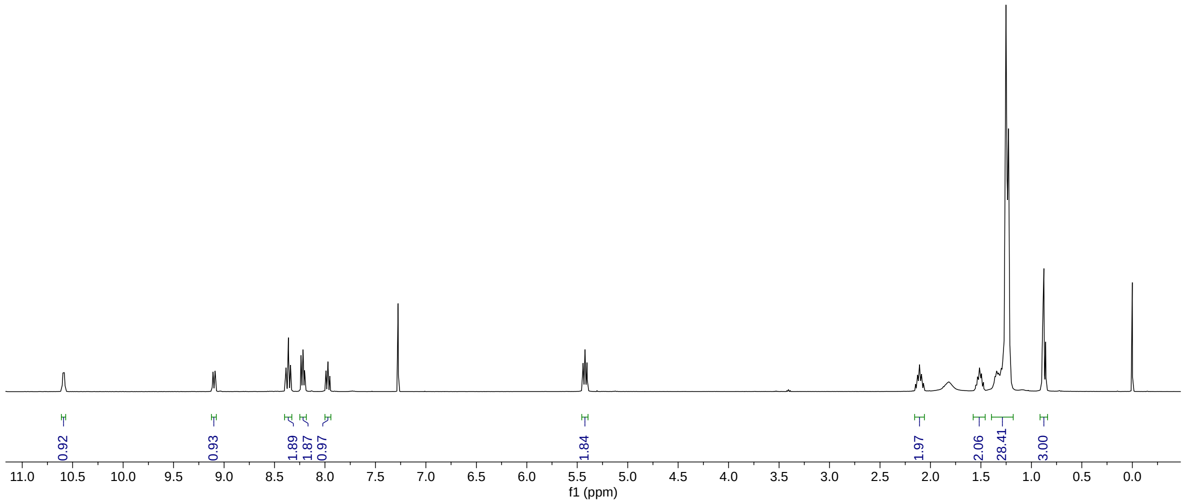

b)

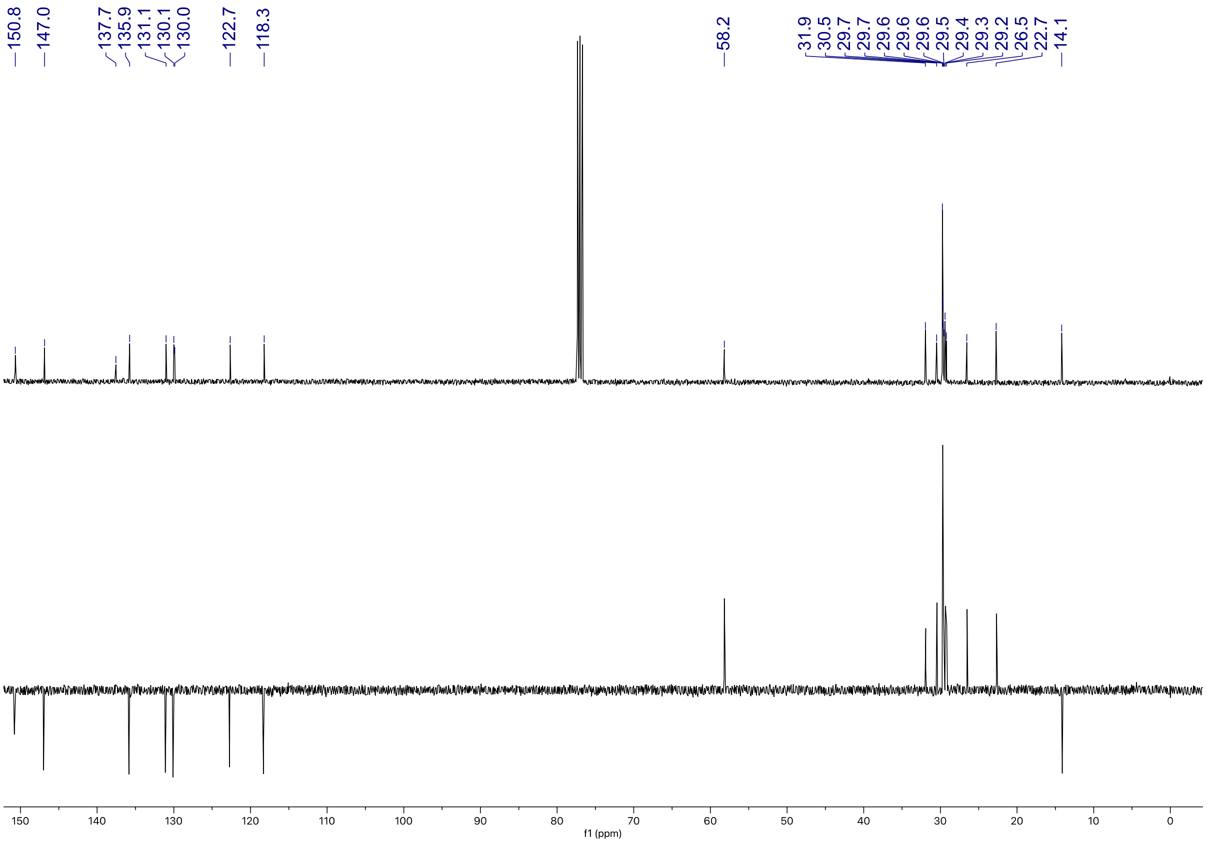

Figure S35. ^{1}H NMR **(a)**, ^{13}C NMR and DEPT135 **(b)** spectra of compound **5g**.

a)

b)

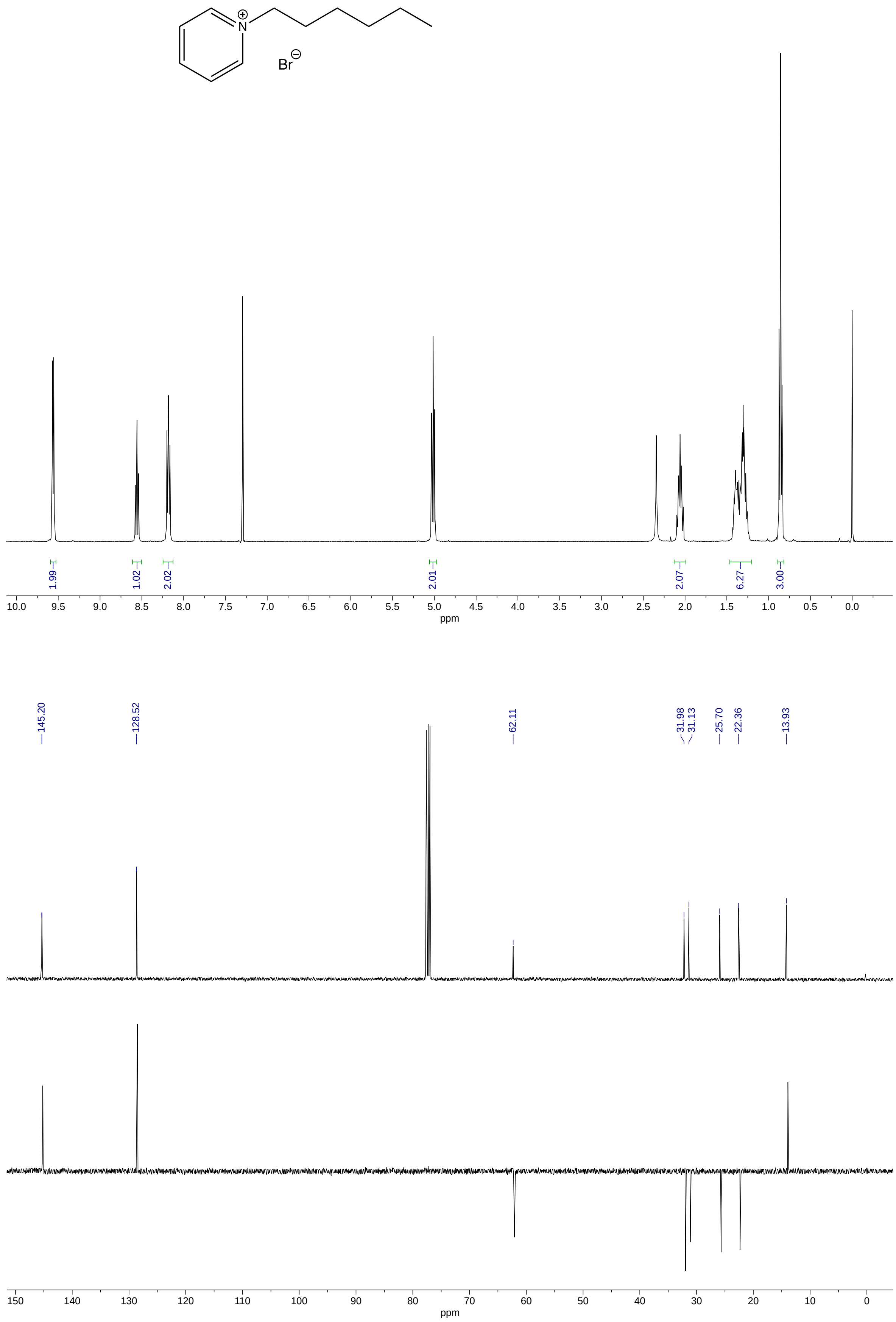

Figure S36. ^{1}H NMR **(a)**, ^{13}C NMR and DEPT135 **(b)** spectra of compound **6a**.

a)

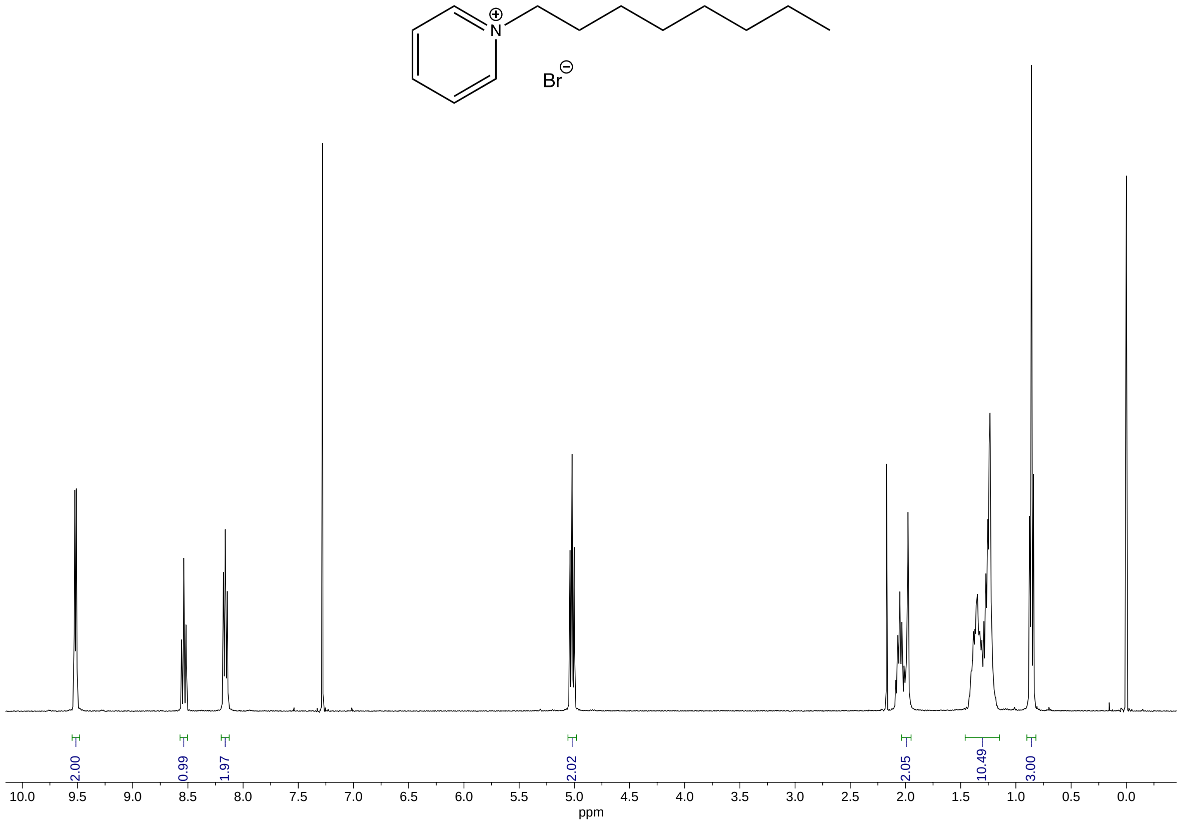

b)

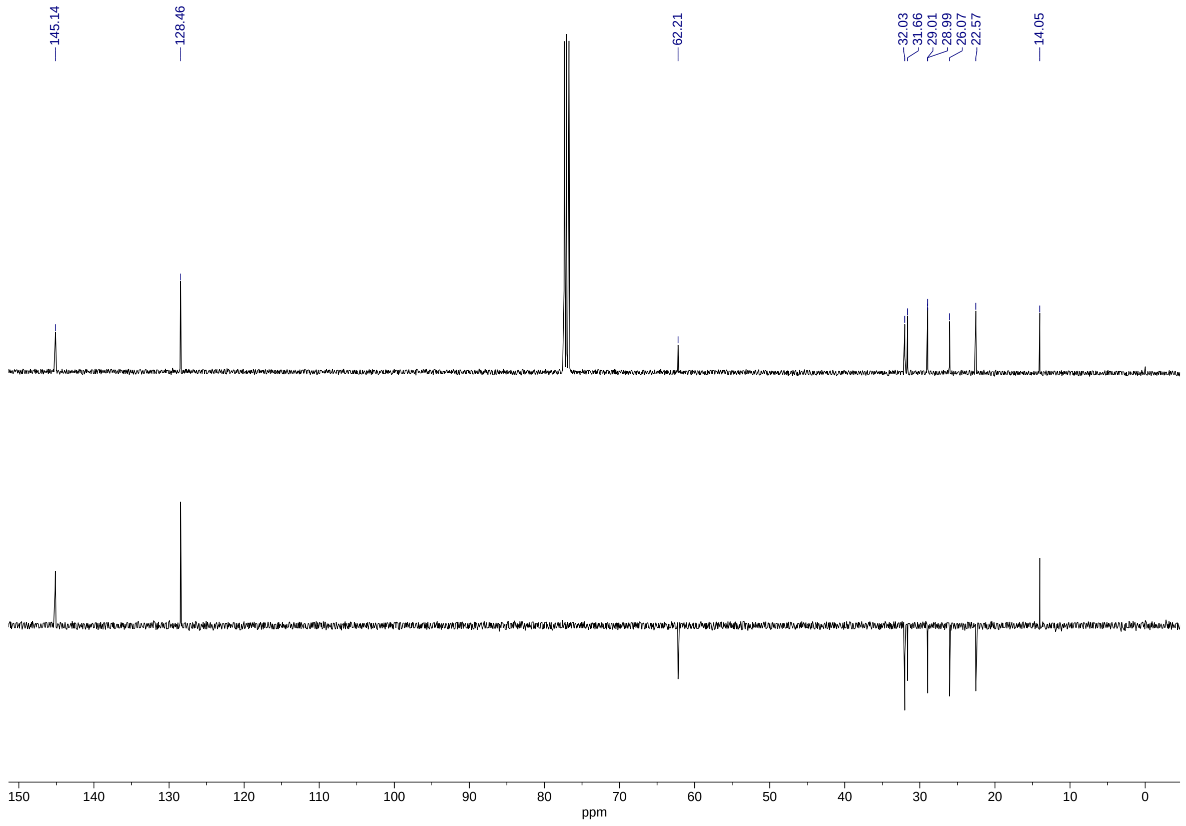

Figure S37. ^{1}H NMR **(a)**, ^{13}C NMR and DEPT135 **(b)** spectra of compound **6b**.

a)

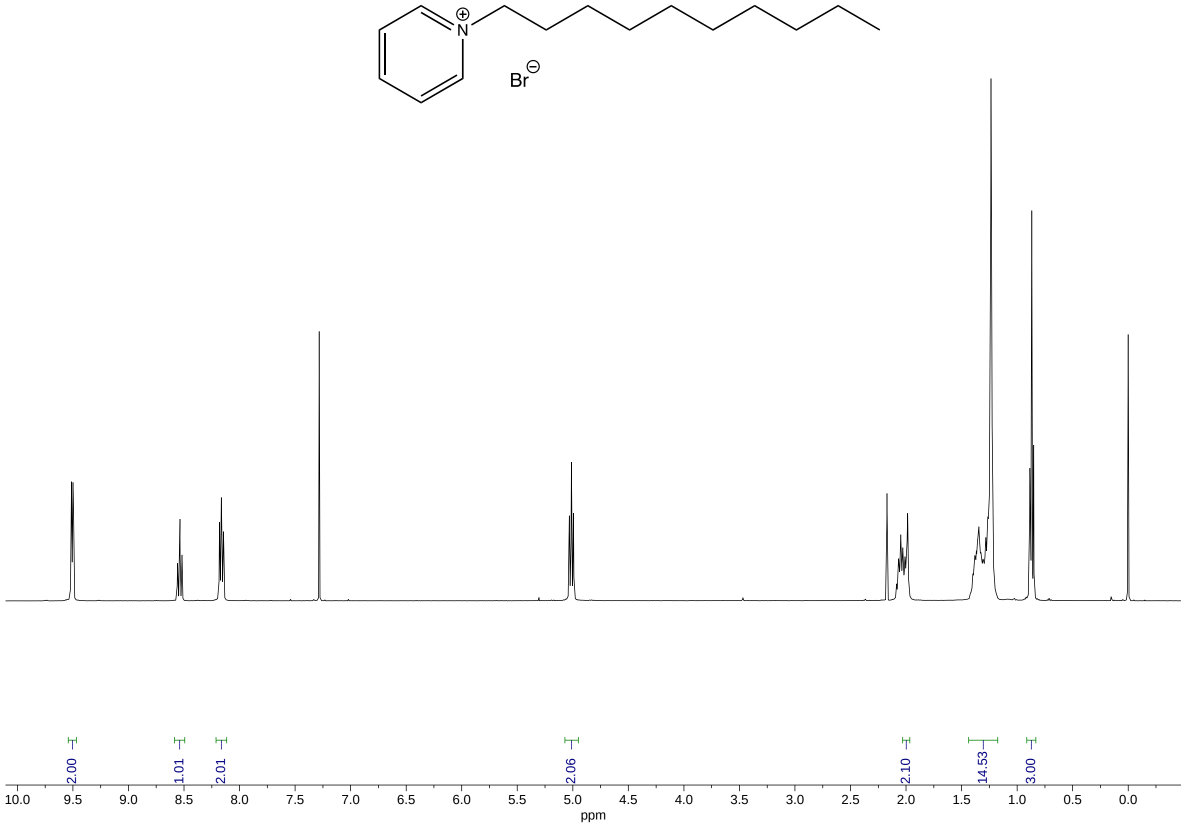

b)

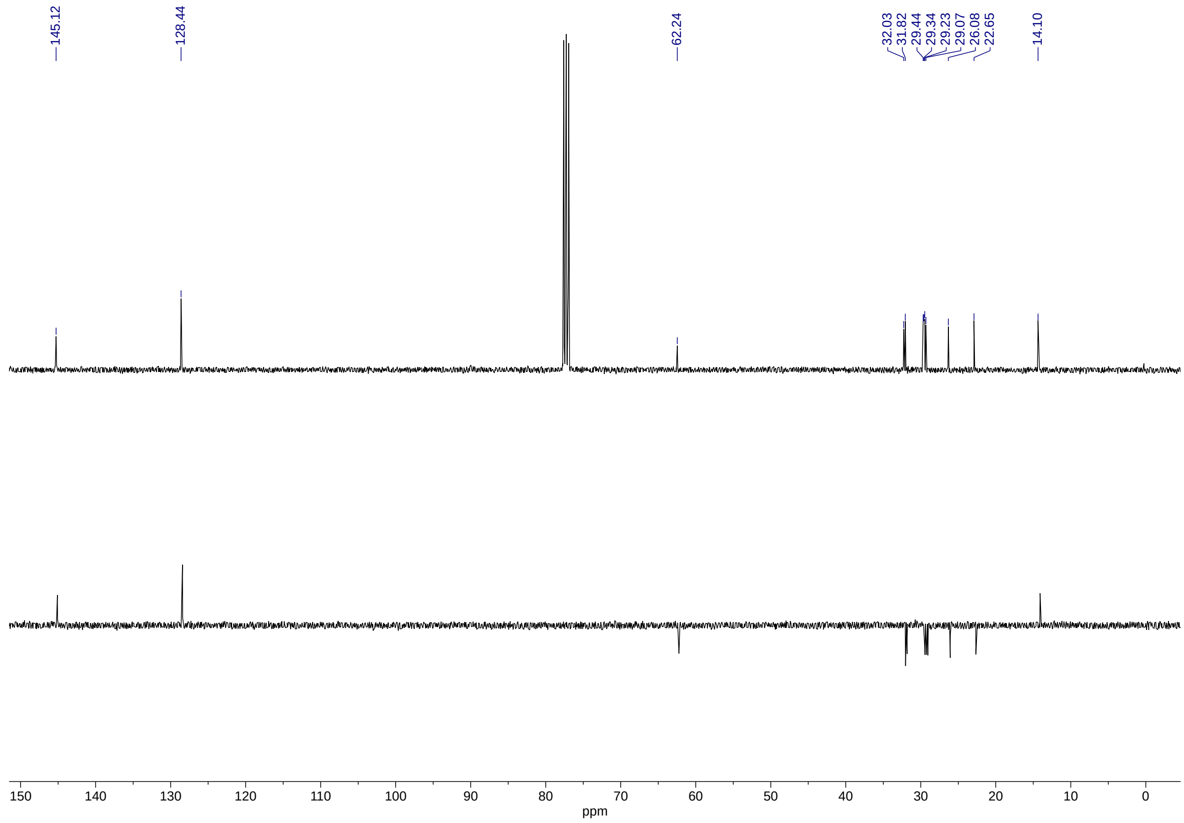

Figure S38. ^{1}H NMR **(a)**, ^{13}C NMR and DEPT135 **(b)** spectra of compound **6c**.

a)

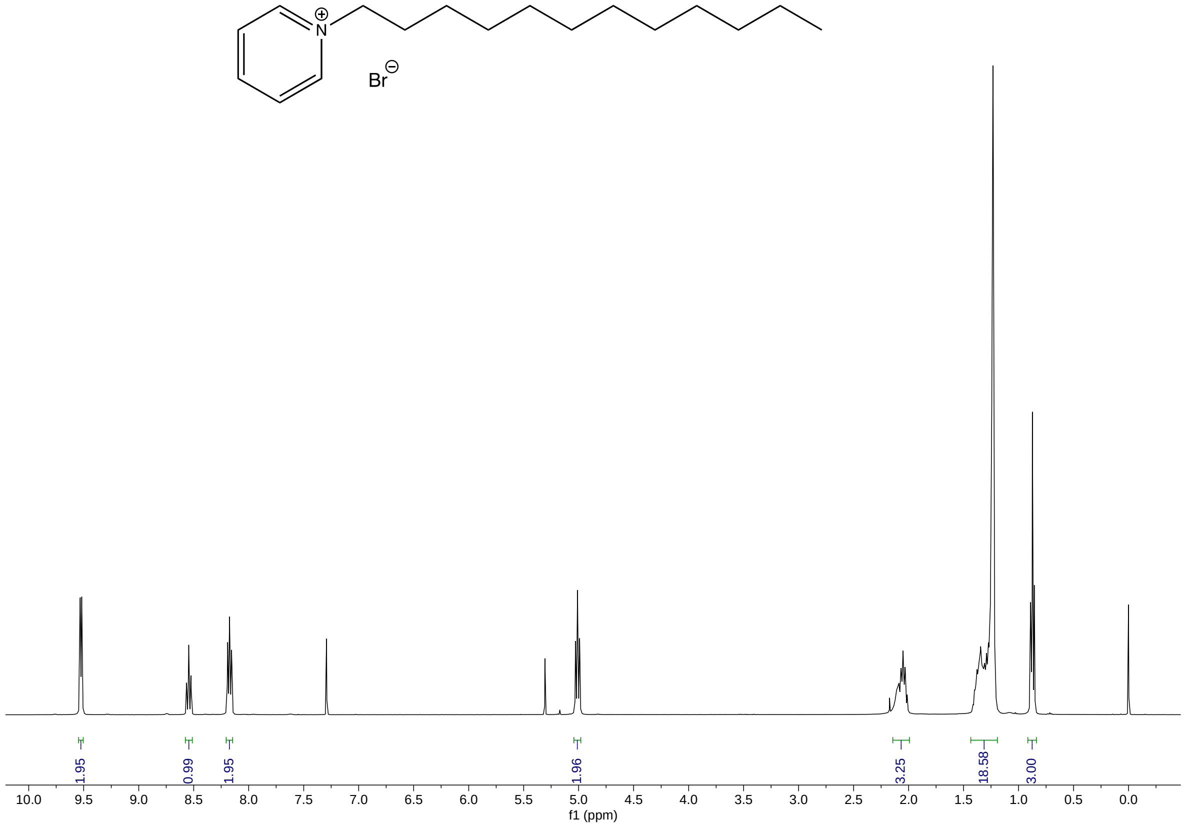

b)

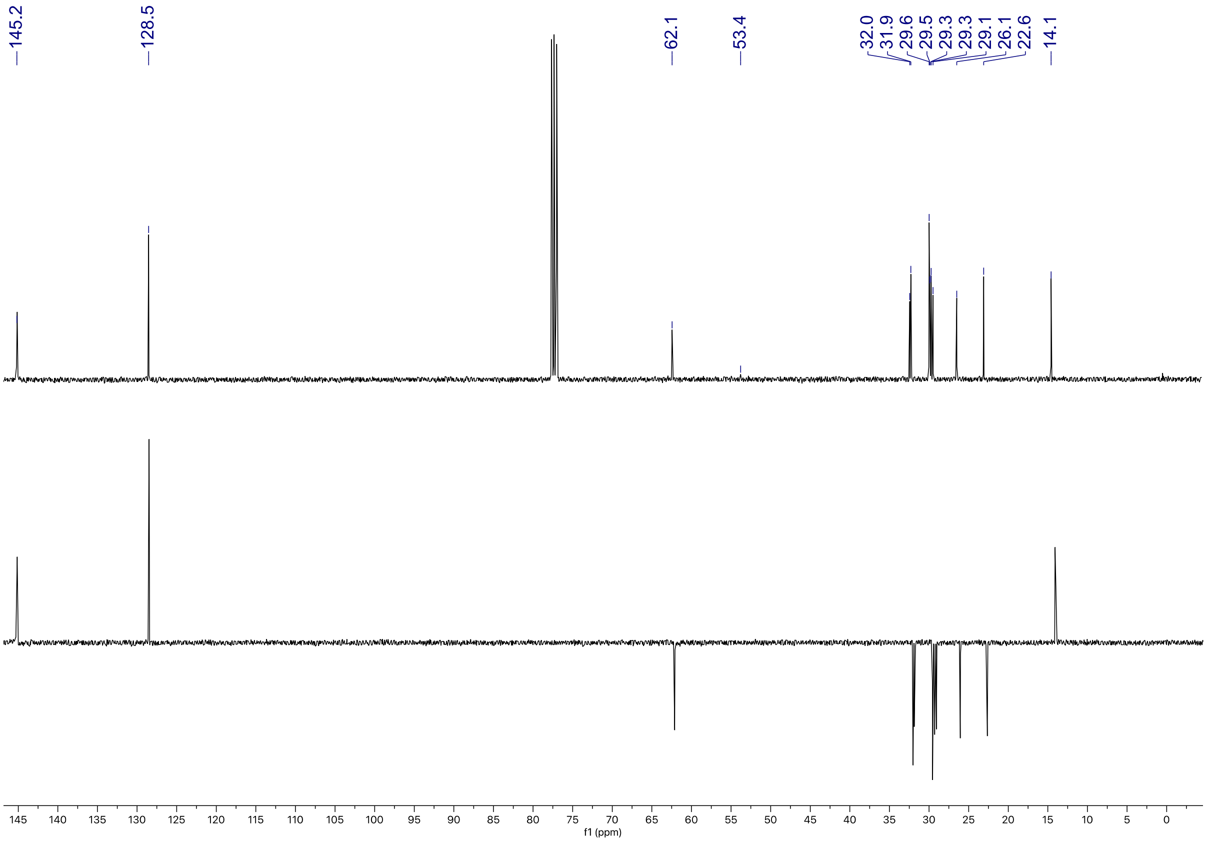

Figure S39. ^{1}H NMR **(a)**, ^{13}C NMR and DEPT135 **(b)** spectra of compound **6d**.

a)

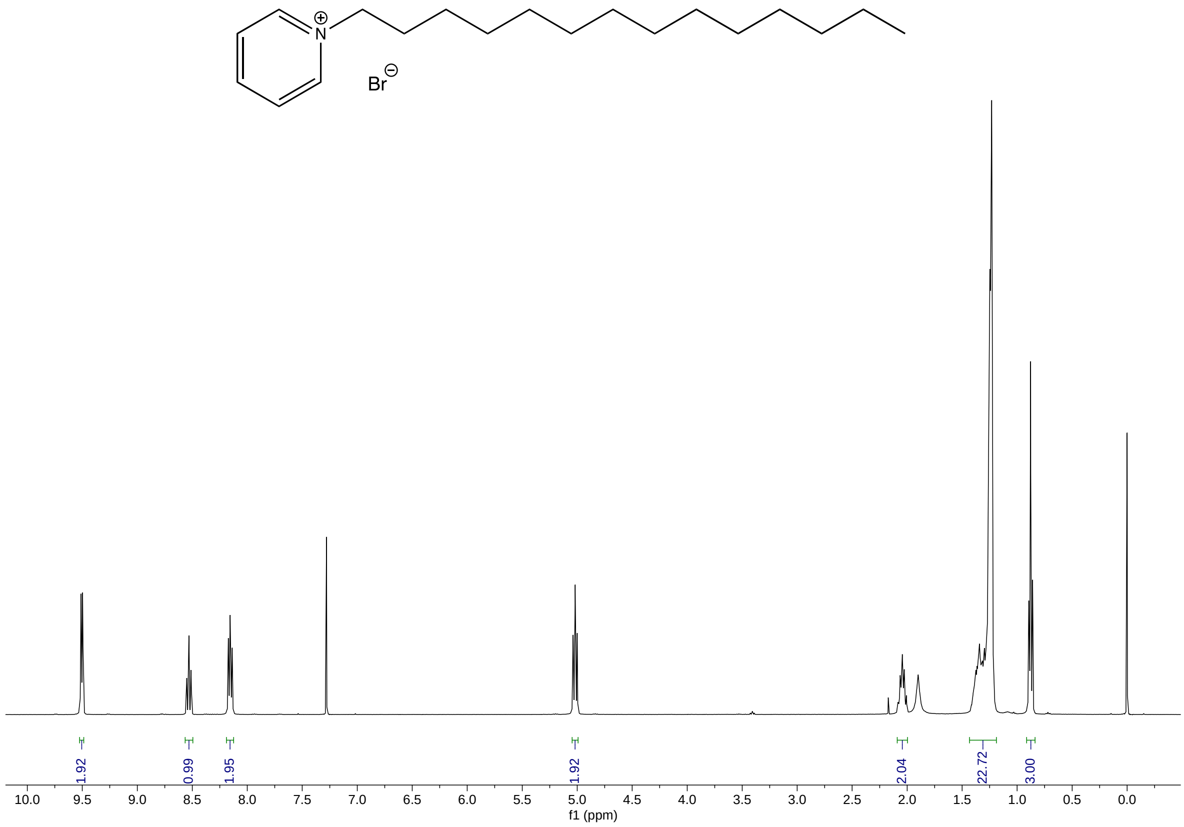

b)

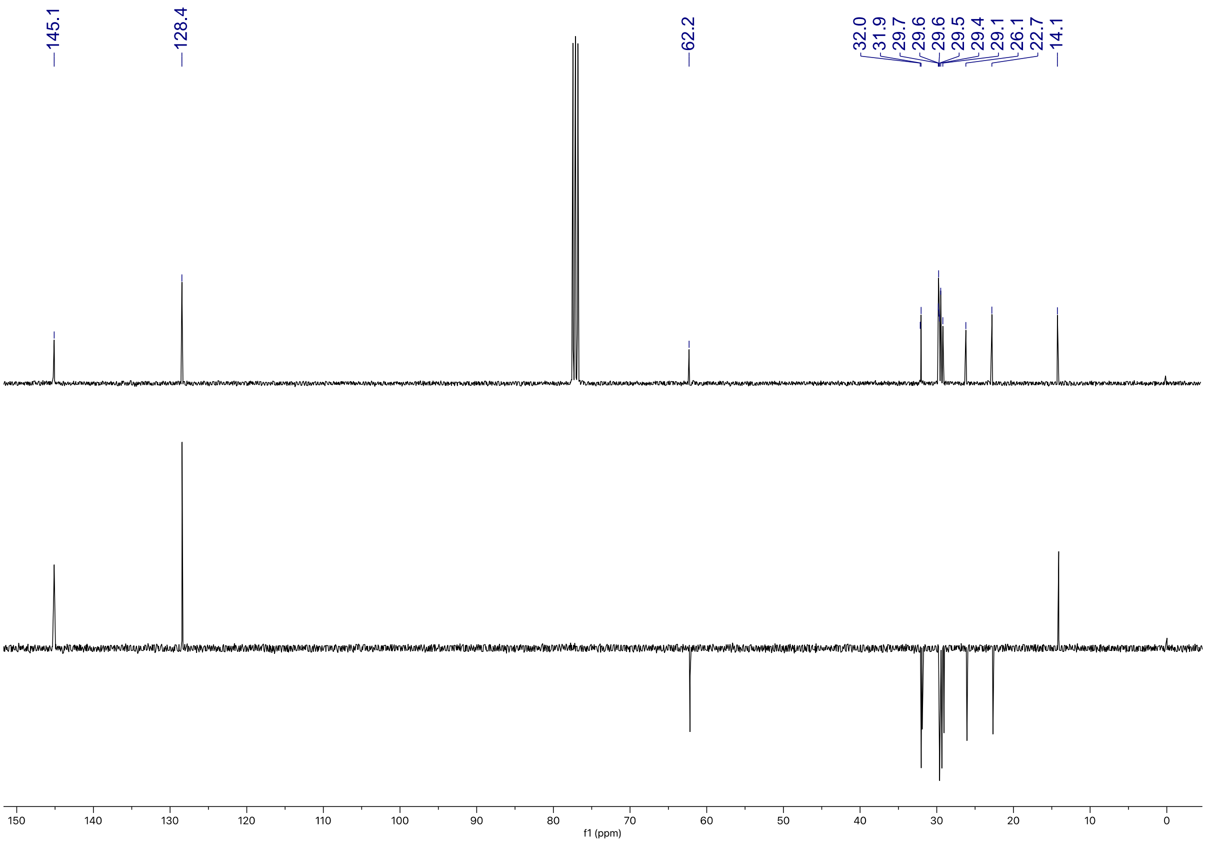

Figure S40. ^{1}H NMR **(a)**, ^{13}C NMR and DEPT135 **(b)** spectra of compound **6e**.

a)

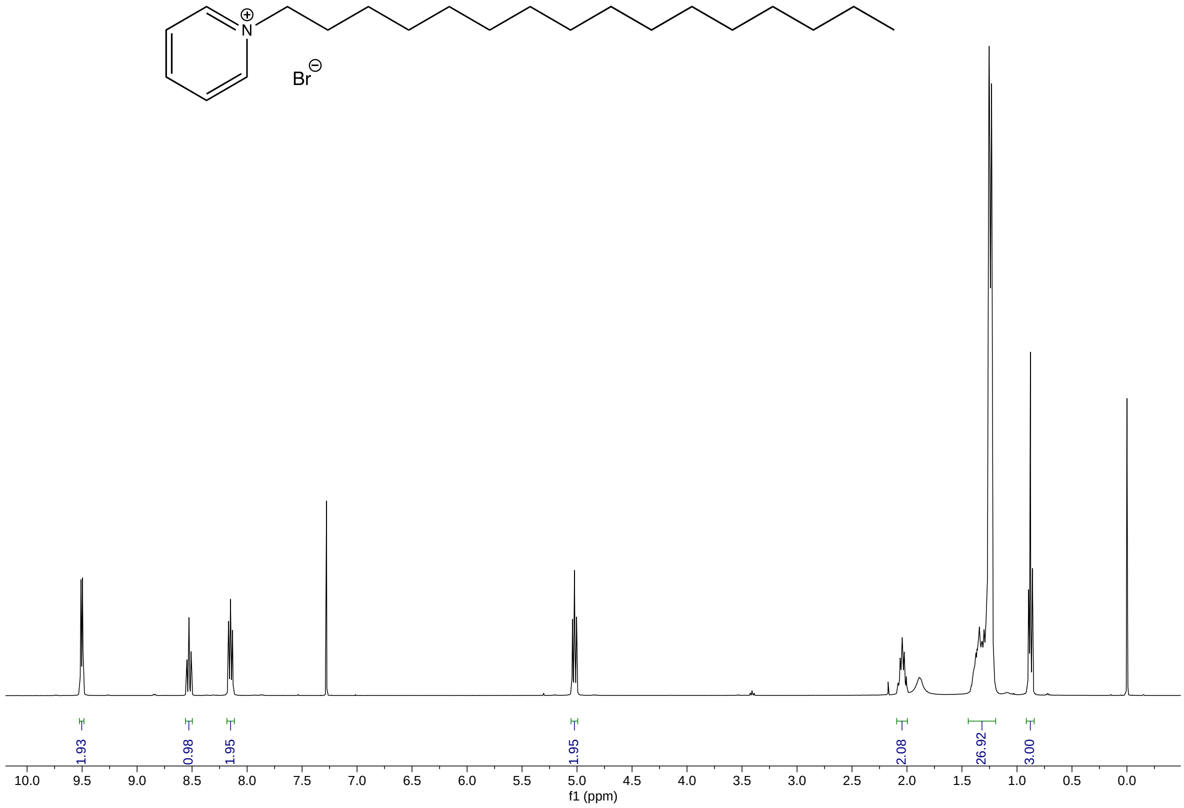

b)

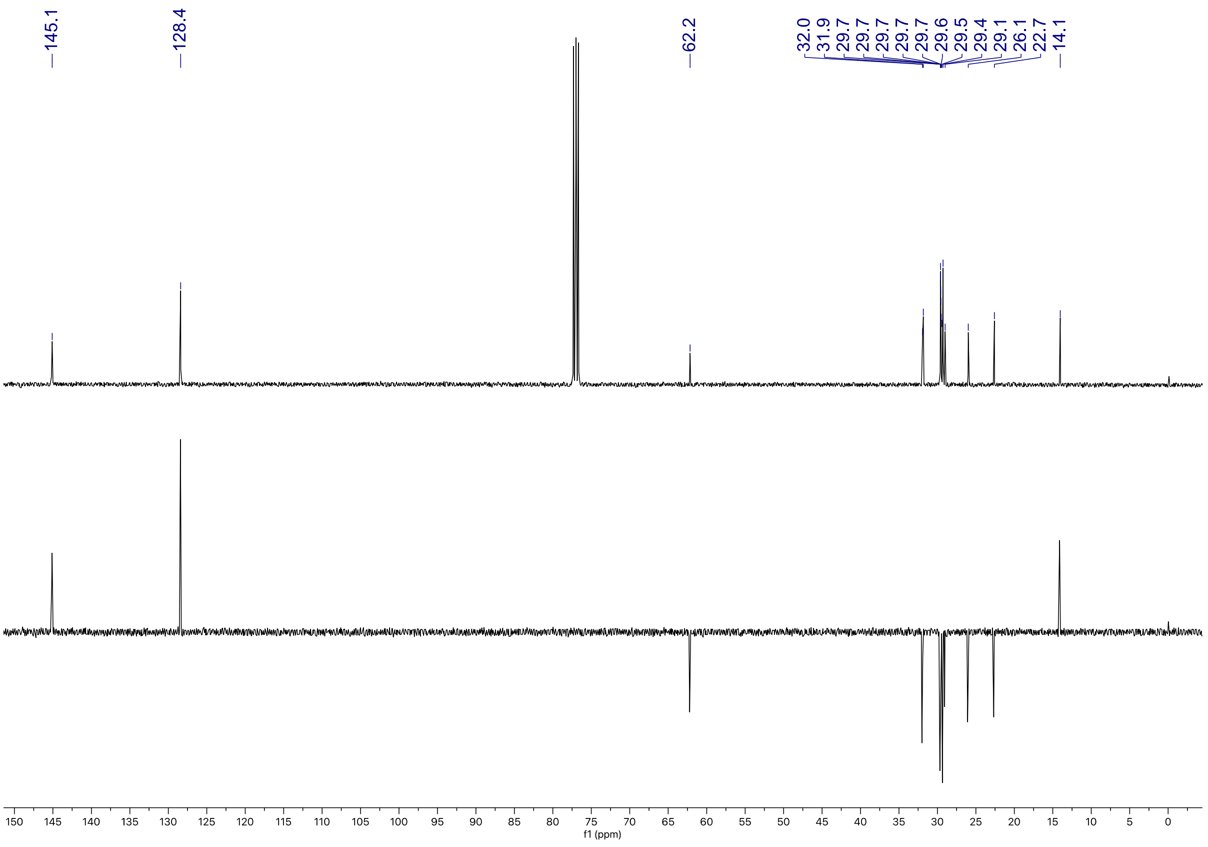

Figure S41. ^{1}H NMR **(a)**, ^{13}C NMR and DEPT135 **(b)** spectra of compound **6f**.

a)

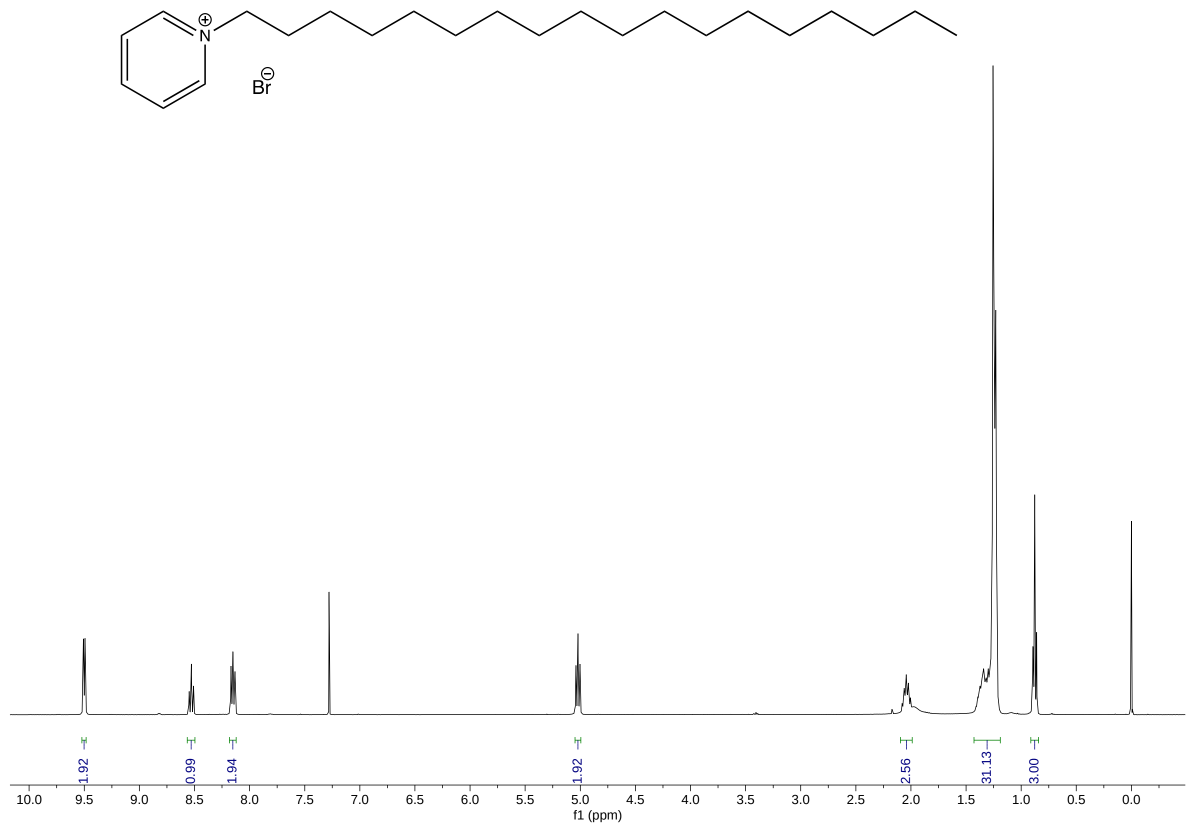

b)

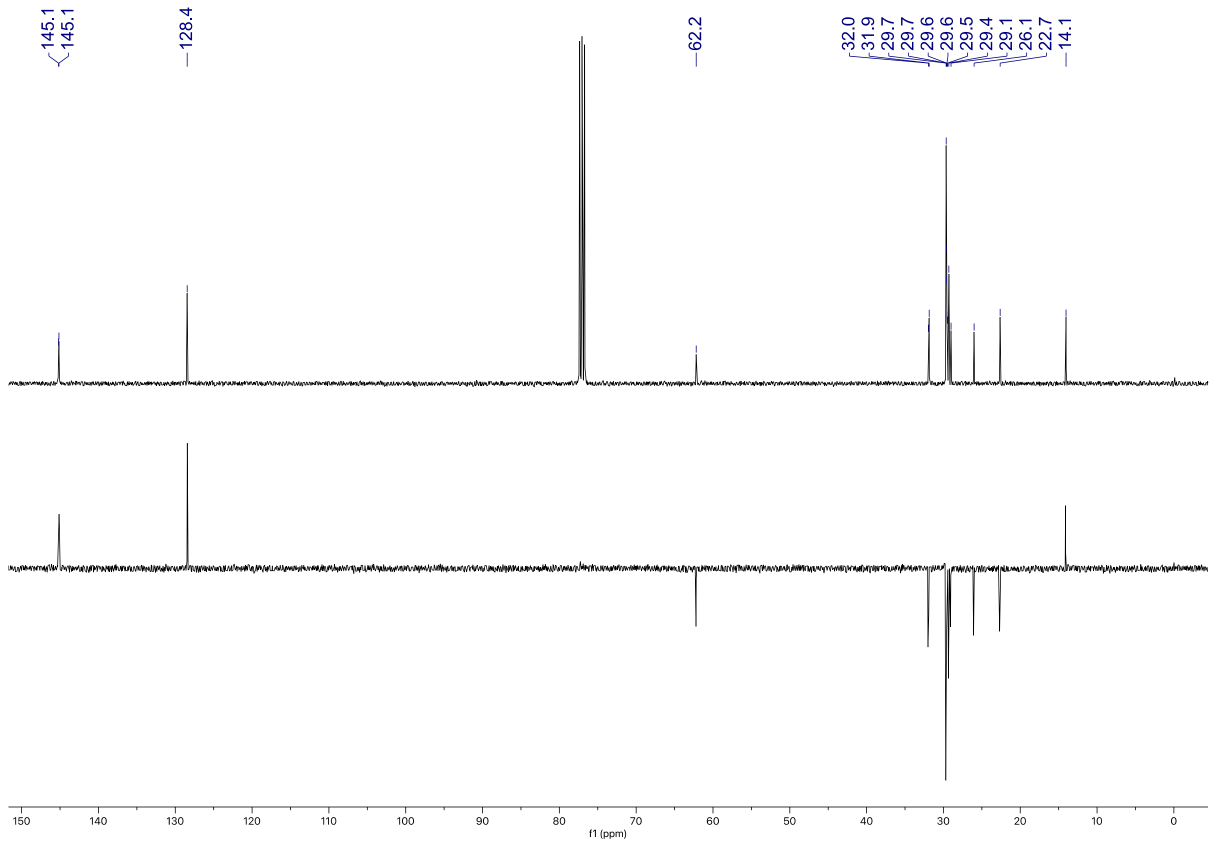

Figure S42. ^{1}H NMR **(a)**, ^{13}C NMR and DEPT135 **(b)** spectra of compound **6g**.

a)

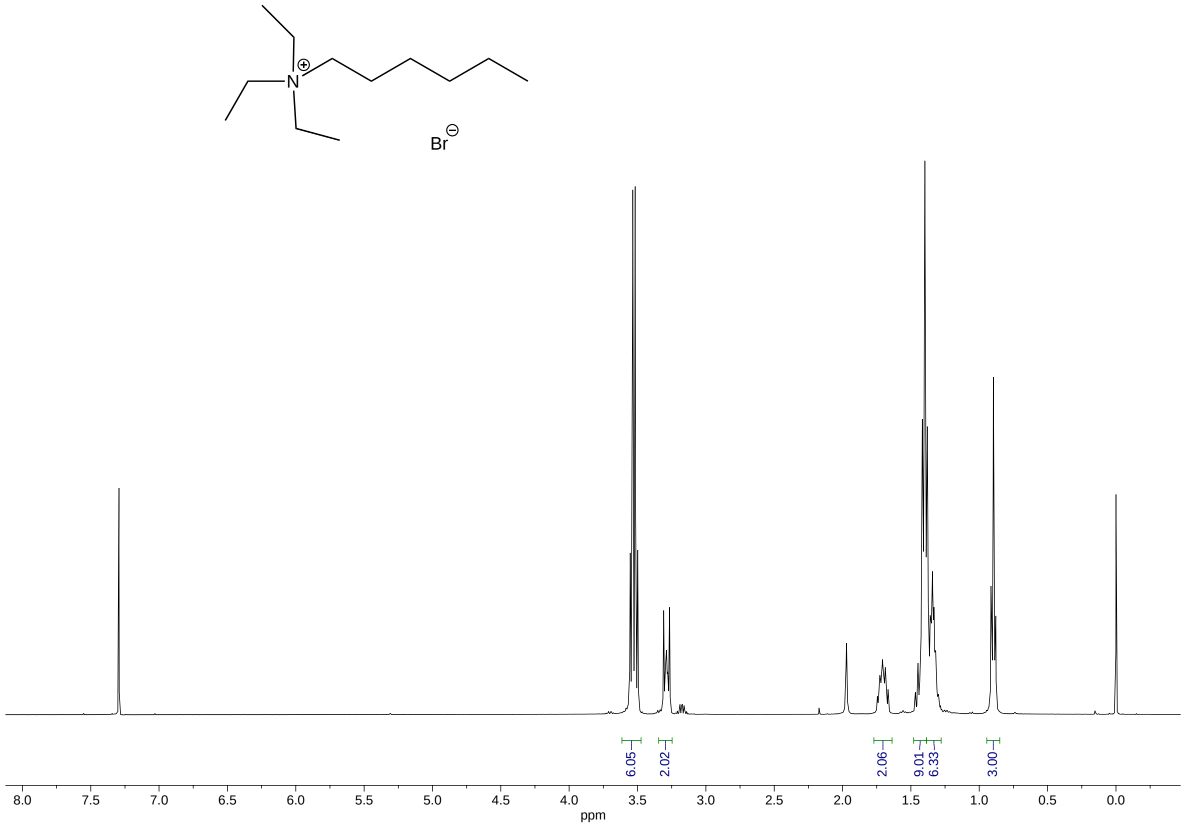

b)

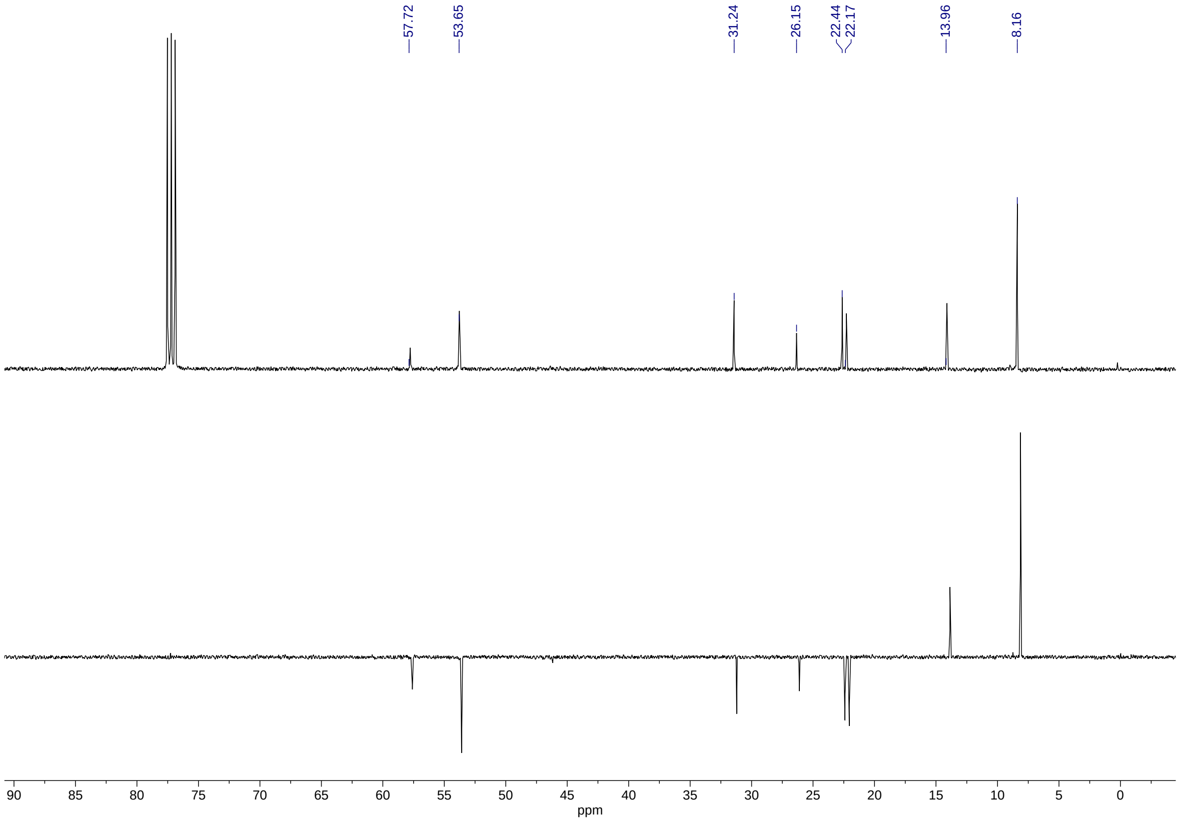

Figure S43. ^{1}H NMR **(a)**, ^{13}C NMR and DEPT135 **(b)** spectra of compound **7a**.

a)

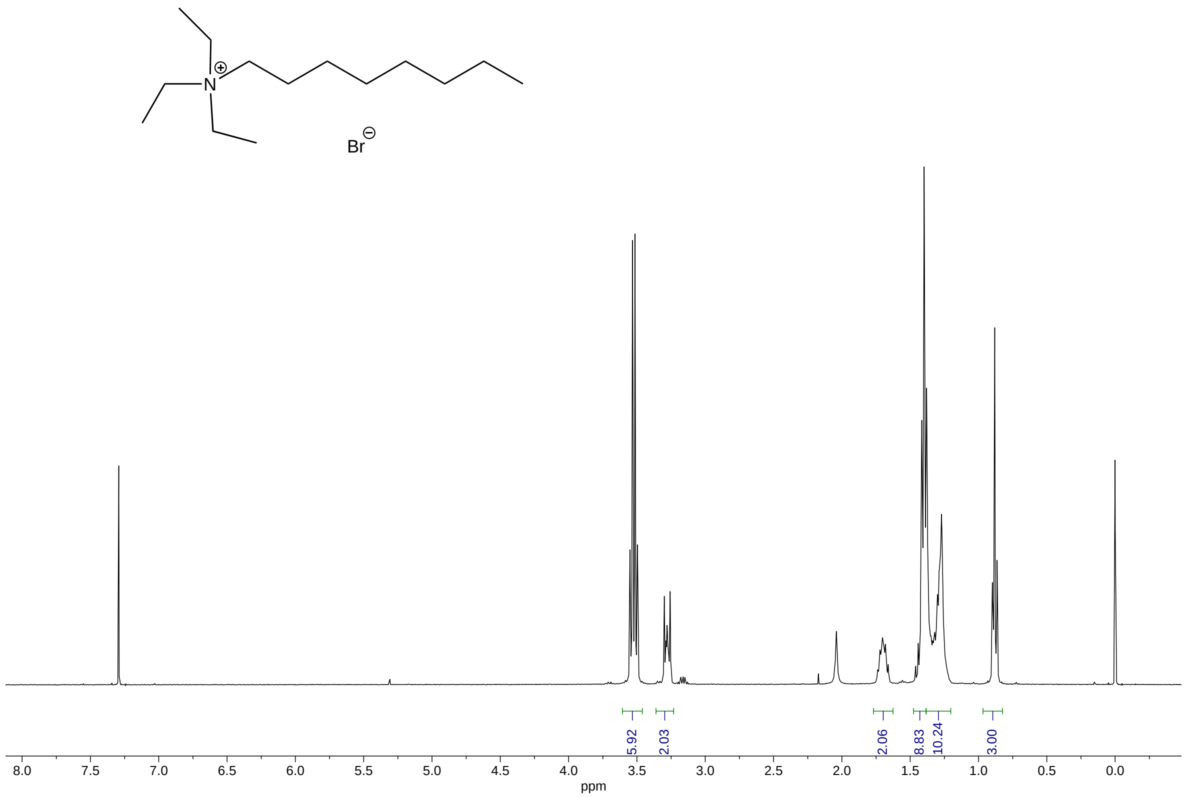

b)

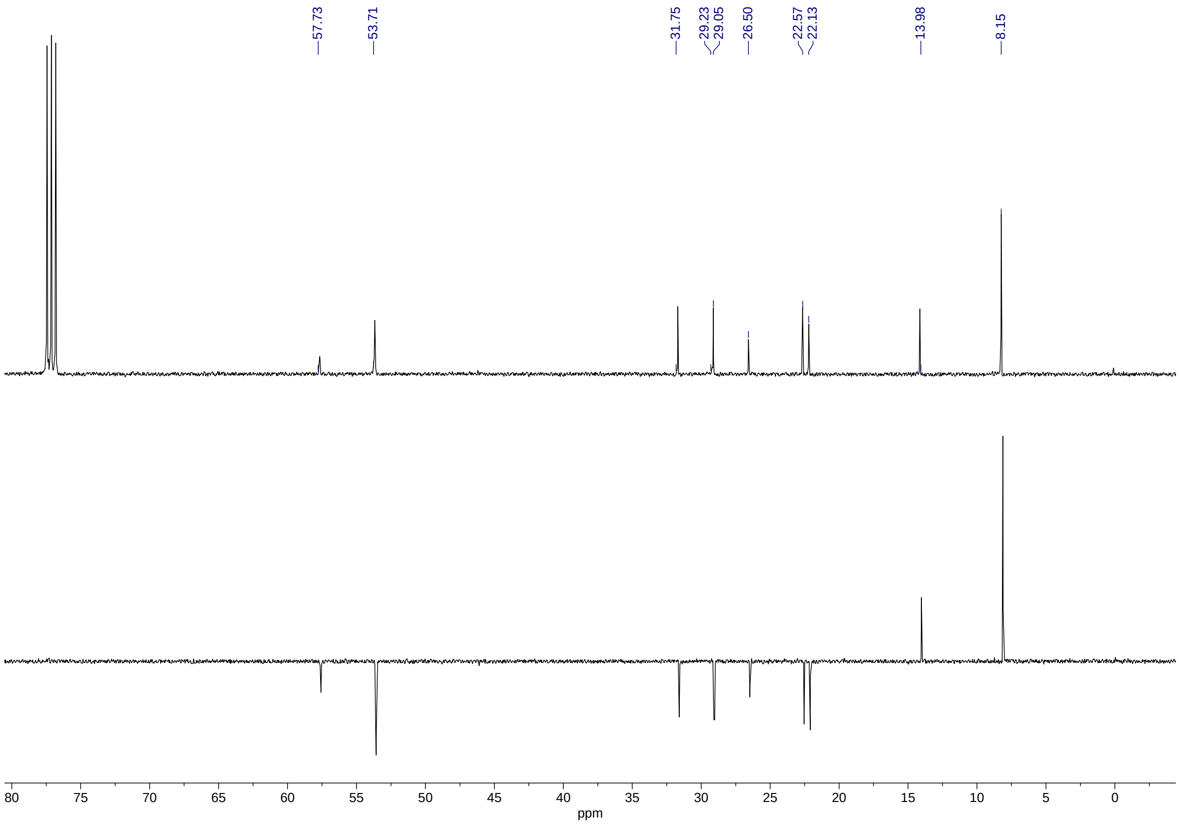

Figure S44. ^{1}H NMR **(a)**, ^{13}C NMR and DEPT135 **(b)** spectra of compound **7b**.

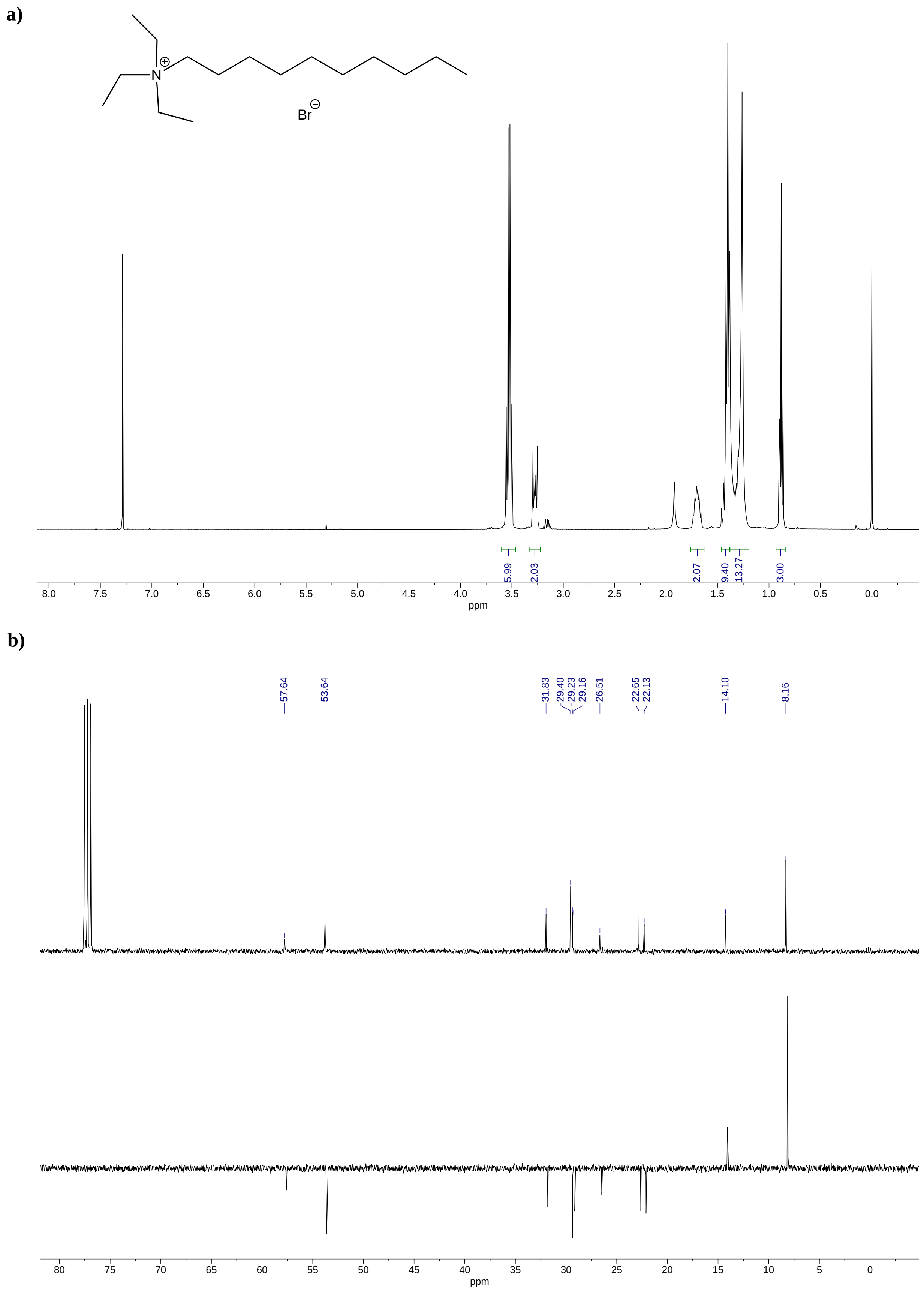

Figure S45. ^{1}H NMR **(a)**, ^{13}C NMR and DEPT135 **(b)** spectra of compound **7c**.

a)

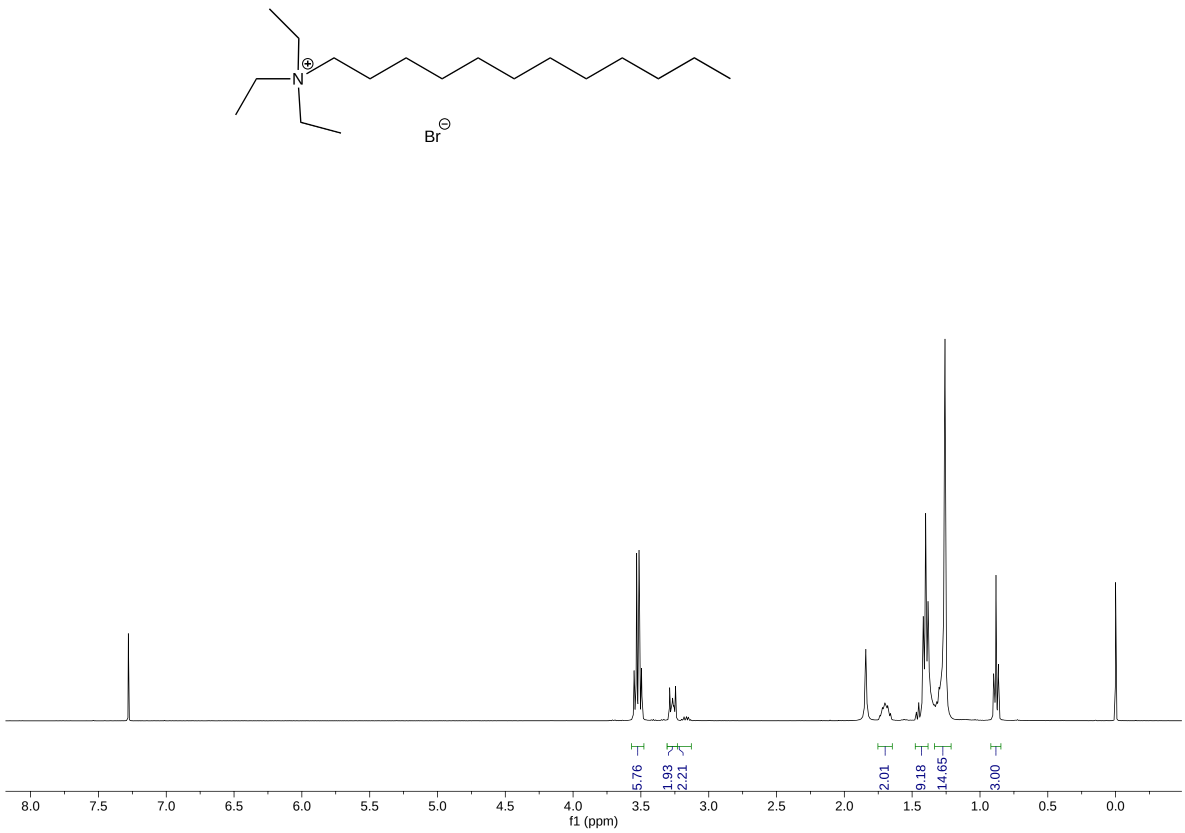

b)

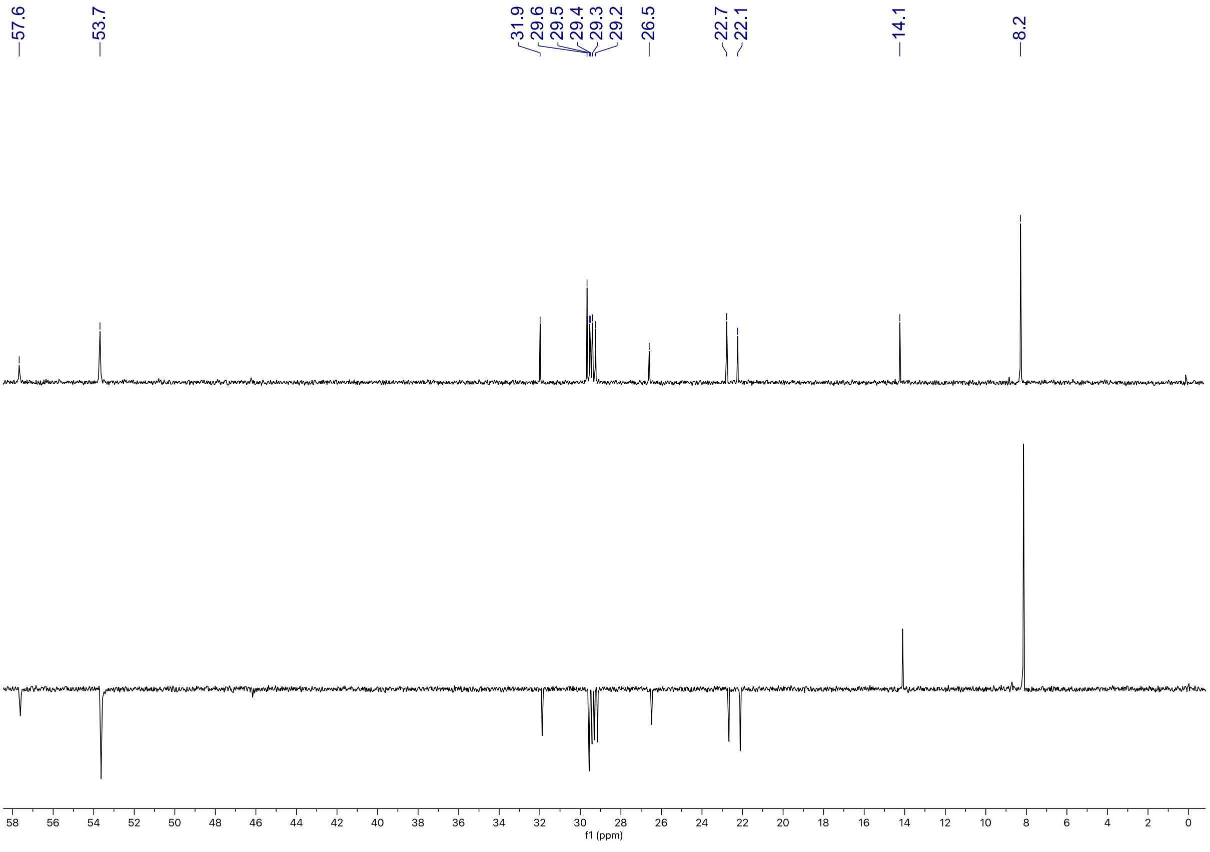

Figure S46. ^{1}H NMR **(a)**, ^{13}C NMR and DEPT135 **(b)** spectra of compound **7d**.

a)

b)

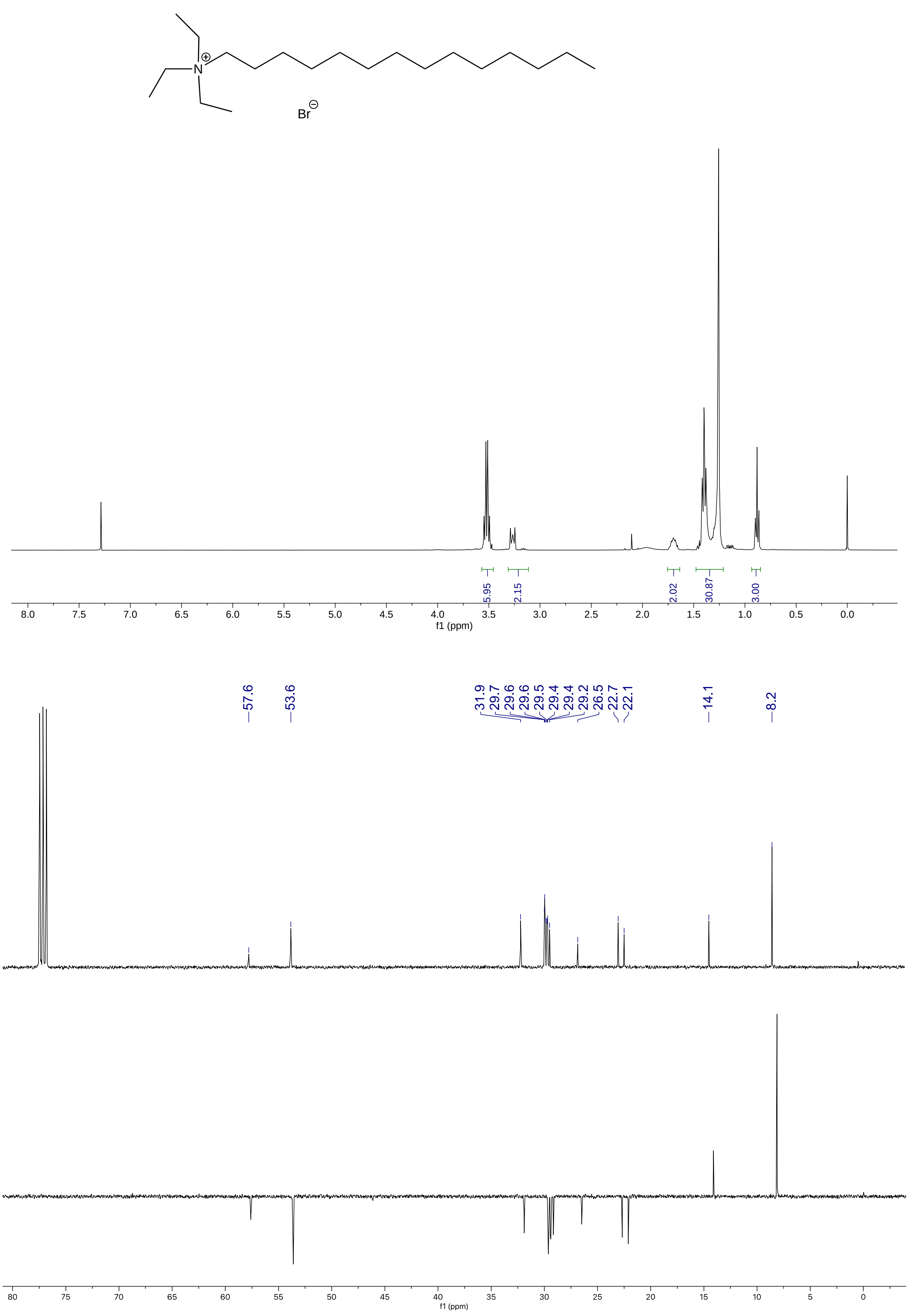

Figure S47. ^{1}H NMR **(a)**, ^{13}C NMR and DEPT135 **(b)** spectra of compound **7e**.

a)

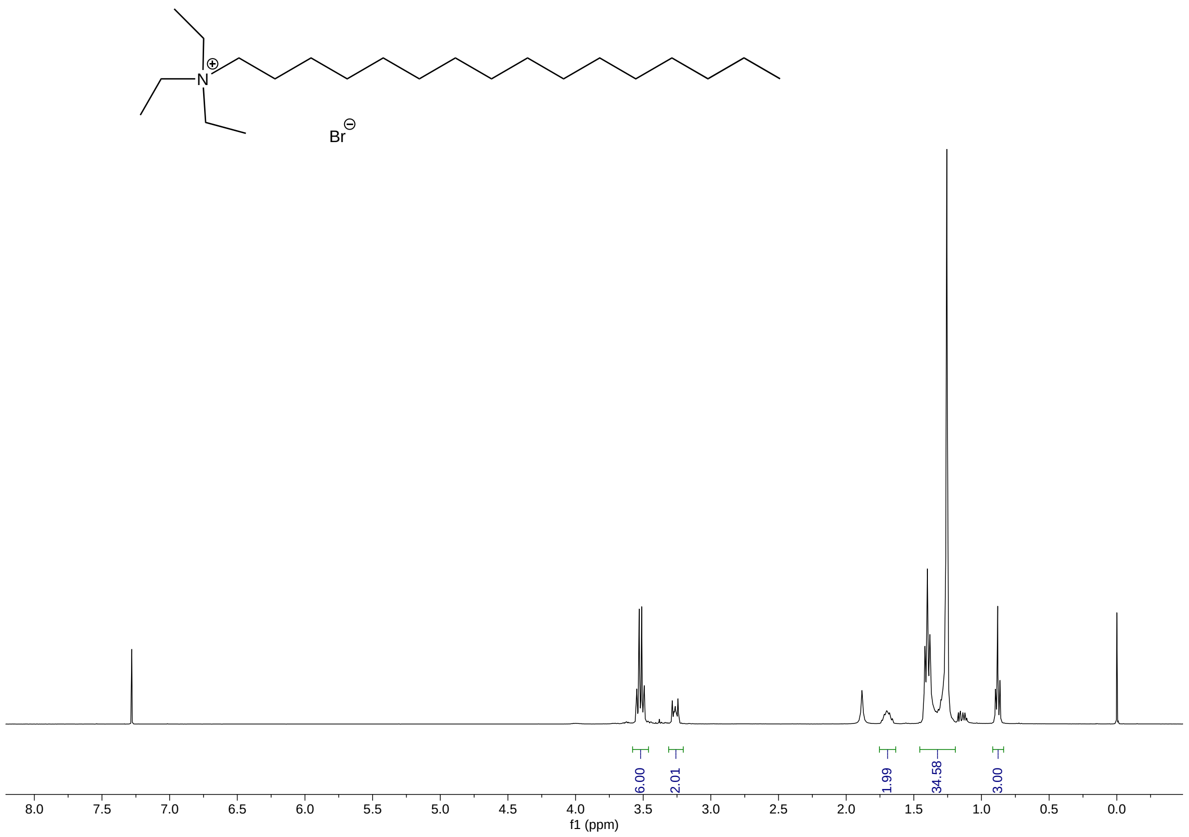

b)

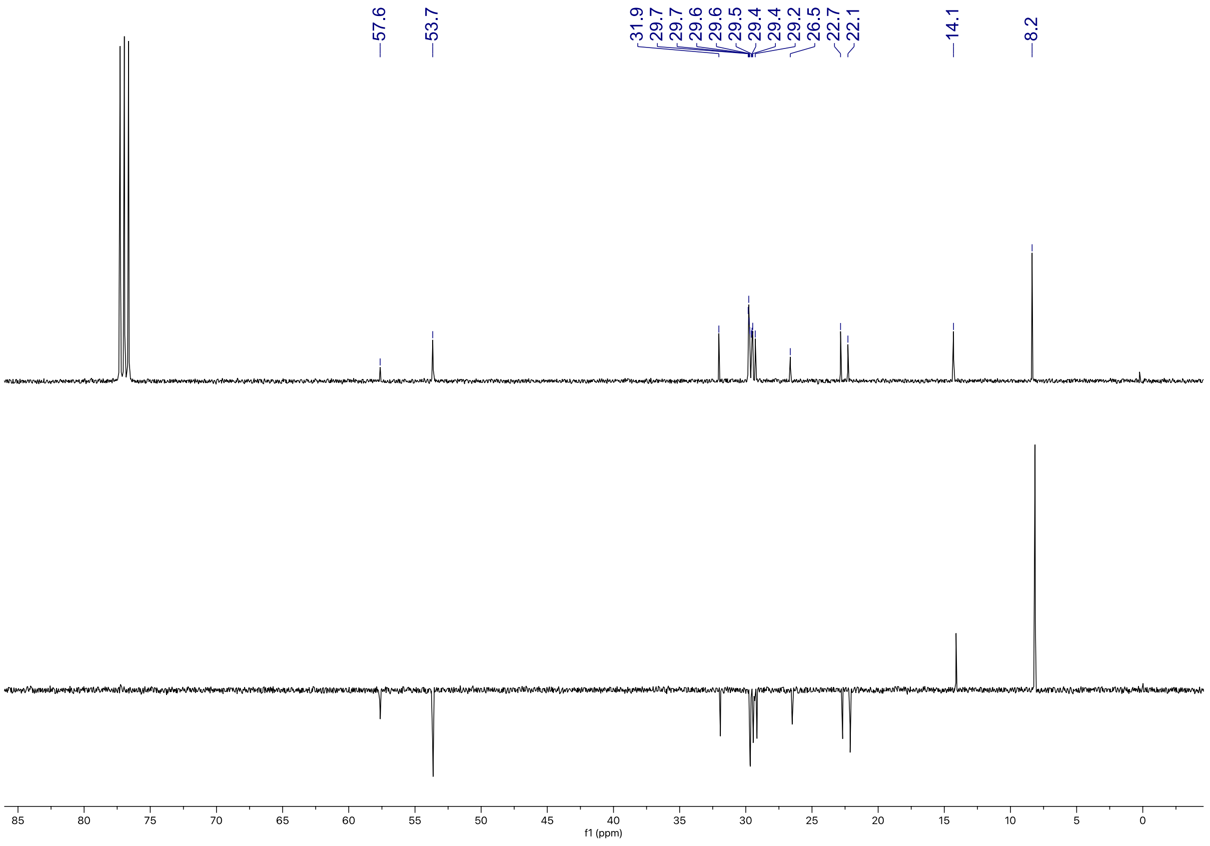

Figure S48. ^{1}H NMR **(a)**, ^{13}C NMR and DEPT135 **(b)** spectra of compound **7f**.

a)

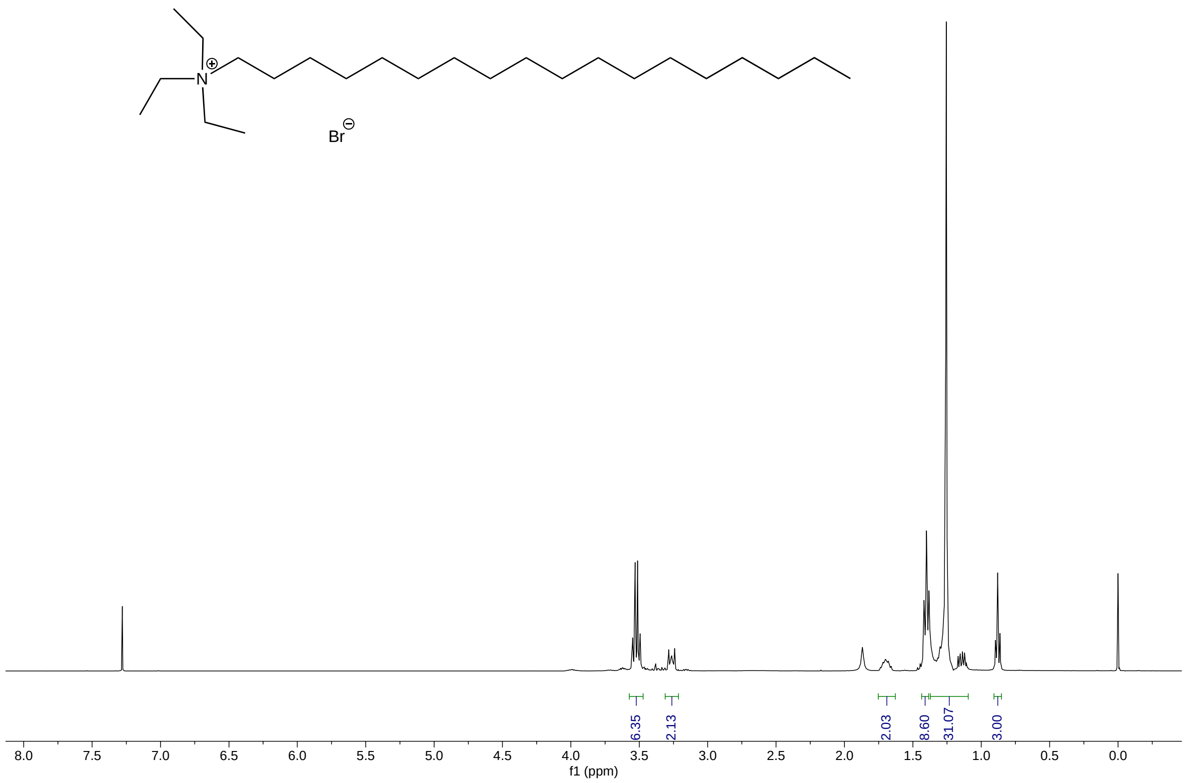

b)

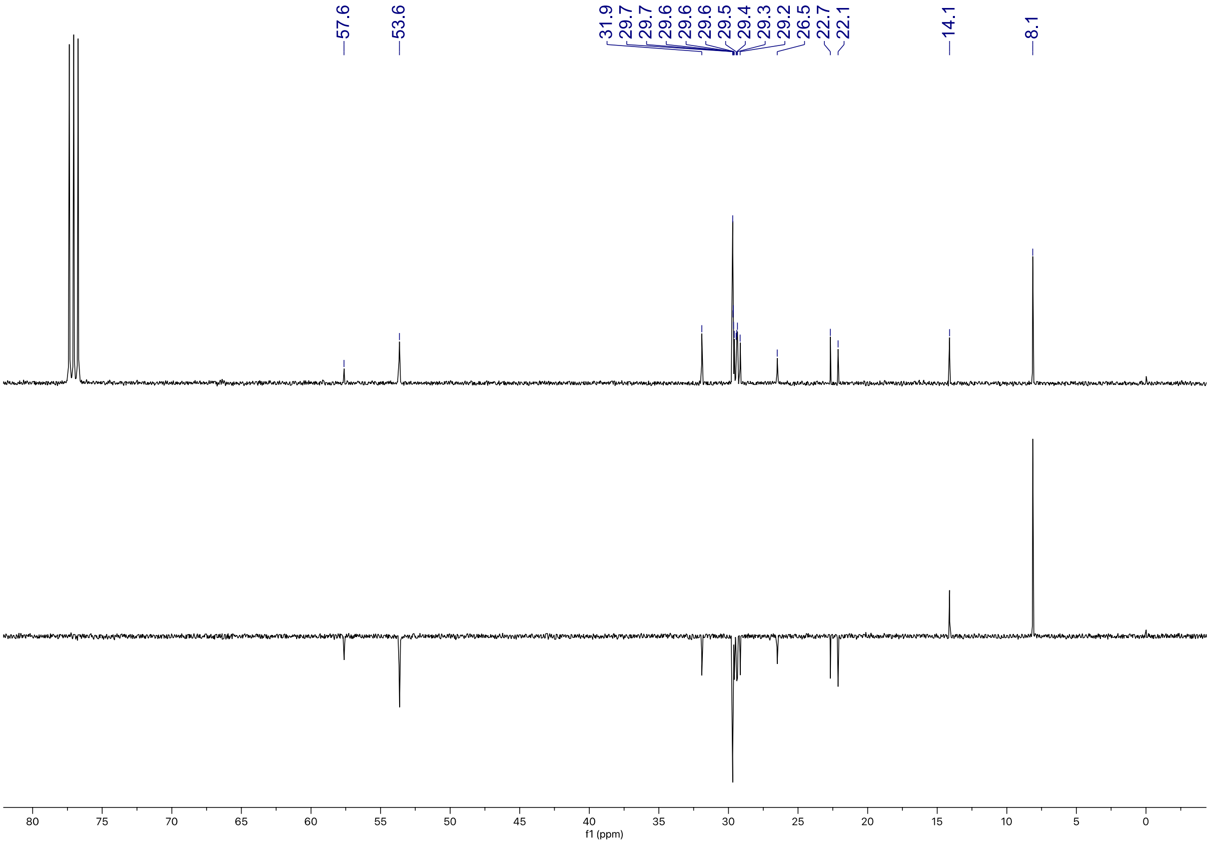

Figure S49. ^{1}H NMR **(a)**, ^{13}C NMR and DEPT135 **(b)** spectra of compound **7g**.

1.2 Evaluation of the Chromatographic Hydrophobicity Index

Table S1. Retention times (t_R) of the standard mixture obtained by LC/UV at pH 2.6. CHI_0 values at pH 2.6.

Compound	t_R (min)	CHI_0 pH 2.6
Benzimidazole	4.141	6.300
Theophylline	4.532	17.90
Paracetamol	4.656	18.77
Caffeine	4.823	23.41
Colchicine	5.792	43.90
Carbamazepine	6.434	60.42
Indole	7.076	72.10
Propiophenone	7.421	77.40
Butyrophenone	7.901	87.30
Valerophenone	8.326	96.40
Heptanophenone	9.092	112.1

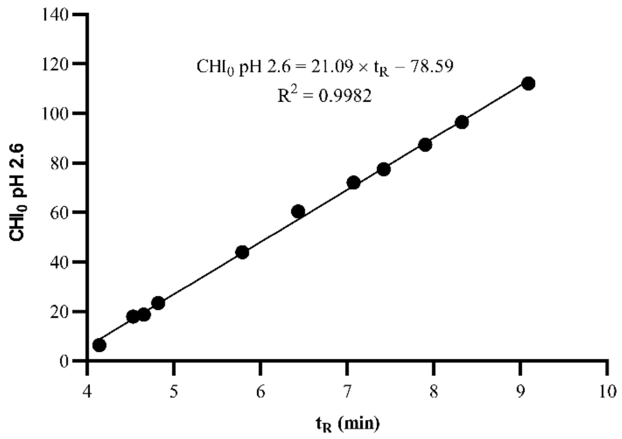

Figure S50. Linear correlation obtained by plotting the retention times (t_R) of each of the individual standard mixture compounds against the CHI values at pH 2.6 (CHI_0).
